# Reduced chromatin accessibility underlies gene expression differences in homologous chromosome arms of diploid *Aegilops tauschii* and hexaploid wheat

**DOI:** 10.1093/gigascience/giaa070

**Published:** 2020-06-20

**Authors:** Fu-Hao Lu, Neil McKenzie, Laura-Jayne Gardiner, Ming-Cheng Luo, Anthony Hall, Michael W Bevan

**Affiliations:** Department Cell and Developmental Biology, John Innes Centre, Norwich Research Park, Norwich NR4 7UH, UK; Department Cell and Developmental Biology, John Innes Centre, Norwich Research Park, Norwich NR4 7UH, UK; Earlham Institute, Norwich Research Park, Norwich Research Park, Norwich NR4 7UZ, UK; Department of Plant Sciences, University of California, 1 Shields Avenue, Davis, CA 95616, USA; Earlham Institute, Norwich Research Park, Norwich Research Park, Norwich NR4 7UZ, UK; Department Cell and Developmental Biology, John Innes Centre, Norwich Research Park, Norwich NR4 7UH, UK

**Keywords:** *Triticum aestivum*, wheat, chr3DL, synteny, ATAC, gene expression in polyploids

## Abstract

**Background:**

Polyploidy is centrally important in the evolution and domestication of plants because it leads to major genomic changes, such as altered patterns of gene expression, which are thought to underlie the emergence of new traits. Despite the common occurrence of these globally altered patterns of gene expression in polyploids, the mechanisms involved are not well understood.

**Results:**

Using a precisely defined framework of highly conserved syntenic genes on hexaploid wheat chromosome 3DL and its progenitor 3 L chromosome arm of diploid *Aegilops tauschii*, we show that 70% of these gene pairs exhibited proportionately reduced gene expression, in which expression in the hexaploid context of the 3DL genes was ∼40% of the levels observed in diploid *Ae tauschii*. Several genes showed elevated expression during the later stages of grain development in wheat compared with *Ae tauschii*. Gene sequence and methylation differences probably accounted for only a few cases of differences in gene expression. In contrast, chromosome-wide patterns of reduced chromatin accessibility of genes in the hexaploid chromosome arm compared with its diploid progenitor were correlated with both reduced gene expression and the imposition of new patterns of gene expression.

**Conclusions:**

Our pilot-scale analyses show that chromatin compaction may orchestrate reduced gene expression levels in the hexaploid chromosome arm of wheat compared to its diploid progenitor chromosome arm.

## Background

Polyploidy arises from the duplication or fusion of genomes and has occurred frequently in the lineages of many organisms, from fish to flowering plants [1]. The ancestral flowering plant lineage has undergone ≥2 genome duplication events, with subsequent multiple genome duplication events in different lineages [2]. Radical alterations of gene expression patterns are commonly observed consequences of polyploidization. In newly formed allotetraploids of *Arabidopsis arenosa* and *Arabidopsis thaliana* ∼5% of genes were expressed at different levels than in the parental lines [3]. In *Tragopogon* allopolyploids, ∼76% of homoeologous genes displayed additive expression (the average of expression measured in each parental line), and ∼20% of transcripts exhibited non-additive expression in which gene expression levels varied from the average of parents [4]. Over longer time scales, a tendency of homoeologous gene expression from one parental genome to dominate over the other has been observed in several polyploids, including cotton, Brassicas, and maize [5]. The mechanisms underlying these genome-scale changes in gene expression are poorly understood.

Many crop species have undergone relatively recent polyploid events prior to and during domestication, leading to new traits and improved performance [6]. Large genomic segments such as entire chromosomes from related species are also added to crop varieties to introduce new traits [7]. Understanding how these large-scale genomic changes influence genome stability and gene expression, and how they give rise to improved performance in crops, is therefore centrally important from both practical and research perspectives. Several hypotheses have been proposed to explain genomic interactions in hybrids and polyploids, ranging from complementation of differing alleles, misregulation of gene expression, epigenetic changes, and the activities of transposable elements (TEs) [8–10].

The wheat group of the Triticeae is characterized by stable tetraploid and hexaploid species that exhibit greater diversity, adaptability, and potential for domestication than their diploid progenitors. Multiple types of genomic changes, including altered expression patterns of genes and TEs, and epigenetic changes, have been proposed to contribute to this “genomic plasticity” [11]. The hexaploid bread wheat genome (*Triticum aestivum*) arose from the very recent integration of the diploid *Aegilops tauschii* DD genome into a tetraploid *Triticum turgidum* AABB genome [12]. The 3 component genomes are very closely related, sharing common ancestry in the Triticeae lineage ∼6.5 million years ago. In newly synthesized allohexaploid wheat [13] and tetraploid AADD and S′S′AA (where S′S′ is *Aegilops longissima*) [14] wheat lines between 60% and 80% of genes were additively expressed, suggesting a dynamic re-adjustment of gene expression patterns as a consequence of allopolyploidy. Rapid asymmetric changes in short RNA, histone methylation, and gene expression in the 2 allotetraploids are thought to contribute to genome-biased gene expression and the activation of TE transcription [14]. RNA-sequencing (RNA-seq) analyses of newly formed allotriploid ABD and stable allohexaploid AABBDD lines generated from *T. turgidum* and *Ae tauschii* showed rapid and extensive changes in gene expression in triploid tissues that were partly restored upon genome duplication [15]. The overall very high conservation of sequences and gene order of D genome chromosomes in diploid *Ae tauschii* and hexaploid wheat [16] provides an important opportunity to assess differences between diploid and hexaploid states of very similar chromosomes. Here we analyse gene expression, DNA methylation, and chromatin accessibility of a set of well-defined syntenic genes from diploid chromosome 3 L of *Ae tauschii* and hexaploid chromosome 3DL of bread wheat and show that reduced chromatin accessibility underlies large-scale changes in gene expression between diploid and hexaploid states.

## Data Description

### Plant materials

The Paragon elite wheat (*Triticum aestivum*, NCBI:txid4565; AABBDD) variety was used because it is a commonly used experimental line with a sequenced genome and extensive functional genetic resources. The diploid progenitor species *Aegilops tauschii* (NCBI:txid200361; DD), accession AL8/78, that has a sequenced genome was used for most experiments. Two divergent *Ae tauschii* lines, Clae23 and ENT336, were also used for comparative transcriptomics. All plants were grown in a glasshouse with supplementary lighting (12–24°C, 16/8 h light/dark cycle).

### Chromosome 3DL arm assembly and annotation

A set of bacterial artificial chromosome (BAC) scaffolds of flow-sorted chromosome 3DL [17] was extended and scaffolded using wheat Pacific Biosciences assemblies from Triticum 3.1 [18] as templates ([19]; Additional File 1). These scaffolds were further extended using Fosill long mate-pair reads [17]. The chromosome 3DL pseudomolecule was assessed by mapping the resulting 504 scaffolds to International Wheat Genome Sequencing Consortium (IWGSC) 3D [20]. The scaffolds were localized and assigned to a specific order and strand and linked (Additional File 2). Order discrepancies were manually corrected and 100 Ns were placed between neighbouring scaffolds to mark the sequence gap (Supplementary Table S1). Chromosome 3DL genes were predicted by *ab initio*methods, using EST and *de novo* transcript assemblies. Predicted gene models were curated manually and given a confidence score using RNA evidence, protein alignments, and *ab initio* predictions. Pseudogenes were annotated and identified as those genes with predicted exon-intron structures conforming to the GT-AG intron rule, but with no consensus or translatable coding sequences (CDS). A total of 3,927 genes were identified on 3DL of which 3,540 were located on anchored scaffolds (Additional File 4). Seventy-five percent of the annotated genes were predicted with high confidence, and 192 pseudogenes were identified on the basis of gene models with conserved exon-intron structures that lacked an identifiable coding sequence. Approximately 70% of the assembled sequences were repetitive (Supplementary Table S2), comprising 52% class I long terminal repeat retrotransposons and 12% class II DNA transposons. Supplemental Table S3 describes the assembly of chromosome 3DL. Gene order between hexaploid wheat 3DL and *Ae tauschii* AL8/78 genome pseudomolecules was determined using gene annotations from Luo et al. [21]. An additional 1,266 additional genes were identified on chromosome 3 L using wheat 3DL gene models and *Ae tauschii* RNA-seq and transcript assemblies to assign a total of 4,121 genes to chromosome 3 L.

### RNA sequence datasets

Triplicated samples of wheat and *Ae tauschii* AL8/78 were collected from leaves and roots of 14-day-old greenhouse-grown plants, 4-day-old seedlings germinated on filter paper, and 10 days after pollination (DAP) and 27 DAP developing grains (Supplementary Table S3), frozen in liquid nitrogen and stored at −80 °C. Total RNA was extracted as described [22]. Illumina TruSeq messenger RNA libraries were constructed according to the manufacturer's protocol. All sequencing was carried out on an Illumina HiSeq 2500, with 100 bp paired-end read metric, TruSeq SBS V3 Sequencing kit, and version 1.12.4.2 RTA.

### Bisulphite sequence datasets

Triplicated DNA samples from *Ae tauschii* AL8/78 and Paragon wheat 14-day-old leaves were extracted for analysis. For wheat, whole-genome bisulphite sequencing was carried out, while gene capture with Agilent SureSelect Target Enrichment was used for *Ae tauschii* chromosome 3 L predicted genes. Bisulfite treatment of triplicated samples used the Zymo Research EZ DNA Methylation-Gold Kit, standard Illumina library preparation, and sequencing using a Hiseq 4000 (2 × 150 bp reads) (Illumina Hiseq 4000, RRID:SCR_016386) for *Ae tauschii* samples and a Hiseq 2500 (2 × 250 bp reads) (Illumina Hiseq 2500, RRID:SCR_016383) for wheat samples. Chromosome 3DL methylation status was identified by alignment of 13,046,879 bp of wheat methyl-sequences with the wheat 3DL pseudomolecule. For *Ae tauschii*, 19,519,314 bp of methyl-sequences across 4,130 sequences (18,975,440 bp of unique sequence) were identified on chromosome 3 L.

### Assay for transposase-accessible chromatin sequence datasets

Nuclei were isolated rapidly from triplicated wheat and *Ae tauschii* leaf protoplast samples and subjected to transposition using Nextera transposase (Illumina). Purified DNA was used as a control. Amplified tagmented DNA was sequenced on a HiSeq X Illumina sequencer (Illumina HiSeq X, RRID:SCR_016385) with 150 bp paired-end reads (Novagene, Shenzhen, China). Assay for transposase-accessible chromatin sequencing (ATAC-seq) reads matching mitochondrial and chloroplast genomes were identified and removed using the *T. aestivum* chloroplast genome (GenBank accession No. NC_002762), *T. aestivum* mitochondrial genome (GenBank accession No. AP008982), and the *Ae tauschii* chloroplast genome (GenBank accession No. NC_022133).

## Analyses

### Extensive conservation of genes on wheat chromosome 3DL and *Ae tauschii* 3 L

**Figure 1:**
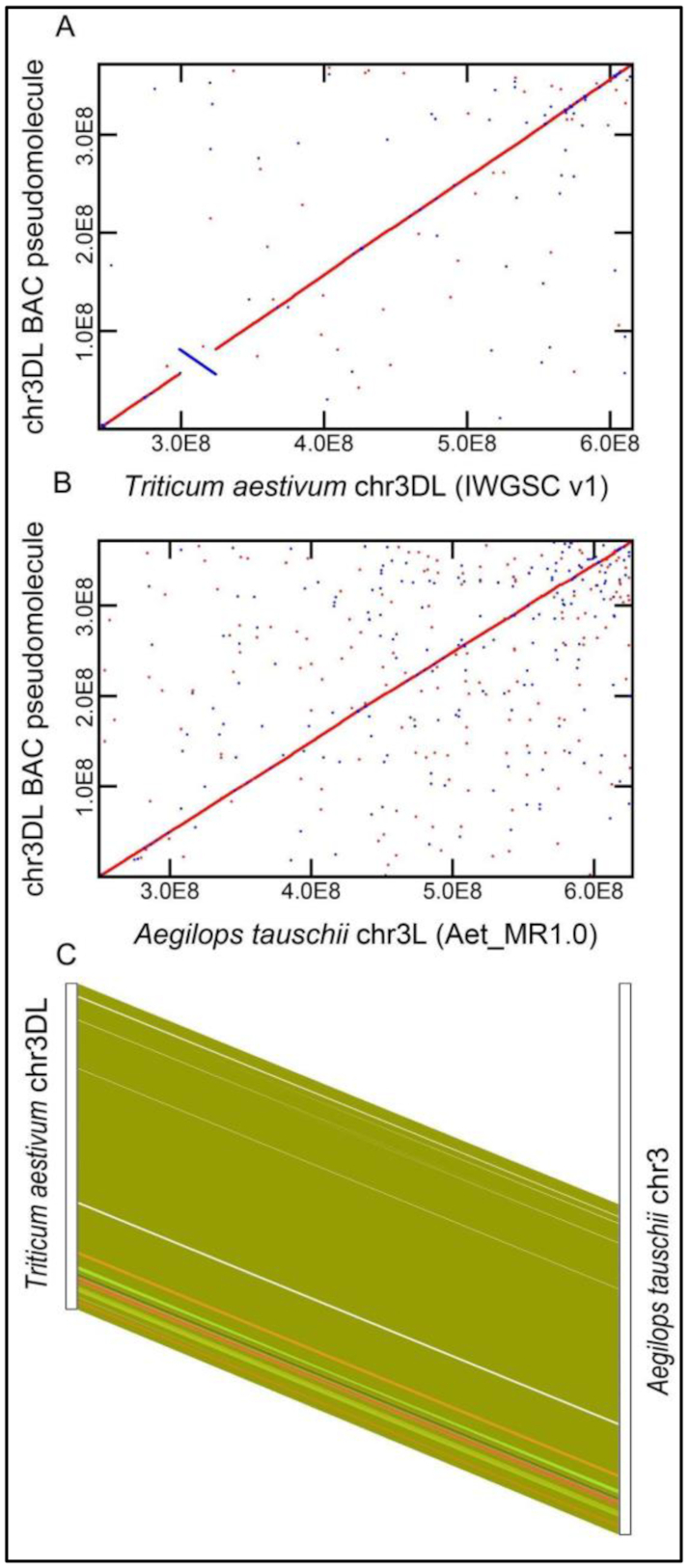
Highly conserved sequence and gene order between *Ae tauschii* 3 L and wheat 3DL chromosome arms. MUMmerplot alignments are shown of the chromosome 3DL BAC-based pseudomolecule (on the y-axis) to the v1 IWGSC assembly of Chinese Spring chromosome 3DL (**A**) and the *Ae tauschii* AL8/78 chromosome 3 L assembly. Alignments are shown by the diagonal red line. A large inversion (blue line) can be seen in the IWGSC v1 assembly of 3DL compared to the BAC-based pseudomolecule. Chromosome coordinates are in base pairs, with chromosome 3DL BAC pseudomolecule coordinates starting at a centromeric location and extending to the telomere. (**B**) Alignment of chromosome 3DL and 3 L from *Ae tauschii* shows an essentially collinear relationship between the assemblies. (**C**) Gene synteny alignments between 3,927 hexaploid wheat chromosome 3DL genes and 4,121 *Ae tauschii* 3 L genes. The coordinates of 3,456 gene pairs are shown by connecting lines between the chromosome arms. The different colours show different syntenic groups. The white region in the comparison of wheat and *Ae tauschii* is due to a gene-free region. The telomeric region is at the bottom of the figure.

Accurate long-range assemblies and consistent annotations are essential prerequisites for comparing chromosome-scale features. We focused on group 3 chromosomes of pooid grasses becase they have extensive conserved gene order and clearly defined orthologous relationships [16, 23]. We prepared a BAC-based long-range assembly of wheat chromosome 3DL and compared this to a long-range assembly of chromosome 3 L of *Ae tauschii* [21]. Comparison of the 504 3DL scaffolds with the recently available IWGSC v1.0 assembly of chromosome 3D [20] corrected 13 orientation discrepancies in the IWGSC v1 assembly, including a 25.37-Mb segment (Fig. 1A), and its collinear relationship to chromosome 3 L of *Ae tauschii* (Fig. 1B). Extensive conserved syntenic gene order between wheat 3DL and *Ae tauschii* 3 L was seen (Fig. 1C), in which 3,456 of the 3,927 predicted 3DL wheat genes align to 3 L genes with a mean of 99.66% sequence identity across their full CDS (Additional File 4). Most of the 207 non-syntenic genes on wheat 3DL were located towards the telomeric ends of the chromosome arms.

### Gene expression patterns in diploid 3 L and hexaploid 3DL

**Figure 2:**
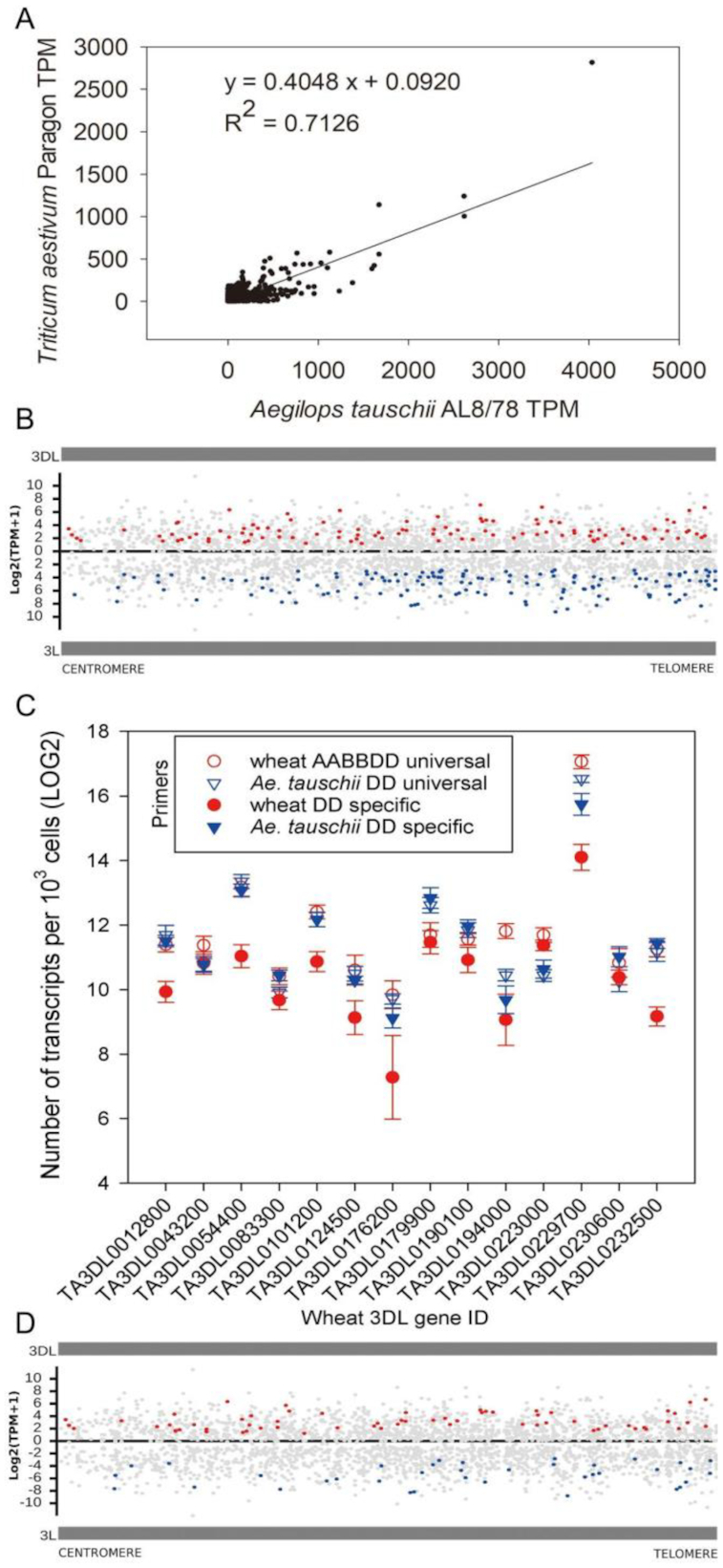
Chromosome-scale differences in expression of syntenic gene pairs in wheat 3DL and *Ae tauschii* 3 L. **(A)** Comparison of gene expression levels between syntenic pairs of hexaploid wheat 3DL and diploid AL8/78 3 L genes. In total, 2,378 (68%) of the syntenic genes were expressed in each of the 5 tissues examined. A trend of reduction of hexaploid wheat 3DL gene expression to 40% of that observed in diploid *Ae tauschii* was observed. (**B**) Chromosomal locations of differentially expressed and proportionately reduced genes in leaf tissue of wheat 3DL and *Ae tauschii* AL8/78. The locations of 3,456 syntenic gene pairs are shown on the horizontal axis, with the centromere on the left and telomere on the right. Gene expression values (TPM) are on the vertical axis, with wheat TPM values shown on the upper panel and *Ae tauschii* TPM values shown on the lower panel. Expression of DEGs is shown by blue lines. Gene expression of proportionately reduced genes is shown as gray lines. (**C**) Expression levels of 14 syntenic gene pairs on wheat 3DL and *Ae tauschii* 3 L using absolute quantitative RT-PCR. The graph shows transcript levels expressed per leaf mesophyll protoplast on the vertical axis. The 14 genes (identified on the horizontal axis) were from among non-differential genes. For 11 of the 14 gene pairs, lower expression levels (41.78% on average) of the DD gene from wheat were seen compared to the same gene in diploid *Ae tauschii*. Universal primers can amplify all the homoeologs while specific primers can only amplify DD genes. (**D**) Chromosomal locations of 106 conserved DEGs and balanced genes in leaf tissue of Paragon 3DL and 3 *Ae tauschii* accessions AL8/78, Clae23, and ENT336. As in panel **B**, red lines mark those DEGs expressed more highly in wheat than the 3 *Ae tauschii* varieties, and blue lines indicate DEGs with higher expression in the 3 *Ae tauschii* lines compared to wheat. Gray lines indicate proportionately reduced gene expression patterns.

Supplementary Fig. S1 shows expression profiles of 3DL and 3 L genes in 2-week-old greenhouse Paragon wheat and *Ae tauschii* AL8/78 plants, from 4-day-old seedlings, and from 10 and 27 DAP developing grain sampled tissues (Supplementary Table S3). In total, 2,375 (68.72%) of the syntenic genes from 3DL (1,893 genes) and/or 3 L (2,217 genes) were expressed at transcripts per million (TPM) ≥ 1 in these tissues (Additional File 4). Comparison of TPM values of syntenic gene pairs between hexaploid 3DL and diploid 3 L (Fig. 2A) showed a significant trend in reduction of TPM values in the hexaploid context to ∼40% of those measured in diploid genomic context for ∼70% of the pairs of genes. This pattern of gene expression difference, which we called proportionately reduced expression, is shown as grey bars in Fig. 2B that mapped across the chromosome arms of wheat 3DL and *Ae tauschii* 3 L. To validate the use of TPM for comparing gene expression between hexaploid wheat and diploid *Ae tauschii*, we measured the expression of a set of 14 genes present as single copies in the wheat AA, BB, DD, and *Ae tauschii* DD genomes (Additional File 5) using RNA extracted from 50,000 leaf protoplasts and quantitative RT-PCR using a standard curve of different numbers of a plasmid molecule. For 11 of the 14 gene sets, absolute transcript levels showed the same pattern of reduction in expression in the 3DL hexaploid context to ∼40% of that measured in the D genome diploid context (Fig. 2C). The sum of AA, BB, and DD expression values was 1.2 times the diploid value on average (Fig. 2C), verified by our quantitative PCR results. This consistency validated the use of TPM values for comparing gene expression values in diploid and hexaploid genome contexts.

### Differentially expressed genes

Differentially expressed genes (DEGs) were categorized by median-normalizing wheat TPM values to *Ae tauschii* TPM values by multiplying them by 1.7604 to take account of the 70% of genes that had reduced expression levels in 3DL compared to 3 L (Fig. 2A). A threshold value of ≥4-fold change (either up in wheat or up in *Ae tauschii*) was then established as a conservative measure, compared to 30% variation used in whole-genome analyses [24]. This defined a class of 674 DEGs (28.38% out of the 2,375 expressed genes) in the 5 examined tissues (Supplementary Table S4). Fig. 2B describes these genes, which are represented as red bars for those with ≥4-fold increase in wheat or blue bars for ≥4-fold increase in *Ae tauschii* on a chromosome map. It is possible that these differences in expression were due to differences between the sequenced *Ae tauschii* variety AL8/78 and the donor of the D genome to bread wheat. We therefore carried out RNA-seq analyses of 2 other diverse *Ae tauschii* varieties, Clae23 and ENT336. Leaf RNA-seq data from Clae23 and ENT336 were mapped to 3 L (Supplementary Fig. S2). Of the 262 (17% of 1,564 expressed genes) leaf-specific DEGs identified in wheat 3DL and *Ae tauschii* AL8/8 3 L, 106 were conserved in all 3 *Ae tauschii* varieties. Mapping of these conserved DEGs to 3DL and 3 L showed that they occurred along the entire chromosome arm, with no evidence for regional differences (Fig. 2D).

**Figure 3:**
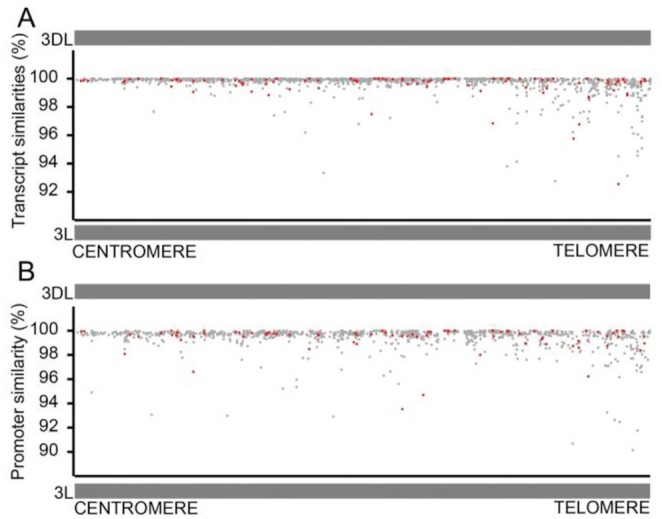
Sequence differences of transcripts and promoters of syntenic gene pairs are not related to patterns of gene expression differences in hexaploid wheat and 3 diploid *Ae tauschii* lines. (**A**) Sequence differences between gene transcripts of 106 syntenic genes with conserved differential gene expression patterns on the long arms of wheat 3DL and 3 L of *Ae tauschii*. The vertical axis shows percent similarities in predicted transcript sequences, and on the x-axis the telomeric end of the chromosome arm is on the right. Red dots indicate the 106 consensus DEGs among 3 *Ae tauschii* accessions, and the grey dots identify genes with proportionately reduced expression. Increased transcript sequence divergence is seen towards the telomere (Spearman correlation test −0.6913737 in 8-Mb bins), while DEGs and genes with proportionately reduced expression were distributed along the chromosome arm. **(B)** Sequence differences between promoters of 106 syntenic genes with conserved differential gene expression patterns on the long arms of wheat 3DL and 3 L of *Ae tauschii*. The vertical axis shows percent similarities in predicted promoter sequences, defined as 2 kb upstream of the predicted transcription start site. Red dots indicate the 106 consensus DEGs among 3 *Ae tauschii* accessions, and the grey dots identify genes with proportionately reduced expression. Promoter sequence divergence was also more pronounced towards the telomeres (Spearman correlation test −0.398358), but there was no clear relationship between promoter divergence and differential gene expression.

Most gene sequences (CDS and untranslated region [UTR]) had >99% sequence similarity between wheat 3DL and *Ae tauschii* 3 L, with a pattern of greater diversity towards chromosome ends (Fig. 3A). Promoter sequence variation was also more pronounced towards chromosome ends (Fig. 3B). However, there was no clear relationship between promoter and gene sequence divergence and differential patterns of gene expression. In addition, only 4 of the 106 leaf DEGs showed minor differences in exon-intron structure (Supplementary Fig. S3).

We identified 86 DEGs on 3DL that were up-regulated in 27 DAP developing grain of wheat compared to *Ae tauschii* (Additional File 6). Sixty-three were classified using GO terms as involved in protein targeting and degradation, RNA transcription, processing, and translation regulation. Several promoter motifs were enriched in these genes, suggesting that the DD genome contributed genes with new roles in the later stages of grain development. Thirty-three of these 86 DEGS had previously defined tissue-specific gene expression patterns [25] with 27 most highly expressed in 20 DAP aleurone layer samples. These 33 genes were also expressed in 20 DAP whole endosperm, 20 DAP transfer cell, and 20 and 27 DAP starchy endosperm tissue samples. Thus hexploidy leads to differential regulation of 3 L genes during later stages of grain development in wheat.

### Comparative DNA methylation

**Figure 4:**
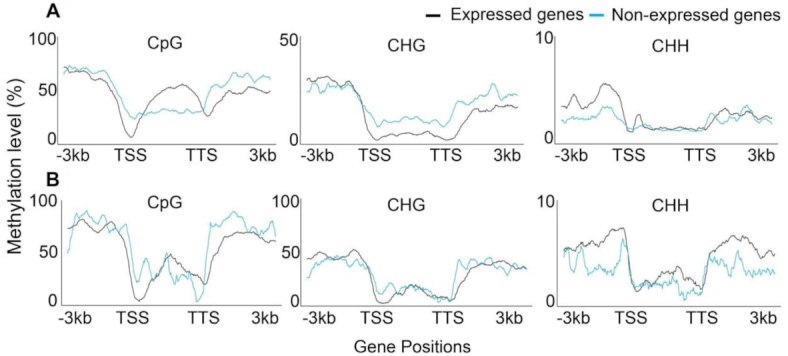
Mean methylation across all expressed and non-expressed genes on hexaploid wheat 3DL and diploid *Ae tauschii* 3 L chromosome arms. (**A**) The distribution of CpG, CHG, and CHH DNA methylation contexts across expressed and non-expressed genes on wheat chromosome 3DL. (**B**) The distribution of CpG, CHG, and CHH DNA methylation contexts across expressed and non-expressed genes on *Ae tauschii* AL8/78 chromosome 3 L. TSS: transcriptional start site; TTS: transcriptional termination site. Methylation was assessed 3 kb upstream and downstream of the TSS.

The role of gene body and promoter methylation in the altered patterns of gene expression observed in chromosomes 3 L and 3DL was determined using exome capture and bisulphite sequencing of *Ae tauschii* AL8/78, and whole-genome bisulphite sequencing of hexaploid wheat. Reads were mapped to each complete genome, those mapping to chromosomes 3 L and 3DL were identified, and their methylation in CpG, CHG, or CHH contexts characterized. Overall levels of 3 L and 3DL methylation were similar, with 89.9%, 59.4%, and 3.8% methylation of CpG, CHG, and CHH sites for wheat and 87.1%, 53.4%, and 3.4% for *Ae tauschii* AL8/78 (Supplementary Table S5). Average methylation levels were assessed across normalized promoter and gene body lengths of expressed and non-expressed genes for CpG, CHG, and CHH contexts to define relationships between gene methylation and gene expression. Fig. 4 shows a decrease of CpG methylation at the transcriptional start site (TSS) and transcription termination site (TTS) compared to the promoter and gene body regions. This pattern of reduced TSS methylation of CpG and CHH contexts is more marked for expressed genes than non-expressed genes in both 3 L and 3DL. CpG and CHG methylation at the TSS (±20 bp) is significantly lower in expressed genes compared to non-expressed genes (CpG sites: *P* < 0.0001, *t* = 5.9739, df = 80; CHG sites: *P* < 0.0001, *t* = 6.3446, df = 80).

To identify differentially methylated regions (DMRs) DNA methylation at CpG, CHG, and CHH sites was averaged independently across each gene-body and promoter region. A gene/promoter region was only analysed if a minimum of 5 methylated cytosines were included in the region, each with a minimum bisulphite sequencing coverage of 5× for *Ae tauschii* 3 L and 10× for Paragon 3DL, because it had a higher sequence depth coverage. This identified 2,709 unique gene-regions (81.9% of the total genes) and 2,182 unique promoter regions across the CpG/CHG/CHH contexts for chromosome 3DL and 2,952 unique gene-regions (71.5% of the total genes) and 2,719 unique promoter regions for chromosome 3 L (Supplementary Table S6). Comparison of methylation between Paragon and *Ae tauschii* for 2,224 gene pairs in total (64.35%) across CpG/CHG/CHH gene and promoter sites (1,920 genes and 1,368 promoters) identified DMRs. DMRs were defined if a CpG region showed a difference in methylation of ≥50% (*q* < 0.05), a CHG region showed a difference of ≥25%, or a CHH site showed a difference of ≥10%. Only 11.3% of differentially methylated genes or promoters were correlated with differences in gene expression between Paragon 3DL and *Ae tauschii* 3 L (Table 1). This showed that differential promoter and gene methylation may account for only a small proportion of observed gene expression differences between diploid 3 L and hexaploid 3DL genes.

**Table 1:** Differentially methylated genes that are also differentially expressed between wheat 3DL genes and *Ae tauschii* 3 L genes

Parameter	CpG	CHG	CHH
Genes showing differential methylation (*q* < 0.05) (% total genes)	526 (15%)	169 (5%)	42 (1%)
Genes showing differential methylation and DEGs	64 (12%)	18 (11%)	5 (12%)
Promoters showing differential methylation (*q* < 0.05) (% total genes)	263 (8%)	85 (3%)	123 (4%)
Promoters showing differential methylation and expression 4-fold	27 (10%)	6 (7%)	17 (13%)

### Pseudogene analyses

Pseudogene formation as a consequence of polyploidization has been proposed to be an important driver of genomic change [26]. A total of 192 pseudogenes were defined on chromosome 3DL on the basis of well-defined exon-intron structures using EST, protein, and RNA-seq evidence, but without a predicted CDS region. Among the 160 pseudogenes that were syntenic on 3DL and 3 L, 66 (43 in Paragon and 49 in AL8/78) were expressed with ≥1 TPM in the examined tissues. DNA methylation analyses of the 160 syntenic pseudogenes revealed CpG, CHG, or CHH gene methylation data for 85, 82, and 101 genes and 45, 46, and 67 promoters, respectively. Supplementary Table S7 compares the methylation levels of pseudogenes with the methylation patterns of all analysed genes on 3 L and 3DL. Methylation levels were generally elevated in pseudogenes in both Paragon and *Ae tauschii*, by 13.2% for CpG sites and 12.6% for CHG sites, in comparison with non-pseudogenes. Methylation levels were not increased at CHH sites in pseudogenes (mean difference −0.4%). We identified 7 pseudogenes on wheat 3DL that were intact genes in *Ae tauschii* 3 L, indicating a potential recent origin (Supplementary Table S8). Three of these *Ae tauschii* 3 L genes were expressed, but none were in wheat, confirming their identification as pseudogenes. Available methylation data for 4 of these gene pairs showed a large increase in CpG methylation in the wheat pseudogenes compared with their functional *Ae tauschii* counterparts.

### Chromatin accessibility

Differences in chromatin accessibility are key factors in chromosome-scale patterns of gene expression changes seen in dosage compensation [27] and in large-scale transcriptional reprogramming [28]. We therefore carried out ATAC-seq [29] in nuclei prepared from hexaploid wheat and diploid *Ae tauschii* AL8/78 leaf protoplasts. Supplementary Fig. S4 shows the profile of ATAC fragment sizes on chromosomes 3 L and 3DL. The ∼10.5 bp periodicity reflects cleavage of the DNA helix, and a trace of single and double nucleosome spacing can be seen in the 3 L ATAC fragment length frequency plot. In both *Ae tauschii* and wheat, normalized density plots of ATAC peaks showed that these were mainly found in 5′UTR + promoter regions (Supplementary Fig. S5), consistent with more accessible chromatin in putative transcriptional regulatory regions observed in mammalian cells [29]. Peak lengths showed that regions of accessible chromatin extended to >5 nucleosomes, with a peak at 1–2 nucleosome spacing. In *Ae tauschii* leaf nuclei 4,960 ATAC peaks on 936 genes were identified on diploid 3 L and 2,970 ATAC peaks in 1,187 genes were identified on hexaploid 3DL leaf nuclei (Supplementary Table S9). Interestingly, 26.53% of wheat and 28.64% of *Ae tauschii* ATAC peaks mapped to intergenic regions. Approximately 60% of these intergenic ATAC peaks were in regions that were not annotated as repeats, while nearly 75% of the ATAC peaks that mapped to annotated repeats were found over simple sequence repeats (SSRs) (Supplementary Fig. S6A). Only a small proportion of the annotated repeats on 3DL and 3 L had accessible chromatin, the largest of which was 8% of SSR on 3 L (Supplementary Fig. S6B).

**Figure 5:**
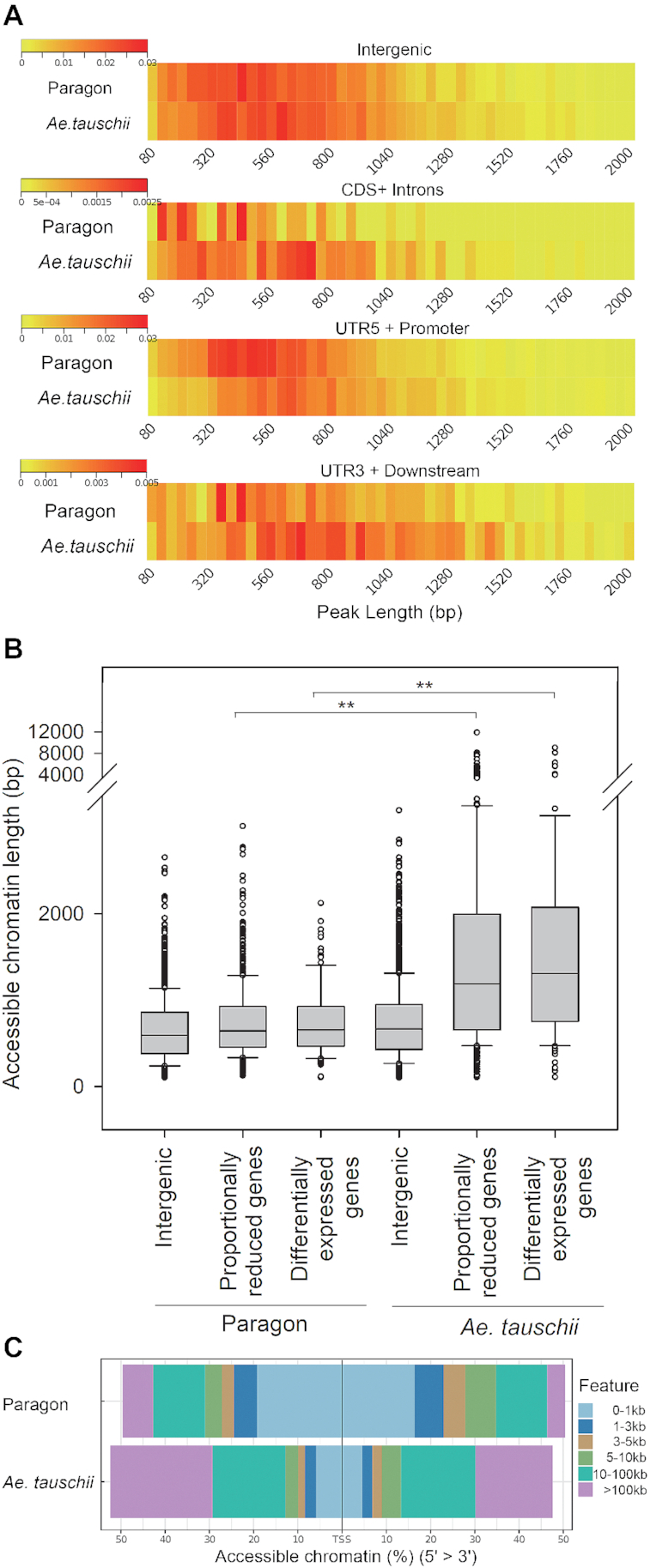
The distribution of accessible chromatin on wheat chromosome 3DL and *Ae tauschii* chromosome 3 L. (**A**) Normalized ATAC peak length enrichment for 4 classes of chromosomal regions on Paragon wheat chromosome 3DL and *Ae tauschii* 3 L. The chromosomal regions are intergenic, CDS+introns, 5′UTR+2 kb upstream putative promoter region, and 3′UTR+2 kb downstream. ATAC peak length distributions are shown in base pairs on the horizontal axis. The colour scale shows ATAC peak frequency distribution per bin. (**B**) Box plots of ATAC peak lengths across intergenic regions, genes with proportionately reduced expression patterns, and genes showing differential expression between wheat 3DL and *Ae tauschii* 3 L. The significance of ATAC peak length differences was assessed by 1-way analysis of variance. Peak length differences for balanced genes between wheat at *Ae tauschii* were significant at 6.99E−68, and 1.34E−10 for differentially expressed genes. (**C**) Normalized distance distribution of ATAC-sequence peaks relative to the TSS of genes in hexaploid wheat and diploid *Ae tauschii* genes.

**Figure 6:**
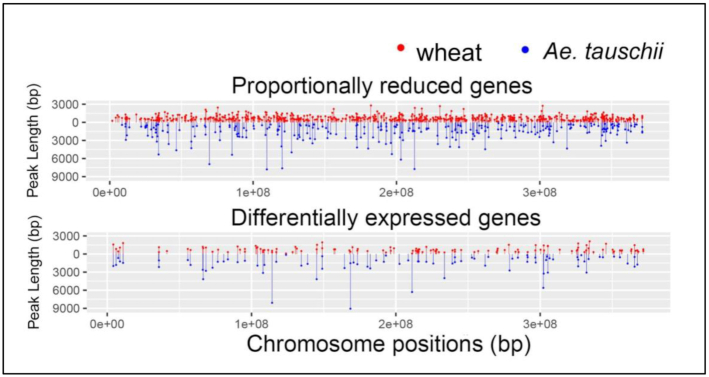
ATAC peak length distributions across syntenic genes with either proportionately reduced expression patterns (*upper panel*) or differential expression patterns (*lower panel*) across chromosome 3DL and 3 L. Red lines and dots mark wheat ATAC peak lengths on the promoter+5′UTR regions of genes, while blue lines and dots mark ATAC peak lengths on the promoter+5′UTR regions of *Ae tauschii* genes.

ATAC peak length density plots (Fig. 5A) showed an overall restriction of accessible chromatin in genic regions of hexaploid 3DL compared to diploid 3 L, with highly significant differences in genes with both proportionately reduced and differential patterns of gene expression (Fig. 5B). Typical patterns of reduced ATAC peak coverage of promoters of 2 syntenic pairs of wheat genes compared with their *Ae tauschii* counterparts involved loss of additional peaks and reduced width of a common peak and are shown in Supplementary Fig. S7. Peak length distributions mapped to TSS of hexaploid 3DL and diploid 3 L genes showed that ∼60% of accessible regions were found within 100 kb of the TSS in diploid 3 L genes. In contrast, 50% of accessible chromatin was found within 3 kb of hexaploid 3DL gene TSSs (Fig. 5C). This large-scale restriction of genic chromatin accessibility in hexaploid 3DL compared with diploid 3 L genes mapped across the entire chromosome arms (Fig. 6).

**Figure 7:**
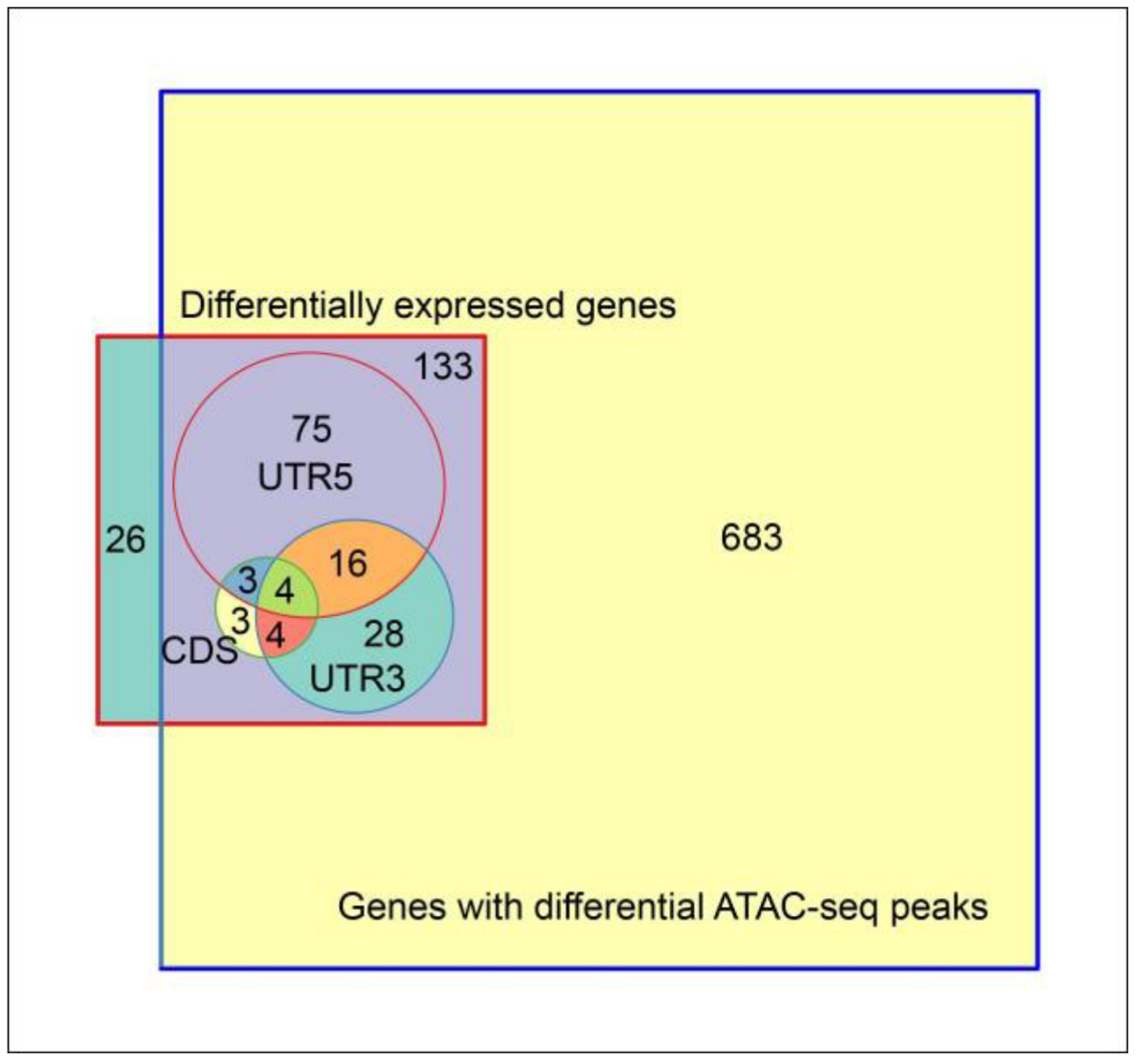
Relationships between differentially expressed genes and differential ATAC peaks. The Venn diagram shows 683 genes with differential ATAC peaks on any region of genes on hexaploid 3DL and diploid 3 L. In total, 133 of 159 genes that are differentially expressed in leaf tissue (the tissue used for ATAC-seq) have differential ATAC peaks. Of these, most differential ATAC peaks occurred in the 5′UTR+promoter region (shown as UTR5).

Of the 683 genes with differential ATAC peaks between hexploid 3DL and diploid 3 L, 133 of 159 genes (84%) that were differentially expressed in leaf tissue (the tissue used for ATAC-seq) also had differential ATAC peaks (Fig. 7; Supplementary Table S10). Of these, most different ATAC peaks occurred in the 5′UTR+promoter regions. These analyses show that the majority of DEGs in hexaploid 3DL had reduced chromatin accessibility mainly over their 5′ UTR5+promoter regions. These patterns indicate that chromatin accessibility influenced differential gene expression.

## Discussion

We used long-range assemblies and detailed annotations of homologous chromosome arms of diploid *Ae tauschii* (3 L) and hexaploid wheat (3DL) to characterize the expression of precisely defined syntenic gene pairs from these chromosome arms in diploid and hexaploid genome contexts. Approximately 70% of expressed gene pairs showed a proportionate reduction of gene expression in the hexaploid chromosome arm to 40% of that in the diploid chromosome. This pattern of reduced expression probably reflects balancing gene expression, in which ∼70% of 1:1:1 AABBDD homoeologs were shown to be expressed at an approximate mid-point level [24]. Similar overall reductions in expression have been observed in newly formed wheat hybrids [14, 30, 31], suggesting that a global re-alignment of gene expression to near-diploid levels is an early consequence of hexaploidy. The remaining 30% of gene pairs in diploid 3 L and hexaploid 3DL exhibited differential expression patterns that were significantly higher or lower in the hexaploid 3DL context compared to the diploid 3 L context. This proportion is also similar to that observed in analyses of complete wheat gene sets in the hexaploid genome [24]. We were not able to identify any relationships between sequence divergence in putative promoter or gene sequences between 3 L and 3DL gene pairs that may account for these differential expression patterns. A significant proportion of DEGs were expressed during the later stages of grain development, consistent with the differential contributions of the AA, BB, and DD genomes to functional modules involved in seed development identified by co-expression analyses [32]. Enrichment of promoter motifs in these 87 DEGs is consistent with a model in which transcriptional regulation from the AABB genomes may differentially regulate DD genes during grain development. Such emergent patterns of *cis-trans* interactions in polyploid genomes have been modelled as an important consequence of polyploidy [33,34].

Differences in gene methylation have been proposed to contribute to differences in gene expression between progenitor and allopolyploid species [35,36]. Only 11% of DEGs had different gene and promoter methylation patterns that might cause altered expression, indicating a relatively minor influence of methylation differences on gene expression patterns in 3DL and 3 L, which might be partly affected by gene capturing efficiency. Of the 7 pseudogenes found in 3DL that had an intact homolog in 3 L, bisulphite sequence data for 4 showed large increases in CpG methylation. This is consistent with extensive methylation of pseudogenes seen in many plant species [37,38]. Whether DNA methylation is a cause or consequence of pseudogene formation is not known in these examples.

Given the lack of evidence for differences in sequence composition (including gene loss and pseudogenization) or DNA methylation that could account for the major patterns of altered gene expression observed across chromosomes 3 L and 3DL, what mechanisms may be responsible? Changes in small RNA and chromatin, as measured by chromosome immunofluorescence [14], accompany the formation of new allotetraploid wheat lines, suggesting that some forms of chromatin modification may contribute to altered gene expression in wheat allopolyploids. Dynamic interplay between nucleosomes, transcription factors, and chromatin remodelling proteins alters physical access to DNA in chromatin [39] and provides a direct measure of chromatin states involved in gene expression, such as the occupation of promoter and enhancer sequences by transcription factors and other proteins. ATAC sequencing [29] was used to assess chromatin accessibility in leaf nuclei of hexaploid wheat and diploid *Ae tauschii*. Chromatin in promoter and 5′UTR regions of genes in the diploid context had more accessible chromatin, both in terms of peak numbers and peak lengths, than in the hexaploid context. This restriction of chromatin accessibility in the hexaploid context extended across the 3DL chromosome arm, encompassing genes with both proportionately reduced and differential expression. Proportionately reduced gene expression on 3DL was correlated with reduced chromatin accessibility compared to diploid 3 L. Differences in chromatin accessibility, as measured by ATAC, are thought to be due to passive competition for DNA between nucleosomes and transcription factors, chromatin remodelling and architectural proteins [39]. It is possible that in the hexaploid context reduced chromatin access across genic regions of 3DL may be due to altered competition for DNA between increased nucleosome formation or reduced transcription factor levels, or a combination of both.

The overall similarities in chromatin accessibility in intergenic regions of both 3 L and 3DL may be due to higher-order nucleosome packaging in heterochromatin, which is characteristic of intergenic DNA in grass genomes [40]. The relatively high levels of chromatin accessibility in SSRs may reflect possible altered nucleosome interactions with these atypical sequences, as Alu repeats influence nucleosome spacing in human cells [41].

The introduction of a divergent set of gene regulatory proteins from the 4-Gb *Ae tauschii* genome into a 12-Gb tetraploid nucleus may lead to altered interactions between the new set of transcription factors and nucleosomes, thus altering chromatin accessibility. Models of homoeolog expression patterns in allopolyploids that include varying affinities and concentrations of transcription factors for gene regulatory sequences in an allopolyploid showed a strong effect of inter-genome interactions on altered gene expression [34]. Recently, the conformation of cotton chromosomes was shown to be strongly affected by polyploidy, which altered topologically associating domain (TAD) boundaries and chromatin states [42]. Similar changes may occur in wheat upon polyploidization.

## Potential Implications

We have identified chromosome-wide changes in chromatin accessibility in a pair of homologous Triticeae chromosome arms in diploid and hexaploid genome contexts that may establish and maintain the large-scale differences in gene expression observed upon formation of polyploid genomes. A wide range of chromatin analysis methods are currently available for studying genome-scale changes in chromatin in newly formed polyploids to further explore mechanisms that impart new patterns of gene expression in polyploid genomes. These analyses will be able to establish comprehensive mechanisms that explain the rapid emergence and stable maintenance of new traits in polyploid crop plants such as bread wheat.

## Methods

### Gene synteny

*Ae tauschii* AL8/78 genome pseudomolecules and their gene annotations were from [21]. A total of 1,266 additional genes were identified on chromosome 3 L using wheat 3DL gene models and *Ae tauschii* RNA-seq and transcript assemblies to assign a total of 4,121 genes to chromosome 3 L. Similarity searches between wheat and several other sequenced grass species were performed using BLAST+ (v2.6.0; parameter: -evalue 1e-10 -outfmt 6 -num_alignments 5) [43], and gene synteny and collinearity were detected using MCScanX with default settings [44], and plotted using VGSC (v1.1) [45].

### Transcription analyses

Reads were mapped to the complete Triticum3.1 genome assembly with 3DL assemblies replaced by our chromosome 3DL pseudomolecule. Expression differences between wheat and *Ae tauschii* used the HISAT2-StringTie pipeline [46] to compute TPM values (described online at [47]). Absolute quantitative RT-PCR was used to validate TPM value comparisons between the diploid and hexaploid chromosome arms. Fourteen non-DEG genes were identified and 2 pairs of primers for RT-PCR were designed by the HomoeologPrimer (HomoeologPrimer, RRID:SCR_017559) (Additional File 5). One pair was designed to amplify all 3 gene copies in hexaploid wheat and the single copy in diploid *Ae tauschii*, and a second pair was designed to specifically amplify only the DD genome copy. Triplicate samples of 50,000 leaf protoplasts from wheat and *Ae tauschii* AL8/78 from young leaves were collected and RNA extracted. Quantitative PCR was performed on complementary DNA using a LightCycler 480 System (Roche), and a standard curve of 8–80 m molecules of the 5,340-bp plasmid pETnT was used to estimate absolute transcript levels.

### Bisulphite sequencing

A full description is provided on protocols.io [48]. Bisulfite converted wheat paired-end sequences were aligned to wheat genome assemblies using Bismark (version 0.18.1) [49]. The methylation status of each cytosine residue across the sequences was identified using the Bismark methylation extractor tool and the percentage of reads methylated per cytosine residue across the wheat 3DL and *Ae tauschii* sequences was calculated.

### ATAC-seq sequencing

A protocol adapted for wheat and *Ae tauschii* leaf nuclei is described (see details at [50]) and was used with the ATACseqMappingPipeline (ATACseqMappingPipeline, RRID:SCR_017558). Duplicate reads were removed using the Picard tools MarkDuplicates program [49]. All reads aligning to the forward strand were offset by +4 bp, and all reads aligning to the reverse complement strand were offset by −5 bp [51]. ATAC-Seq peak regions of each sample were called using MACS2 (v2.1.2_dev) [52] and only peak areas generated in all 3 independent experiments were used in subsequent analyses. ATAC-Seq peaks for which the distance between proximal ends was <10 bp were merged. Four classes of peak areas were assessed for chromatin accessibility: 5′UTR + promoter (including from the ATG to 2 kb upstream), CDS (the gene coding region and predicted intron sequences), 3′UTR + downstream (including the 3′ UTR to 2 kb downstream), and intergenic (regions > 2 kb distance from genes).

## Availability of Supporting Data and Materials

The chr3DL assembly and annotation data generated in this study have been submitted to the EBI European Nucleotide Archive (ENA) database (https://www.ebi.ac.uk/ena) under accession number PRJEB23358. Paragon RNA-seq data are under PRJEB29855; *Ae tauschii* AL8/78 RNA-seq data are under PRJEB23317 as described in the *Ae tauschii* genome article [21]. *Ae tauschii* Clae 23 leaf RNA-seq data are under PRJEB29859; *Ae tauschii* ENT336 leaf RNA-seq data are under PRJEB29860; Paragon ATAC-seq data are under PRJEB29868; AL8/78 ATAC-seq data are under PRJEB29869. Gene methylation data for *Ae tauschii* AL8/78 3 L and Paragon wheat 3DL data are under PRJEB31186. All supporting data and materials are available in the *GigaScience* database GigaDB [53].

## Additional Files

Additional File 1. Supplementary Methods

Additional File 2. Chromosome Arm Sequence Assembly

Additional File 3. Supplementary Figures and Tables

Supplementary Figure S1. Expression profiles of syntenic genes of Triticum aestivum Paragon 3DL and Aegilops tauschii AL8/78 3L in five tissues

Supplementary Figure S2. Comparison of expression levels of between Aegilops tauschii varieties AL8/78, Clae23 and ENT336

Supplementary Figure S3. Illustration of gene structure differences among 106 conserved DEGs between wheat and Ae. tauschii

Supplementary Figure S4. Size distribution of ATAC-seq fragment lengths between Triticum aestivum Paragon and Aegilops tauschii AL8/78

Supplementary Figure S5. Normalised read enrichment for four classes of chromosome states in Triticum aestivum Paragon and Aegilops tauschii AL8/78

Supplementary Figure S6. Distribution of chromatin accessibility across intergenic regions of hexaploid wheat 3DL and Ae. tauschii 3L chromosome arms

Supplementary Figure S7. ATAC peaks on pairs of syntenic genes in hexaploid wheat and diploid Ae tauschii". "Table S1 "Sequence assembly of wheat chromosome 3DL Scaffolds

Supplementary Table S1. Sequence assembly of wheat chromosome 3DL Scaffolds

Supplementary Table S2. Summary of repetitive elements of Triticum aestivum Chinese Spring 3DL and Aegilops tauschii AL8/78 3L

Supplementary Table S3. Illustration of gene structure differences among 106 conserved DEGs between wheat and Ae. tauschii

Supplementary Table S4. Number of differential expressed genes in 5 tissues between Triticum aestivum Paragon and Aegilops tauschii AL8/78

Supplementary Table S5. Mapping statistics after alignment of bisulfite treated Triticum aestivum Paragon and Ae. tauschii AL8/78 samples

Supplementary Table S6. Number of regions for methylation analysis in Triticum aestivum Paragon 3DL and Aegilops tauschii AL8/78 3L

Supplementary Table S7. Methylation levels of pseudogenes in Paragon wheat 3DL and Ae tauschii AL8/78 3L

Supplementary Table S8. Pseudogenes in Paragon wheat 3DL that have intact counterparts in Ae. tauschii AL8/78 3L

Supplementary Table S9. Summary of ATAC peaks and covered genes in different chromosome states of Paragon wheat and Ae. tauschii AL8/78

Supplementary Table S10. Number of syntenic genes covered by ATAC-seq peaks

Additional File 4. Gene Annotation

Additional File 5. Quantitative Gene Expression

Additional File 6. Differentially Expressed Genes in Developing Grain

giaa070_GIGA-D-19-00357_Original_Submission

giaa070_GIGA-D-19-00357_Revision_1

giaa070_Response_to_Reviewer_Comments_Original_Submission

giaa070_Reviewer_1_Report_Original_Submission Z. Jeffrey Chen -- 12/4/2019 Reviewed

giaa070_Reviewer_2_Report_Original_Submission Hua Jiang -- 12/5/2019 Reviewed

giaa070_Reviewer_2_Report_Revision_1 Hua Jiang -- 4/2/2020 Reviewed

giaa070_Supplemental_Files

## Abbreviations

ATAC: assay for transposase-accessible chromatin; BAC: bacterial artificial chromosome; BLAST: Basic Local Alignment Search Tool; bp: base pairs; CDS: coding sequence; DAP: days after pollination; DEG: differentially expressed gene; DMR: differentially methylated region; EST: expressed sequence tag; Gb: gigabase pairs; GO: gene ontology; IWGSC: International Wheat Genome Sequencing Consortium; kb: kilobase pairs; Mb: megabase pairs; NCBI: National Center for Biotechnology Information; NR: non-redundant; RNA-seq: RNA sequencing; SSR: simple sequence repeat; TAD: topologically associating domain; TE: transposable element; TPM: transcripts per million; TSS: transcriptional start site; TTS: transcription termination site; UTR: untranslated region.

## Competing Interests

The authors declare that they have no competing interests.

## Funding

This work was supported by BBSRC ERA-CAPS grants BB/N005104/1, BB/N005155/1 “INTREPID” to M.W.B. and A.H. M.W.B. was also supported by BBSRC Institute Strategic Programme Grants GRO (BB/J004588/1) and GEN (BB/P013511/1) to M.W.B.

## Authors' Contributions

F.-H.L. and M.W.B. conceived and managed the research. F.-H.L. conducted bioinformatics analyses. N.M. and F.-H.L. carried out laboratory work. L.-J.G. performed DNA methylation analyses. A.H. and M.-C.L. provided material and advice prior to publication. M.W.B., L.-J.G., and F.-H.L. wrote the manuscript with contributions from N.M.
