# Supplementary material for: Reduced chromatin accessibility underlies gene expression differences in homologous chromosome arms of diploid Aegilops tauschii and hexaploid wheat
Source: Gigascience. 2020 Jun 20;9(6):giaa070. doi: 10.1093/gigascience/giaa070 (PMC7305686; doi:10.1093/gigascience/giaa070)
Supplement: giaa070_GIGA-D-19-00357_Original_Submission [file giaa070_giga-d-19-00357_original_submission.pdf]

GigaScience

Reduced chromatin accessibility underlies gene expression differences in homologous chromosome arms of diploid Aegilops tauschii and hexaploid wheat

--Manuscript Draft--

**Manuscript Number:**	GIGA-D-19-00357
**Full Title:**	Reduced chromatin accessibility underlies gene expression differences in homologous chromosome arms of diploid Aegilops tauschii and hexaploid wheat
**Article Type:**	Research
**Funding Information:**	Biotechnology and Biological Sciences Research Council (BB/N005104/1, BB/N005155/1) — Prof Michael Webster Bevan<br>Biotechnology and Biological Sciences Research Council (BB/N005155/1) — Dr Anthony Hall
**Abstract:**	Background: Polyploidy is centrally important in the evolution and domestication of plants as it leads to major genomic changes, such as altered patterns of gene expression, which are thought to underlie the emergence of new traits. Despite the common occurrence of these globally altered patterns of gene expression in polyploids, the mechanisms involved are not well understood. Results: Using a precisely defined framework of highly conserved syntenic genes on hexaploid wheat chromosome 3DL and its progenitor 3L chromosome arm of diploid Aegilops tauschii , we show that 70% of these gene pairs exhibited proportionally reduced gene expression, in which expression in the hexaploid context of the 3DL genes was approximately 40% of the levels observed in diploid Ae. tauschii. Several genes showing elevated expression during the later stages of grain development in wheat compared to Ae. tauschii . Gene sequence and methylation differences probably accounted for only a few cases of differences in gene expression. In contrast, chromosome-wide patterns of reduced chromatin accessibility of genes in the hexaploid chromosome arm compared to its diploid progenitor were correlated with both reduced gene expression and the imposition of new patterns of gene expression.<br>Conclusions: Our pilot-scale analyses show that chromatin compaction orchestrates reduced gene expression levels in hexaploid chromosome arm of wheat compared to its diploid progenitor chromosome arm.<br>Keywords: Triticum aestivum; wheat; chr3DL; synteny; ATAC; gene expression in polyploids
**Corresponding Author:**	Michael Webster Bevan, PhD<br>John Innes Centre<br>Norwich, UNITED KINGDOM
**Corresponding Author Secondary Information:**	
**Corresponding Author's Institution:**	John Innes Centre
**Corresponding Author's Secondary Institution:**	
**First Author:**	Fu-Hao Lu
**First Author Secondary Information:**	
**Order of Authors:**	Fu-Hao Lu
	Neil McKenzie
	Laura-Jayne Gardiner
	Ming-Chen Luo
	Anthony Hall
	Michael Webster Bevan, PhD

Powered by Editorial Manager® and ProduXion Manager® from Aries Systems Corporation

Order of Authors Secondary Information:	
Additional Information:	
Question	Response
Are you submitting this manuscript to a special series or article collection?	No
**Experimental design and statistics**<br><br>Full details of the experimental design and statistical methods used should be given in the Methods section, as detailed in our Minimum Standards Reporting Checklist. Information essential to interpreting the data presented should be made available in the figure legends.<br><br>Have you included all the information requested in your manuscript?	Yes
**Resources**<br><br>A description of all resources used, including antibodies, cell lines, animals and software tools, with enough information to allow them to be uniquely identified, should be included in the Methods section. Authors are strongly encouraged to cite Research Resource Identifiers (RRIDs) for antibodies, model organisms and tools, where possible.<br><br>Have you included the information requested as detailed in our Minimum Standards Reporting Checklist?	Yes
**Availability of data and materials**<br><br>All datasets and code on which the conclusions of the paper rely must be either included in your submission or deposited in publicly available repositories (where available and ethically appropriate), referencing such data using a unique identifier in the references and in the "Availability of Data and Materials" section of your manuscript.	Yes

Have you have met the above requirement as detailed in our Minimum Standards Reporting Checklist?	

main manuscript Click here to download Manuscript final Final 3L-3DL Gigascience.pdf

Reduced chromatin accessibility underlies gene expression differences in homologous chromosome arms of diploid *Aegilops tauschii* and hexaploid wheat

Fu-Hao Lu[1], Neil McKenzie[1], Laura-Jayne Gardiner[2], Ming-Cheng Luo[3], Anthony Hall[2] and Michael W Bevan[1*]

[*] Correspondence: michael.bevan@jic.ac.uk

[1]Department Cell and Developmental Biology, John Innes Centre, Norwich Research Park, Norwich, NR4 7UH, UK

[2]Earlham Institute, Norwich Research Park, Norwich, NR4 7UZ, UK

[3]Department of Plant Sciences, University of California, One Shields Avenue, Davis, CA 95616, USA

Running Title: Chromatin accessibility in polyploid wheat

Keywords: wheat, polyploidy, *Aegilops tauschii*, gene expression, DNA methylation, chromatin accessibility.

Abstract

Background: Polyploidy is centrally important in the evolution and domestication of plants as it leads to major genomic changes, such as altered patterns of gene expression, which are thought to underlie the emergence of new traits. Despite the common occurrence of these globally altered patterns of gene expression in polyploids, the mechanisms involved are not well understood. **Results:** Using a precisely defined framework of highly conserved syntenic genes on hexaploid wheat chromosome 3DL and its progenitor 3L chromosome arm of diploid *Aegilops tauschii*, we show that 70% of these gene pairs exhibited proportionally reduced gene expression, in which expression in the hexaploid context of the 3DL genes was approximately 40% of the levels observed in diploid *Ae. tauschii.* Several genes showing elevated expression during the later stages of grain development in wheat compared to *Ae. tauschii*. Gene sequence and methylation differences probably accounted for only a few cases of differences in gene expression. In contrast, chromosome-wide patterns of reduced chromatin accessibility of genes in the hexaploid chromosome arm compared to its diploid progenitor were correlated with both reduced gene expression and the imposition of new patterns of gene expression. **Conclusions:** Our pilot-scale analyses show that chromatin compaction orchestrates reduced gene expression levels in hexaploid chromosome arm of wheat compared to its diploid progenitor chromosome arm.

Keywords: Triticum aestivum; wheat; chr3DL; synteny; ATAC; gene expression in polyploids

Background

Polyploidy arises from the duplication or fusion of genomes and has occurred frequently in the lineages of many organisms, from fish to flowering plants [1]. The ancestral flowering plant lineage has undergone at least two genome duplication events, with subsequent multiple genome duplication events in different lineages [2]. Radical alterations of gene expression patterns are commonly observed consequences of polyploidisation. In newly formed allotetraploids of *Arabidopsis arenosa* and *A. thaliana* approximately 5% of genes were expressed at different levels than in the parental lines [3]. In *Tragopogon* allopolyploids, approximately 76% of homoeologous genes displayed additive expression (the average of expression measured in each parental line), and approximately 20% of transcripts exhibited non-additive expression in which gene expression levels varied from the average of parents [4]. Over longer time scales, a tendency of homoeologous gene expression from one parental genome to dominate over the other has been observed in several polyploids, including cotton, Brassicas and maize [5]. The mechanisms underlying these genome-scale changes in gene expression are poorly understood. Many crop species have undergone relatively recent polyploid events prior to and during domestication, leading to new traits and improved performance [6]. Large genomic segments such as entire chromosomes from related species are also added to crop varieties to introduce new traits [7]. Understanding how these large-scale genomic changes influence genome stability and gene expression, and how they give rise to improved performance in crops is therefore centrally important from both practical and research perspectives. Several hypotheses have been proposed to explain genomic interactions in hybrids and polyploids, ranging from complementation of differing alleles, misregulation of gene expression, epigenetic changes, and the activities of transposable elements (TEs) [8–10].

The wheat group of the Triticeae is characterised by stable tetraploid and hexaploid species that exhibit greater diversity, adaptability and potential for domestication than their diploid progenitors.

Multiple types of genomic changes, including altered expression patterns of genes and TEs, and epigenetic changes, have been proposed to contribute to this “genomic plasticity” [11]. The hexaploid bread wheat genome (*Triticum aestivum*) arose from the very recent integration of the diploid *Aegilops tauschii* DD genome into a tetraploid *T. turgidum* AABB genome [12]. The three component genomes are very closely related, sharing common ancestry in the Triticeae lineage approximately 6.5 mya (million years ago). In newly synthesised allohexaploid wheat [13] and tetraploid AADD and S’S’AA (where S’S’ is *Ae. longissima*) [14] wheat lines between 60-80% of genes were additively expressed, suggesting a dynamic re-adjustment of gene expression patterns as a consequence of allopolyploidy. Rapid asymmetric changes in short RNA, histone methylation and gene expression in the two allotetraploids are thought to contribute to genome-biased gene expression and the activation of TE transcription [14]. RNAseq analyses of newly formed allotriploid ABD and stable allohexaploid AABBDD lines generated from *T. turgidum* and *Ae. tauschii* showed rapid and extensive changes in gene expression in triploid tissues that were partly restored upon genome duplication [15]. The overall very high conservation of sequences and gene order of D genome chromosomes in diploid *Ae.tauschii* and hexaploid wheat [16] provides an important opportunity to assess differences between diploid and hexaploid states of very similar chromosomes. Here we analyse gene expression, DNA methylation and chromatin accessibility of a set of well-defined syntenic genes from diploid chromosome 3L of *Ae. tauschii* and hexaploid chromosome 3DL of bread wheat and show that reduced chromatin accessibility underlays large-scale changes in gene expression between diploid and hexaploid states.

Data Description

Plant Materials

The Paragon elite wheat (*Triticum aestivum*, AABBDD) variety was used as it is a commonly used experimental line with a sequenced genome and extensive functional genetic resources. The

diploid progenitor species *Aegilops tauschii* (DD), accession AL8/78 has a sequenced genome was used for most experiments. Two divergent *Ae. tauschii* lines, Clae23 and ENT336 were also used for comparative transcriptomics. All plants were grown in a glasshouse with supplementary lighting (12-24 ℃, 16/8h light/day).

Chromosome 3DL Arm Assembly and Annotation

A set of BAC scaffolds of flow-sorted chromosome 3DL [17] was extended and scaffolded using wheat PacBio assemblies from Triticum 3.1 [18] as templates (protocols.io at [19]; Additional File 1). These scaffolds were further extended using Fosill long mate-pair reads [17]. The chromosome 3DL pseudomolecule was assessed by mapping the resulting 504 scaffolds to IWGSC 3D [20]. The scaffolds were localised and assigned to a specific order and strand and linked (Additional File 2). Order discrepancies were manually corrected and one hundred Ns were placed between neighbouring scaffolds to mark the sequence gap (Additional File 3: Table S1). Chromosome 3DL genes were predicted by *ab initio methods*, using EST and *de novo* transcript assemblies. Predicted gene models were curated manually and given a confidence score using RNA evidence, protein alignments and *ab initio* predictions. Pseudogenes were annotated and identified as those genes with predicted exon-intron structures conforming to the GT-AG intron rule, but with no consensus or translatable coding sequences (CDS). A total of 3,927 genes were identified on 3DL of which 3,540 were located on anchored scaffolds (Additional File 4). Seventy-five percent of the annotated genes were predicted with high confidence, and 192 pseudogenes were identified based on gene models with conserved exon-intron structures that lacked an identifiable coding sequence. Approximately 70% of the assembled sequences were repetitive (Additional File 3: Table S2), comprising 52% class I LTR retrotransposons and 12% class II DNA transposons. Supplemental Table S3 describes the assembly of chromosome 3DL Gene order between hexaploid wheat 3DL and *Ae. tauschii* AL8/78 genome pseudomolecules was determined using gene annotations from [21]. An additional 1,266 additional genes were identified

on chromosome 3L using wheat 3DL gene models and *Ae. tauschii* RNAseq and transcript assemblies to assign a total of 4,121 genes to chromosome 3L.

RNA Sequence Datasets

Triplicated samples of wheat and *Ae. tauschii* AL8/78 were collected from leaves and roots of 14 day old greenhouse grown plants, 4 day old seedlings germinated on filter paper, and 10 days after pollination (DAP) and 27 DAP developing grains (Additional File 3: Table S3), frozen in liquid nitrogen and stored at -80℃. Total RNA was extracted as described [22]. Illumina TruSeq mRNA libraries were constructed according to the manufacturer's protocol. All sequencing was carried out on an Illumina HiSeq 2500, with 100 bp paired-end read metric, TruSeq SBS V3 Sequencing kit and version 1.12.4.2 RTA.

Bisulphite Sequence Datasets

Triplicated DNA samples from *Ae. tauschii* AL8/78 and Paragon wheat 14 day old leaves were extracted for analysis. For wheat, whole genome bisulphite sequencing was carried out, while gene capture with Agilent SureSelect Target Enrichment was used for *Ae. tauschii* chromosome 3L predicted genes. Bisulfite treatment of triplicated samples used the Zymo Research EZ DNA Methylation-Gold Kit, standard Illumina library preparation and sequencing using a Hiseq 4000 (2 x 150 bp reads) for *Ae. tauschii* samples and a Hiseq 2500 (2 x 250 bp reads) for wheat samples. Chromosome 3DL methylation status was identified by alignment of 13,046,879 bp of wheat methyl-sequences with the wheat 3DL pseudomolecule. For *Ae. tauschii*, 19,519,314 bp of methyl-sequences across 4,130 sequences (18,975,440 bp of unique sequence) were identified on chromosome 3L.

Assay for Transposase-Accessible Chromatin (ATAC) Sequence Datasets

Nuclei were isolated rapidly from triplicated wheat and *Ae. tauschii* leaf protoplast samples and

subjected to transposition using Nextera transposase (Illumina). Purified DNA was used as a control. Amplified tagmented DNA was sequenced on a HiSeqX Illumina sequencer with 150 bp paired-end reads (Novagene, Shenzhen). ATAC-seq reads matching mitochondrial and chloroplast genomes were identified and removed using the *Triticum aestivum* chloroplast genome (GenBank accession No. NC_002762), *Triticum aestivum* mitochondrial genome (GenBank accession No. AP008982) and the *Ae. tauschii* chloroplast genome (GenBank accession No. NC_022133).

Analyses

Extensive conservation of genes on wheat chromosome 3DL and *Ae. tauschii* 3L

Accurate long-range assemblies and consistent annotations are essential prerequisites for comparing chromosome-scale features. We focused on group 3 chromosomes of pooid grasses as they have extensive conserved gene order and clearly defined orthologous relationships [16,23]. We prepared a BAC-based long-range assembly of wheat chromosome 3DL and compared this to a long-range assembly of chromosome 3L of *Ae. tauschii* [21]. Comparison of the 504 3DL scaffolds with the recently available IWGSC v1.0 assembly of chromosome 3D [20] corrected 13 orientation discrepancies in the IWGSC v1 assembly, including a 25.37Mb segment (Fig. 1A). and its collinear relationship to chromosome 3L of *Ae. tauschii* (Fig. 1B). Extensive conserved syntenic gene order between wheat 3DL and *Ae. tauschii* 3L was seen (Fig. 1C), in which 3,456 of the 3,927 predicted 3DL wheat genes align to 3L genes with an average of 99.66% sequence identity across their full CDS (Additional File 4). Most of the 207 non-syntenic genes on wheat 3DL were located towards the telomeric ends of the chromosome arms.

Figure 1: Highly conserved sequence and gene order between *Ae. tauschii* 3L and wheat 3DL chromosome arms. MUMmerplot alignments are shown of the chromosome 3DL BAC-

based pseudomolecule (on the y axis) to the v1 IWGSC assembly of Chinese Spring chromosome 3DL (**A**) and the *Ae tauschii* AL8/78 chromosome 3L assembly. Alignments are shown by the diagonal red line. The upper panel shows a large inversion (blue line) in the IWGSC v1 assembly of 3DL compared to the BAC-based pseudomolecule. Chromosome coordinates are in base-pairs, with chromosome 3DL BAC pseudomolecule coordinates starting at a centromeric location and extending to the telomere. (**B**). Alignment of chromosome 3DL and 3L from *Ae. tauschii* shows an essentially collinear relationship between the assemblies. (**C**) Gene synteny alignments between 3,927 hexaploid wheat chromosome 3DL genes and 4,121 *Ae. tauschii* 3L genes. The coordinates of 3,456 gene pairs are shown by connecting lines between the chromosome arms. The different colours show different syntenic groups. The white region in the comparison of wheat and *Ae. tauschii* is due to a gene-free region. The telomeric region is at the bottom of the figure.

Gene expression patterns in diploid 3L and hexaploid 3DL

Additional File 3: Fig. S1 shows expression profiles of 3DL and 3L genes in 2 week old greenhouse Paragon wheat and *Ae. tauschii* AL8/78 plants, from 4 day old seedlings, and from 10 and 27 days after pollination (DAP) developing grain sampled tissues (Additional File 3: Table S3). In total, 2,375 (68.72%) of the syntenic genes from 3DL (1,893 genes) and/or 3L (2,217 genes) were expressed at TPM≥1 in these tissues (Additional File 4). Comparison of TPM values of syntenic gene pairs between hexaploid 3DL and diploid 3L (Fig. 2A) showed a significant trend in reduction of TPM values in the hexaploid context to approximately 40% of those measured in diploid genomic context for approximately 70% of the pairs of genes. This pattern of gene expression difference, which we called proportionately reduced expression, is shown as grey bars in Fig. 2B that mapped across the chromosome arms of wheat 3DL and *Ae. tauschii* 3L. To validate the use of TPM for comparing gene expression between hexaploid wheat and diploid *Ae. tauschii*, we measured the expression of a set of 14 genes present as single copies in the wheat AA, BB, DD and *Ae. tauschii* DD genomes (Additional File 5) using RNA extracted from 50,000 leaf protoplasts and quantitative RT-PCR using a standard curve of different numbers of a plasmid

molecule. For 11 of the 14 gene sets, absolute transcript levels showed the same pattern of reduction in expression in the 3DL hexaploid context to approximately 40% of that measured in the D genome diploid context (Fig. 2C). The sum of AA, BB and DD expression values was 1.2 times the diploid value on average (Fig. 2C) verified by our quantitative PCR results. This consistency validated the use of TPM values for comparing gene expression values in diploid and hexaploid genome contexts.

Figure 2: Chromosome-scale differences in expression of syntenic gene pairs in wheat 3DL and *Ae tauschii* 3L. (A) Comparison of gene expression levels between syntenic pairs of hexaploid wheat 3DL and diploid AL8/78 3L genes. 2,378 (68%) of the syntenic genes were expressed in each of the five tissues examined. A trend of reduction of hexaploid wheat 3DL gene expression to 40% of that observed in diploid *Ae. tauschii* was observed. (**B**) Chromosomal locations of differentially expressed and proportionately reduced genes in leaf tissue of wheat 3DL and *Ae. tauschii* AL8/78. The locations of 3,456 syntenic gene pairs is shown on the horizontal axis, with the centromere on the left and telomere on the right. Gene expression values (TPM) are on the vertical axis, with wheat TPM values shown on the upper panel and *Ae. tauschii* TPM values shown on the lower panel. Expression of DEGs are shown by blue lines. Gene expression of proportionately reduced genes are shown as gray lines. (**C**) Expression levels of 14 syntenic gene pairs on wheat 3DL and *Ae. tauschii* 3L using absolute quantitative RT-PCR. The graph shows transcript levels expressed per leaf mesophyll protoplast on the vertical axis. The 14 genes (identified on the horizontal axis) were from among proportionately reduced genes. For 11 of the 14 gene pairs, lower expression levels (41.78% on average) of the DD gene from wheat were seen compared to the same gene in diploid *Ae. tauschii*. (**D**) Chromosomal locations of 106 conserved DEGs and balanced genes in leaf tissue of Paragon 3DL and three *Ae. tauschii* accessions AL8/78, Clae23 and ENT336. As described in panel **B** above, red lines mark those DEGs expressed more highly in wheat than the three *Ae. tauschii* varieties, and blue lines indicate DEGs with higher expression in the three *Ae. tauschii* lines compared to wheat. Gray lines indicate proportionately reduced gene expression patterns.

Differentially expressed genes

Differentially expressed genes (DEGs) were categorised by normalizing wheat TPM values to *Ae. tauschii* TPM values by multiplying them by 1.7604 to take account of the 70% of genes that had reduced expression levels in 3DL compared to 3L (Fig. 2A). A threshold value of ≥ 4-fold change (either up in wheat or up in *Ae. tauschii*) was then established as a conservative measure, compared to 30% variation used in whole genome analyses [24]. This defined a class of 674 DEGs (28.38% out of the 2,375 expressed genes) in the 5 examined tissues (Additional File 3: Table S4). Fig. 2B describes these genes, which are represented as red bars for those with ≥4-fold increase in wheat or blue bars for ≥4-fold increase in *Ae. tauschii* on a chromosome map. It is possible that these differences in expression were due to differences between the sequenced *Ae. tauschii* variety AL8/78 and the donor of the D genome to bread wheat. We therefore carried out RNAseq analyses of two other diverse *Ae. tauschii* varieties, Clae23 and ENT336. Leaf RNAseq data from Clae23 and ENT336 was mapped to 3L (Additional File 3: Fig. S2). Of the 262 (16% of 1,564 expressed genes) leaf-specific DEGs identified in wheat 3DL and *Ae. tauschii* AL8/8 3L, 106 were conserved in all three *Ae. tauschii* varieties. Mapping of these conserved DEGs to 3DL and 3L showed that they occurred along the entire chromosome arm, with no evidence for regional differences (Fig. 2D).

Most gene sequences (CDS and UTR) had more than 99% sequence similarity between wheat 3DL and *Ae. tauschii* 3L, with a pattern of greater diversity towards chromosome ends (Fig. 3A). Promoter sequence variation was also more pronounced towards chromosome ends (Fig. 3B). However, there was no clear relationship between promoter and gene sequence divergence and differential patterns of gene expression. For example, only 4 of the 106 leaf DEGs showed minor differences in exon-intron structure (Additional File 3,: Fig. S3).

Figure 3: Sequence differences of transcripts and promoters of syntenic gene pairs are not related to patterns of gene expression differences in hexaploid wheat and three diploid *Ae. tauschii* lines. (**A**) Sequence differences between gene transcripts of 106 syntenic genes with conserved differential gene expression patterns on the long arms of wheat 3DL and 3L of *Ae. tauschii*. The vertical axis shows % similarities in predicted transcript sequences, and on the x axis the telomeric end of the chromosome arm is on the right. Black dots indicate the 106 consensus DEGs among 3 *Ae. tauschii* accessions, and the white dots identify genes with proportionately reduced expression. Increased transcript sequence divergence is seen towards the telomere (Spearman correlation test -0.6913737), while DEGs and gene with proportionately reduced expression were distributed along the chromosome arm. **(B)** Sequence differences between promoters of 106 syntenic genes with conserved differential gene expression patterns in 8 Mb bins on the long arms of wheat 3DL and 3L of *Ae. tauschii*. The vertical axis shows % similarities in predicted promoter sequences, defined as 2kb upstream of the predicted transcription start site. Black dots indicate the 106 consensus DEGs among 3 *Ae. tauschii* accessions, and the white dots identify genes with proportionately reduced expression. Promoter sequence divergence was also more pronounced towards the telomeres (Spearman correlation test -0.398358) but there was no clear relationship between promoter divergence and differential gene expression.

We identified 86 DEGs on 3DL that were up-regulated in 27 DAP developing grain of wheat compared to *Ae. tauschii* (Additional File 6). Sixty-three were classified using GO terms as involved in protein targeting and degradation, RNA transcription, processing and translation regulation. Several promoter motifs were enriched in these genes, suggesting the DD genome contributed genes with new roles in the later stages of grain development . Thirty-three of these 86 DEGS had defined previously defined tissue-specific gene expression patterns [25] with 27 most highly expressed in 20 DAP aleurone layer samples. These 33 genes were also expressed in 20 DAP whole endosperm, 20 DAP transfer cell, and 20 and 27 DAP starchy endosperm tissue samples. Thus hexploidy leads to differential regulation of 3L genes during later stages of grain development in wheat.

Comparative DNA Methylation

The role of gene body and promoter methylation in the altered patterns of gene expression observed in chromosomes 3L and 3DL was determined using exome capture and bisulphite sequencing of *Ae. tauschii* AL8/78, and whole genome bisulphite sequencing of hexaploid wheat. Reads were mapped to each complete genome, those mapping to chromosomes 3L and 3DL were identified, and their methylation in CpG, CHG or CHH contexts characterised. Overall levels of 3L and 3DL methylation were similar, with 89.9%, 59.4% and 3.8% methylation of CpG, CHG and CHH sites for wheat and 87.1%, 53.4% and 3.4% for *Ae. tauschii* AL8/78 (Additional File 3: Table S5). Average methylation levels were assessed across normalised promoter and gene body lengths of expressed and non-expressed genes for CpG, CHG and CHH contexts to define relationships between gene methylation and gene expression. Fig. 4 shows a decrease of CpG methylation at the Transcriptional Start Site (TSS) and Transcription Termination Site (TTS) compared to the promoter and gene body regions. This pattern of reduced TSS methylation of CpG and CHH contexts is more marked for expressed genes than non-expressed genes in both 3L and 3DL. CpG and CHG methylation at the TSS (+/- 20bp) is significantly lower in expressed genes compared to non-expressed genes (CpG sites; $p < 0.0001$, $t=5.9739$, $df=80$, CHG sites; $p < 0.0001$, $t=6.3446$, $df=80$).

Figure 4: Average methylation across all expressed and non- expressed genes on hexaploid wheat 3DL and diploid *Ae tauschii* 3L chromosome arms. (**A**) The distribution of CpG, CHG and CHH DNA methylation contexts across expressed and non- expressed genes on wheat chromosome 3DL. TSS: Transcriptional Start Site; TTS: Transcriptional Termination Site. Methylation was assessed 3kb upstream of TSS and 3kb downstream of the TSS. (**B**) The distribution of CpG, CHG and CHH DNA methylation contexts across expressed and non-expressed genes on *Ae. tauschii* AL8/78 chromosome 3L. TSS: Transcriptional Start Site; TTS: Transcriptional Termination Site. Methylation was assessed 3kb upstream and downstream of the TSS.

To identify differentially methylated regions (DMRs) DNA methylation at CpG, CHG and CHH sites was averaged independently across each gene-body and promoter region. A gene/promoter region was only analysed if a minimum of 5 methylated cytosines were included in the region, each with a minimum bisulphite sequencing coverage of 5x for *Ae. tauschii* 3L and 10x for Paragon 3DL, as it had a higher sequence depth coverage. This identified 2,709 unique gene-regions (81.9% of the total genes) and 2,182 unique promoter regions across the CpG/CHG/CHH contexts for chromosome 3DL and 2,952 unique gene-regions (71.5% of the total genes) and 2,719 unique promoter regions for chromosome 3L (Additional File 3: Table S6). Comparison of methylation between Paragon and *Ae. tauschii* for 2,224 gene pairs in total (64.35%) across CpG/CHG/CHH gene and promoter sites (1,920 genes and 1,368 promoters) identified DMRs. DMRs were defined if a CpG region showed a difference in methylation of 50% or more ($q < 0.05$), a CHG region showed a difference of 25% or more or a CHH site showed a difference of 10% or more. Only 11.3 % of differentially methylated genes or promoters were associated with differences in gene expression between Paragon 3DL and *Ae. tauschii* 3L (Table 1). This showed that differential promoter and gene methylation may account for only a small proportion of observed gene expression differences between diploid 3L and hexaploid 3DL genes.

Table 1:

Differentially methylated genes that are also differentially expressed between wheat 3DL genes and *Ae tauschii* 3L genes.

	CpG	CHG	CHH
Genes showing differential methylation ($q< 0.05$)	526	169	42
(% total genes)	(15%)	(5%)	(1%)
Genes showing differential methylation & DEGs	64	18	5

	(12%)	(11%)	(12%)
Promoters showing differential methylation (q< 0.05) (% total genes)	263 (8%)	85 (2.5%)	123 (3.5%)
Promoters showing differential methylation & expression 4-fold	27 (10%)	6 (7%)	17 (13%)

Pseudogene Analyses

Pseudogene formation as a consequence of polyploidisation has been proposed to be an important driver of genomic change [26]. A total of 192 pseudogenes were defined on chromosome 3DL based on well defined exon-intron structures using EST, protein and RNAseq evidence, but without a predicted CDS region. Among the 160 pseudogenes that were syntenic on 3DL and 3L, 66 (43 in Paragon and 49 in AL8/78) were expressed with at least 1 TPM in the examined tissues. DNA methylation analyses of the 160 syntenic pseudogenes revealed CpG, CHG or CHH gene methylation data for 85, 82 and 101 genes and 45, 46 and 67 promoters respectively. Additional File 3: Table S7 compares the methylation levels of pseudogenes with the methylation patterns of all analysed genes on 3L and 3DL. Methylation levels were generally elevated in pseudogenes in both Paragon and *Ae. tauschii*, by 13.2% for CpG sites and 12.6% for CHG sites, in comparison to non-pseudogenes. Methylation levels were not increased at CHH sites in pseudogenes (average difference -0.4%). We identified seven pseudogenes on wheat 3DL that were intact genes in *Ae. tauschii* 3L, indicating a potential recent origin (Additional File 3: Table S8). Three of these *Ae. tauschii* 3L genes were expressed, but none were in wheat, confirming their identification as pseudogenes. Available methylation data for 4 of these gene

pairs showed a large increase in CpG methylation in the wheat pseudogenes compared to their functional *Ae. tauschii* counterparts.

Chromatin accessibility

Differences in chromatin accessibility are key factors in chromosome-scale patterns of gene expression changes seen in dosage compensation [27] and in large-scale transcriptional reprogramming [28]. We therefore carried out ATAC-seq [29] in nuclei prepared from hexaploid wheat and diploid *Ae. tauschii* AL8/78 leaf protoplasts. Additional File 3: Fig. S4 shows the profile of ATAC fragment sizes on chromosomes 3L and 3DL. The ~10.5 bp periodicity reflects cleavage of the DNA helix and a trace of single and double nucleosome spacing can be seen in the 3L ATAC fragment length frequency plot. In both *Ae. tauschii* and wheat, normalised density plots of ATAC peaks showed that these were mainly found in 5'UTR + promoter regions (Additional File 3: Fig. S5), consistent with more accessible chromatin in putative transcriptional regulatory regions observed in mammalian cells [29]. Peak lengths showed that regions of accessible chromatin extended to over 5 nucleosomes, with a peak at 1-2 nucleosome spacing. In *Ae. tauschii* leaf nuclei 4,960 ATAC peaks on 936 genes were identified on diploid 3L and 2,970 ATAC peaks in 1,187 genes were identified on hexaploid 3DL leaf nuclei (Additional File 3: Table S9). Interestingly, 26.53% of wheat and 28.64% of *Ae. tauschii* ATAC peaks mapped to intergenic regions. Approximately 60% of these intergenic ATAC peaks were in regions that were not annotated as repeats, while nearly 75% of the ATAC peaks that mapped to annotated repeats were found over simple sequence repeats (SSR) (Fig. 5A). Only a small proportion of the annotated repeats on 3DL and 3L had accessible chromatin, the largest of which was 8% of SSR on 3L (Fig. 5B).

Figure 5: Distribution of chromatin accessibility across intergenic regions of hexaploid wheat 3DL and *Ae. tauschii* 3L chromosome arms. (**A**) The proportion of open chromatin sequences in intergenic regions. The left pie charts show the proportion of accessible chromatin sequences in intergenic space that has no annotated features and annotated repeats on both chromosome arms. The right pie charts show the proportion of different annotated repeats that have accessible chromatin. (**B**) The graph shows the percent of annotated repeats that have accessible chromatin in hexaploid wheat 3DL and *Ae. tauschii* 3L chromosome arms.

ATAC peak length density plots (Fig. 6A) showed an overall restriction of accessible chromatin in genic regions of hexaploid 3DL compared to diploid 3L, with highly significant differences in genes with both proportionately reduced and differential patterns of gene expression. (Fig. 6B). Typical patterns of reduced ATAC peak coverage of promoters of two syntenic pairs of wheat genes compared to their *Ae. tauschii* counterparts involved loss of additional peaks and reduced width of a common peak are shown in Additional File 3: Fig. S6. Peak length distributions mapped to TSS of hexaploid 3DL and diploid 3L genes showed approximately 60% of accessible regions were found within 100kb of the TSS in diploid 3L genes. In contrast, 50% of accessible chromatin was found within 3kb of hexaploid 3DL gene TSSs (Fig. 6C). This large-scale restriction of genic chromatin accessibility in hexaploid 3DL compared to diploid 3L genes mapped across the entire chromosome arms (Fig. 7).

Figure 6: The distribution of accessible chromatin on wheat chromosome 3DL and *Ae. tauschii* chromosome 3L. (**A**) Normalized ATAC peak length enrichment for four classes of chromosomal regions on Paragon wheat chromosome 3DL and *Ae. tauschii* 3L. The chromosomal regions are intergenic; CDS+introns; 5'UTR+2kb upstream putative promoter region; 3'UTR+2kb downstream. ATAC peak length distributions are shown in base-pairs on the horizontal axis. The colour scale shows ATAC peak frequency distribution per bin. (**B**) Box plots of ATAC peak lengths across intergenic regions, genes with proportionately reduced expression patterns, and genes showing differential expression between wheat 3DL and *Ae. tauschii* 3L. The significance of ATAC peak length differences was assessed by one-way ANOVA. Peak length differences for balanced genes between wheat at *Ae. tauschii* were significant at 6.99E-68, and

1.34E-10 for differentially expressed genes. (**C**) Normalised distance distribution of ATAC-sequence peaks relative to the TSS of genes in hexaploid wheat and diploid *Ae. tauschii* genes.

Figure 7: ATAC peak length distributions across syntenic genes with either proportionately reduced expression patterns (upper panel) or differential expression patterns (lower panel) across chromosome 3DL and 3L. Red lines and dots mark wheat ATAC peak lengths on the promoter+5'UTR regions of genes, while blue lines and dots mark ATAC peak lengths on the promoter+5'UTR regions of *Ae. tauschii* genes.

Of the 683 genes with differential ATAC peaks between hexploid 3DL and diploid 3L*,* 133 of 159 genes (84%) that were differentially expressed in leaf tissue (the tissue used for ATAC-seq) also had differential ATAC peaks (Fig. 8; Additional File 3: Table S10). Of these, most different ATAC peaks occurred in the 5'UTR+promoter regions. These analyses show that the majority of DEGs in hexaploid 3DL had reduced chromatin accessibility mainly over their 5' UTR5+promoter regions. These patterns indicate chromatin accessibility influenced differential gene expression.

Figure 8: Relationships between differentially expressed genes and differential ATAC peaks. The Venn diagram shows 683 genes with differential ATAC peaks on any region of genes on hexaploid 3DL and diploid 3L*.* 133 of 159 genes that are differentially expressed in leaf tissue (the tissue used for ATAC-seq) have differential ATAC peaks. Of these, most differential ATAC peaks occurred in the 5'UTR+promoter region (shown as UTR5).

Discussion

We used long-range assemblies and detailed annotations of homologous chromosome arms of diploid *Ae. tauschii* (3L) and hexaploid wheat (3DL) to characterize the expression of precisely defined syntenic gene pairs from these chromosome arms in diploid and hexaploid genome contexts. Approximately 70% of expressed gene pairs showed a proportionate reduction of gene expression in the hexaploid chromosome arm to 40% of that in the diploid chromosome. This

pattern of reduced expression probably reflects balancing gene expression, in which approximately 70% of 1:1:1 AABBDD homoeologs were shown to be expressed at an approximate mid-point level [24]. Similar overall reductions in expression have been observed in newly formed wheat hybrids [14,30,31], suggesting a global re-alignment of gene expression to near-diploid levels is an early consequence of hexaploidy. The remaining 30% of gene pairs in diploid 3L and hexaploid 3DL exhibited differential expression patterns that were significantly higher or lower in the hexaploid 3DL context compared to the diploid 3L context. This proportion is also similar to that observed in analyses of complete wheat gene sets in the hexaploid genome [24]. We were not able to identify any relationships between sequence divergence in putative promoter or gene sequences between 3L and 3DL gene pairs that may account for these differential expression patterns. A significant proportion of DEGs were expressed during the later stages of grain development, consistent with the differential contributions of the AA, BB and DD genomes to functional modules involved in seed development identified by co-expression analyses [32]. Enrichment of promoter motifs in these 87 DEGs is consistent with a model in which transcriptional regulation from the AABB genomes may differentially regulate DD genes during grain development. Such emergent patterns of *cis-trans* interactions in polyploid genomes have been modelled as an important consequence of polyploidy [33,34].

Differences in gene methylation have been proposed to contribute to differences in gene expression between progenitor and allopolyploid species [35,36]. Only 11% of DEGs had different gene and promoter methylation patterns that might cause altered expression, indicating a relatively minor influence of methylation differences on gene expression patterns in 3DL and 3L. Of the seven pseudogenes found in 3DL that had an intact homolog in 3L, bisulphite sequence data for four showed large increases in CpG methylation. This is consistent with extensive methylation of pseudogenes seen in many plant species [37,38]. Whether DNA methylation is a cause or consequence of pseudogene formation is not known in these examples.

Given the lack of evidence for differences in sequence composition (including gene loss and pseudogenization) or DNA methylation that could account for the major patterns of altered gene expression observed across chromosomes 3L and 3DL, what mechanisms may be responsible? Changes in small RNA and chromatin, as measured by chromosome immunofluorescence [14], accompany the formation of new allotetraploid wheat lines, suggesting some forms of chromatin modification may contribute to altered gene expression in wheat allopolyploids. Dynamic interplay between nucleosomes, transcription factors and chromatin remodelling proteins alters physical access to DNA in chromatin [39] and provides a direct measure of chromatin states involved in gene expression, such as the occupation of promoter and enhancer sequences by transcription factors and other proteins. ATAC sequencing [29] was used to assess chromatin accessibility in leaf nuclei of hexaploid wheat and diploid *Ae. tauschii*. Chromatin in promoter and 5'UTR regions of genes in the diploid context had more accessible chromatin, both in terms of peak numbers and peak lengths, than in the hexaploid context. This restriction of chromatin accessibility in the hexaploid context extended across the 3DL chromosome arm, encompassing genes with both proportionately reduced and differential expression. Proportionately reduced gene expression on 3DL was correlated with reduced chromatin accessibility compared to diploid 3L. Differences in chromatin accessibility, as measured by ATAC, are thought to be due to passive competition for DNA between nucleosomes and transcription factors, chromatin remodelling and architectural proteins [39]. It is possible that in the hexaploid context reduced chromatin access across genic regions of 3DL may be due to altered competition for DNA between increased nucleosome formation or reduced transcription factor levels, or a combination of both.

The overall similarities in chromatin accessibility in intergenic regions of both 3L and 3DL may be due to higher-order nucleosome packaging in heterochromatin, which is characteristic of intergenic DNA in grass genomes [40]. The relatively high levels of chromatin accessibility in SSRs may reflect possible altered nucleosome interactions with these atypical sequences, as Alu repeats influence nucleosome spacing in human cells [41].

The introduction of a divergent set of gene regulatory proteins from the 4Gb *Ae. tauschii* genome into a 12Gb tetraploid nucleus may lead to altered interactions between the new set of transcription factors and nucleosomes, thus altering chromatin accessibility. Models of homoeolog expression patterns in allopolyploids that include varying affinities and concentrations of transcription factors for gene regulatory sequences in an allopolyploid showed a strong effect of inter-genome interactions on altered gene expression [34]. Recently, the conformation of cotton chromosomes was shown to be strongly affected by polyploidy, which altered Topologically Associating Domain (TAD) boundaries and chromatin states [42]. Similar changes may occur on wheat upon polyploidization.

Potential Implications

We have identified chromosome-wide changes in chromatin accessibility in a pair of homologous Triticeae chromosome arms in diploid and hexaploid genome contexts that may establish and maintain the large-scale differences in gene expression observed upon formation of polyploid genomes. A wide range of chromatin analysis methods are currently available for studying genome-scale changes in chromatin in newly-formed polyploids to further explore mechanisms that impart new patterns of gene expression in polyploid genomes. These analyses will be able to establish comprehensive mechanisms that explain the rapid emergence and stable maintenance of new traits in polyploid crop plants such as bread wheat.

Methods

Gene synteny

Ae. tauschii AL8/78 genome pseudomolecules and their gene annotations were from [21]. 1,266 additional genes were identified on chromosome 3L using wheat 3DL gene models and *Ae.*

tauschii RNAseq and transcript assemblies to assign a total of 4,121 genes to chromosome 3L. Similarity searches between wheat and several other sequenced grass species were performed using BLAST+ (v2.6.0; parameter: -evalue 1e-10 -outfmt 6 -num_alignments 5) [43], and gene synteny and collinearity were detected using MCScanX with default settings [44], and plotted using VGSC (v1.1) [45].

Transcription Analyses

Reads were mapped to the complete Triticum3.1 genome assembly with 3DL assemblies replaced by our chromosome 3DL pseudomolecule. Expression differences between wheat and *Ae. tauschii* used the HISAT2-StringTie pipeline [46] to compute Transcripts Per Million (TPM) values (described online at [47]). Absolute quantitative RT-PCR was used to validate TPM value comparisons between the diploid and hexaploid chromosome arms. Fourteen non-DEG genes were identified and two pairs of primers for RT-PCR were designed by the HomoeologPrimer (RRID: SCR_017559; Additional File 5). One pair was designed to amplify all three gene copies in hexaploid wheat and the single copy in diploid *Ae. tauschii*, and a second pair was designed to specifically amplify only the DD genome copy. Triplicate samples of 50,000 leaf protoplasts from wheat and *Ae. tauschii* AL8/78 from young leaves were collected and RNA extracted. Quantitative PCR was performed on cDNA using a LightCycler 480 System (Roche), and a standard curve of 8-80m molecules of the 5,340-bp plasmid pETnT was used to estimate absolute transcript levels.

Bisulphite sequencing

A full description is provided on protocols.io (Available at [48]). Bisulfite converted wheat paired end sequences were aligned to wheat genome assemblies using Bismark (version 0.18.1) [49]. The methylation status of each cytosine residue across the sequences was identified using the Bismark methylation extractor tool and the percentage of reads methylated per cytosine residue across the wheat 3DL and *Ae. tauschii* sequences were calculated.

ATACseq sequencing

A protocol adapted for wheat and *Ae. tauschii* leaf nuclei is described (see details at [50]) using our ATACseqMappingPipeline (RRID: SCR_017558). Duplicate reads were removed using the Picard tools MarkDuplicates program [52]. All reads aligning to the forward strand were offset by +4 bp, and all reads aligning to the reverse complement strand were offset by -5 bp [53]. ATAC-Seq peak regions of each sample were called using MACS2 (v2.1.2_dev) [54] and only peak areas generated in all three independent experiments were used in subsequent analyses. ATAC-Seq peaks for which the distance between proximal ends was less than 10 base pairs were merged. Four classes of peak areas were assessed for chromatin accessibility: 5'UTR + promoter (including from the ATG to 2kb upstream), CDS (the gene coding region and predicted intron sequences), 3'UTR + downstream (including the 3' UTR to 2kb downstream) and intergenic (regions >2kb distance from genes).

Availability of supporting data

The chr3DL assembly and annotation data generated in this study have been submitted to the EBI European Nucleotide Archive (ENA) database (https://www.ebi.ac.uk/ena) under accession number PRJEB23358. Paragon RNAseq data is under PRJEB29855; *Ae tauschii* AL8/78 RNA-seq data is under PRJEB23317 as described in the *Ae. tauschii* genome paper [21]. *Ae tauschii* Clae 23 leaf RNA-seq data is under PRJEB29859; *Ae tauschii* ENT336 leaf RNA-seq data is under PRJEB29860; Paragon ATAC-seq data is under PRJEB29868; AL8/78 ATAC-seq data is under PRJEB29869. Gene methylation data for *Ae tauschii* AL8/78 3L and Paragon wheat 3DL data is under PRJEB31186.

Additional Files

Additional File 1. Supplementary Methods

Additional File 2. Chromosome Arm Sequence Assembly

Additional File 3. Supplementary Figures and Tables

Additional File 4. Gene Annotation

Additional File 5. Quantitative Gene Expression

Additional File 6. Differentially Expressed Genes in Developing Grain

Abbreviations

ATAC: assay for transposase-accessible chromatin; BAC: bacterial artificial chromosome; CDS: coding sequence; DAP: days after pollination; DEG: differentially expressed gene; DMR: differentially methylated region; EST: expressed sequence tag; GO: gene ontology; IWGSC: International Wheat Genome Sequencing Consortium; NR: non-redundant; SSR: simple sequence repeat; TAD: topologically associating domain; TE: transposable elements; TPM: transcripts per million; TSS: transcriptional start site; TTS: transcription termination site; UTR: un-translated region

Competing interests

The authors declare that they have no competing interests.

Funding

This work was supported by BBSRC ERA-CAPS grants BB/N005104/1, BB/N005155/1 "INTREPID" to MWB and AH. MWB was also supported by BBSRC Institute Strategic Programme Grants GRO (BB/J004588/1) and GEN (BB/P013511/1) to MWB.

Authors' contributions

F-HL and MWB conceived and managed the research. F-HL conducted bioinformatics analyses. NMcK and F-HL carried out laboratory work. L-JG performed DNA methylation analyses. AH and

M-CL provided material and advice prior to publication. MWB, L-JG and F-HL wrote the paper with contributions from NMcK.

Acknowledgements

We are grateful to project IOS1238231 of the US National Science Foundation for providing access to unpublished sequence of the *Ae. tauschii* genome. We are also grateful to Dr Tarang Mehta from the Earlham Institute for advice on ATAC protocols. We thank the sequencing teams at The Earlham Institute, Cold Spring Harbor Laboratory and University of Liverpool CGR for their expert and timely generation of sequencing data.

References

1. Otto SP. The evolutionary consequences of polyploidy. Cell. 2007;131:452–62.

2. Jiao Y, Wickett NJ, Ayyampalayam S, Chanderbali AS, Landherr L, Ralph PE, et al. Ancestral polyploidy in seed plants and angiosperms. Nature. 2011;473:97–100.

3. Wang J, Tian L, Lee H-S, Wei NE, Jiang H, Watson B, et al. Genomewide nonadditive gene regulation in Arabidopsis allotetraploids. Genetics. 2006;172:507–17.

4. Boatwright JL, McIntyre LM, Morse AM, Chen S, Yoo M-J, Koh J, et al. A Robust Methodology for Assessing Differential Homeolog Contributions to the Transcriptomes of Allopolyploids. Genetics. 2018;210:883–94.

5. Wendel JF, Lisch D, Hu G, Mason AS. The long and short of doubling down: polyploidy, epigenetics, and the temporal dynamics of genome fractionation. Curr Opin Genet Dev. 2018;49:1–7.

6. Salman-Minkov A, Sabath N, Mayrose I. Whole-genome duplication as a key factor in crop domestication. Nat Plants. 2016;2:16115.

7. King J, Armstead IP, Iain Donnison S, Roberts LA, Harper JA, Skøt K, et al. Comparative Analyses Between Lolium/Festuca Introgression Lines and Rice Reveal the Major Fraction of Functionally Annotated Gene Models Is Located in Recombination-Poor/Very Recombination-Poor Regions of the Genome. Genetics. Genetics; 2007;177:597–606.

8. Chen ZJ. Molecular mechanisms of polyploidy and hybrid vigor. Trends Plant Sci. 2010;15:57–71.

9. Ding M, Chen ZJ. Epigenetic perspectives on the evolution and domestication of polyploid plant and crops. Curr Opin Plant Biol. 2018;42:37–48.

10. Renny-Byfield S, Rodgers-Melnick E, Ross-Ibarra J. Gene Fractionation and Function in the Ancient Subgenomes of Maize. Mol Biol Evol. 2017;34:1825–32.

11. Feldman M, Levy AA. Origin and Evolution of Wheat and Related Triticeae Species. Alien Introgression in Wheat. 2015. p. 21–76.

12. Marcussen T, Sandve SR, Heier L, Spannagl M, Pfeifer M, The International Wheat Genome Sequencing Consortium, et al. Ancient hybridizations among the ancestral genomes of bread wheat. Science. American Association for the Advancement of Science; 2014;345:1250092.

13. Chagué V, Just J, Mestiri I, Balzergue S, Tanguy A-M, Huneau C, et al. Genome-wide gene expression changes in genetically stable synthetic and natural wheat allohexaploids. New Phytol. 2010;187:1181–94.

14. Jiao W, Yuan J, Jiang S, Liu Y, Wang L, Liu M, et al. Asymmetrical changes of gene expression, small RNAs and chromatin in two resynthesized wheat allotetraploids. Plant J. 2018;93:828–42.

15. Hao M, Li A, Shi T, Luo J, Zhang L, Zhang X, et al. The abundance of homoeologue transcripts is disrupted by hybridization and is partially restored by genome doubling in synthetic hexaploid wheat. BMC Genomics. 2017;18:149.

16. El Baidouri M, Murat F, Veyssiere M, Molinier M, Flores R, Burlot L, et al. Reconciling the evolutionary origin of bread wheat (Triticum aestivum). New Phytol. 2017;213:1477–86.

17. Lu F-H, McKenzie N, Kettleborough G, Heavens D, Clark MD, Bevan MW. Independent assessment and improvement of wheat genome sequence assemblies using Fosill jumping libraries. Gigascience [Internet]. 2018;7. Available from: http://dx.doi.org/10.1093/gigascience/giy053

18. Zimin AV, Puiu D, Hall R, Kingan S, Clavijo BJ, Salzberg SL. The first near-complete assembly of the hexaploid bread wheat genome, Triticum aestivum. Gigascience. 2017;6:1–7.

19. Lu F. Chromosome 3DL Arm Assembly and Annotation [Internet]. protocols.io; 2019 Available from: http://dx.doi.org/10.17504/protocols.io.75thq6n

20. International Wheat Genome Sequencing Consortium (IWGSC), IWGSC RefSeq principal investigators:, Appels R, Eversole K, Feuillet C, Keller B, et al. Shifting the limits in wheat research and breeding using a fully annotated reference genome. Science 2018;361 eaar7191.

21. Luo M-C, Gu YQ, Puiu D, Wang H, Twardziok SO, Deal KR, et al. Genome sequence of the progenitor of the wheat D genome Aegilops tauschii. Nature. 2017;551:498–502.

22. Oñate-Sánchez L, Vicente-Carbajosa J. DNA-free RNA isolation protocols for Arabidopsis thaliana, including seeds and siliques. BMC Res Notes. 2008;1:93.

23. Luo MC, Deal KR, Akhunov ED, Akhunova AR, Anderson OD, Anderson JA, et al. Genome comparisons reveal a dominant mechanism of chromosome number reduction in grasses and accelerated genome evolution in Triticeae. Proc Natl Acad Sci U S A. 2009;106:15780–5.

24. Ramírez-González RH, Borrill P, Lang D, Harrington SA, Brinton J, Venturini L, et al. The transcriptional landscape of polyploid wheat. Science [Internet]. 2018;361. Available from:

http://dx.doi.org/10.1126/science.aar6089

25. Clavijo BJ, Venturini L, Schudoma C, Accinelli GG, Kaithakottil G, Wright J, et al. An improved assembly and annotation of the allohexaploid wheat genome identifies complete families of agronomic genes and provides genomic evidence for chromosomal translocations. Genome Res. 2017;27:885–96.

26. Wicker T, Mayer KFX, Gundlach H, Martis M, Steuernagel B, Scholz U, et al. Frequent gene movement and pseudogene evolution is common to the large and complex genomes of wheat, barley, and their relatives. Plant Cell. 2011;23:1706–18.

27. Giorgetti L, Lajoie BR, Carter AC, Attia M, Zhan Y, Xu J, et al. Structural organization of the inactive X chromosome in the mouse. Nature. Nature Publishing Group, a division of Macmillan Publishers Limited. All Rights Reserved.; 2016;535:575.

28. Miyamoto K, Nguyen KT, Allen GE, Jullien J, Kumar D, Otani T, et al. Chromatin Accessibility Impacts Transcriptional Reprogramming in Oocytes. Cell Rep. 2018;24:304–11.

29. Buenrostro JD, Giresi PG, Zaba LC, Chang HY, Greenleaf WJ. Transposition of native chromatin for fast and sensitive epigenomic profiling of open chromatin, DNA-binding proteins and nucleosome position. Nat Methods. 2013;10:1213–8.

30. Akhunova AR, Matniyazov RT, Liang H, Akhunov ED. Homoeolog-specific transcriptional bias in allopolyploid wheat. BMC Genomics. 2010;11:505.

31. Chelaifa H, Chagué V, Chalabi S, Mestiri I, Arnaud D, Deffains D, et al. Prevalence of gene expression additivity in genetically stable wheat allohexaploids. New Phytol. 2013;197:730–6.

32. Pfeifer M, Kugler KG, Sandve SR, Zhan B, Rudi H, Hvidsten TR, et al. Genome interplay in the grain transcriptome of hexaploid bread wheat. Science. 2014;345:1250091.

33. Bottani S, Zabet NR, Wendel JF, Veitia RA. Gene Expression Dominance in Allopolyploids: Hypotheses and Models. Trends Plant Sci. 2018;23:393–402.

34. Hu G, Wendel JF. Cis-trans controls and regulatory novelty accompanying allopolyploidization. New Phytol [Internet]. 2018; Available from: http://dx.doi.org/10.1111/nph.15515

35. Chen ZJ, Ni Z. Mechanisms of genomic rearrangements and gene expression changes in plant polyploids. Bioessays. 2006;28:240–52.

36. Hu Z, Song N, Xing J, Chen Y, Han Z, Yao Y, et al. Overexpression of three TaEXPA1 homoeologous genes with distinct expression divergence in hexaploid wheat exhibit functional retention in Arabidopsis. PLoS One. 2013;8:e63667.

37. Schöb H, Grossniklaus U. The first high-resolution DNA "methylome." Cell. 2006;126:1025–8.

38. Meunier J, Khelifi A, Navratil V, Duret L. Homology-dependent methylation in primate repetitive DNA. Proc Natl Acad Sci U S A. 2005;102:5471–6.

39. Klemm SL, Shipony Z, Greenleaf WJ. Chromatin accessibility and the regulatory epigenome. Nat Rev Genet [Internet]. 2019; Available from: http://dx.doi.org/10.1038/s41576-

018-0089-8

40. Baker K, Dhillon T, Colas I, Cook N, Milne I, Milne L, et al. Chromatin state analysis of the barley epigenome reveals a higher-order structure defined by H3K27me1 and H3K27me3 abundance. Plant J. 2015;84:111–24.

41. Tanaka Y, Yamashita R, Suzuki Y, Nakai K. Effects of Alu elements on global nucleosome positioning in the human genome. BMC Genomics. 2010;11:309.

42. Wang M, Wang P, Lin M, Ye Z, Li G, Tu L, et al. Evolutionary dynamics of 3D genome architecture following polyploidization in cotton. Nat Plants. 2018;4:90–7.

43. Camacho C, Coulouris G, Avagyan V, Ma N, Papadopoulos J, Bealer K, et al. BLAST+: architecture and applications. BMC Bioinformatics. 2009;10:421.

44. Wang Y, Tang H, Debarry JD, Tan X, Li J, Wang X, et al. MCScanX: a toolkit for detection and evolutionary analysis of gene synteny and collinearity. Nucleic Acids Res. 2012;40:e49.

45. Xu Y, Bi C, Wu G, Wei S, Dai X, Yin T, et al. VGSC: A Web-Based Vector Graph Toolkit of Genome Synteny and Collinearity. Biomed Res Int. 2016;2016:7823429.

46. Pertea M, Kim D, Pertea GM, Leek JT, Salzberg SL. Transcript-level expression analysis of RNA-seq experiments with HISAT, StringTie and Ballgown. Nat Protoc. 2016;11:1650–67.

47. Lu F. Transcription Analyses [Internet]. protocols.io; 2019 [cited 2019 Oct 13]. Available from: http://dx.doi.org/10.17504/protocols.io.75uhq6w

48. Gardiner L-JHA. Bisulphite Sequencing [Internet]. protocols.io; 2019 [cited 2019 Oct 13]. Available from: http://dx.doi.org/10.17504/protocols.io.75vhq66

49. Krueger F, Andrews SR. Bismark: a flexible aligner and methylation caller for Bisulfite-Seq applications. Bioinformatics. 2011;27:1571–2.

50. Lu F. Assay for Transposase-Accessible Chromatin (ATAC) Sequencing [Internet]. protocols.io; 2019 [cited 2019 Oct 13]. Available from: http://dx.doi.org/10.17504/protocols.io.75whq7e

51. Li H, Handsaker B, Wysoker A, Fennell T, Ruan J, Homer N, et al. The Sequence Alignment/Map format and SAMtools. Bioinformatics. 2009;25:2078–9.

52. Picard tools MarkDuplicates (http://broadinstitute.github.io/picard/)

53. Adey A, Morrison HG, Asan, Xun X, Kitzman JO, Turner EH, et al. Rapid, low-input, low-bias construction of shotgun fragment libraries by high-density in vitro transposition. Genome Biol. 2010;11:R119.

54. Zhang Y, Liu T, Meyer CA, Eeckhoute J, Johnson DS, Bernstein BE, et al. Model-based analysis of ChIP-Seq (MACS). Genome Biol. 2008;9:R137.

Click here to download Figure Figure 1.pdf

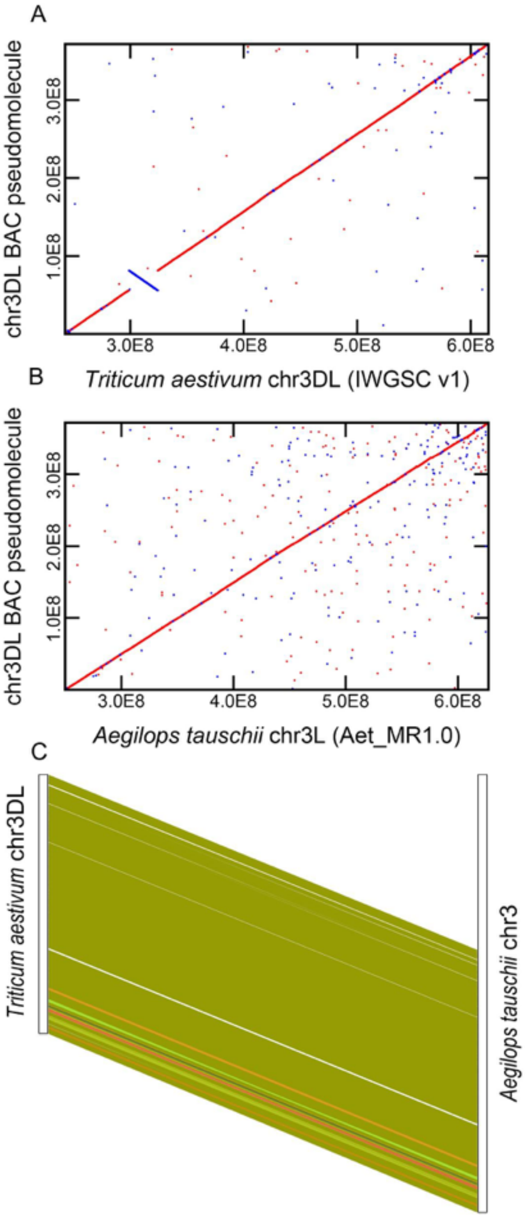

Figure 2

Click here to download Figure Figure2.pdf

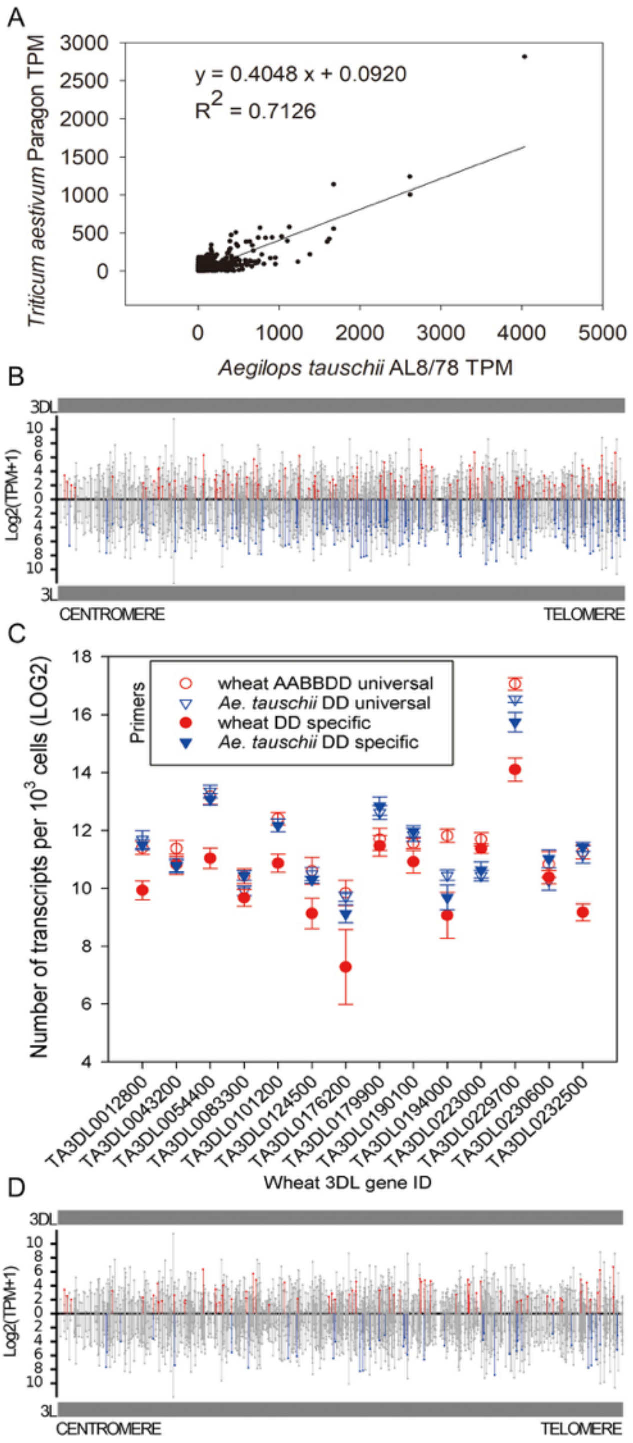

Figure 3 Click here to download Figure Figure3.pdf

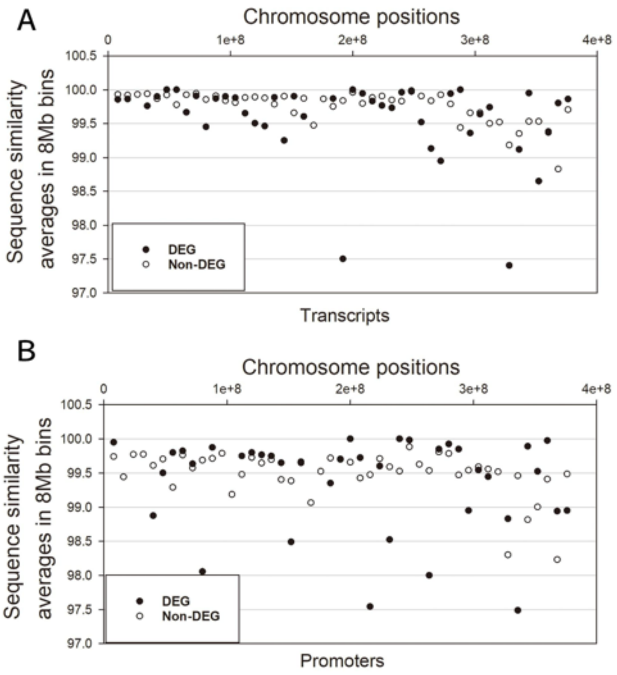

Figure 4 Click here to download Figure Figure 4.pdf

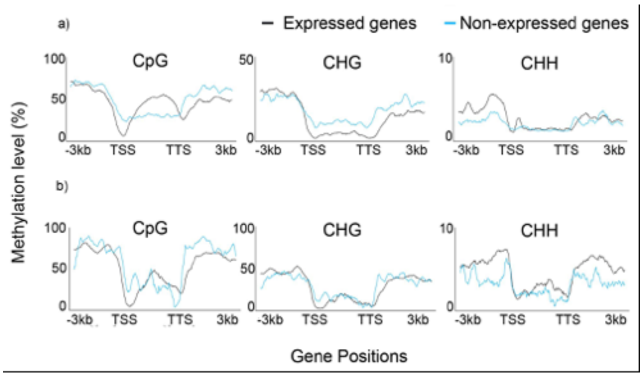

Figure 5

Click here to download Figure Figure 5.pdf

[redacted]

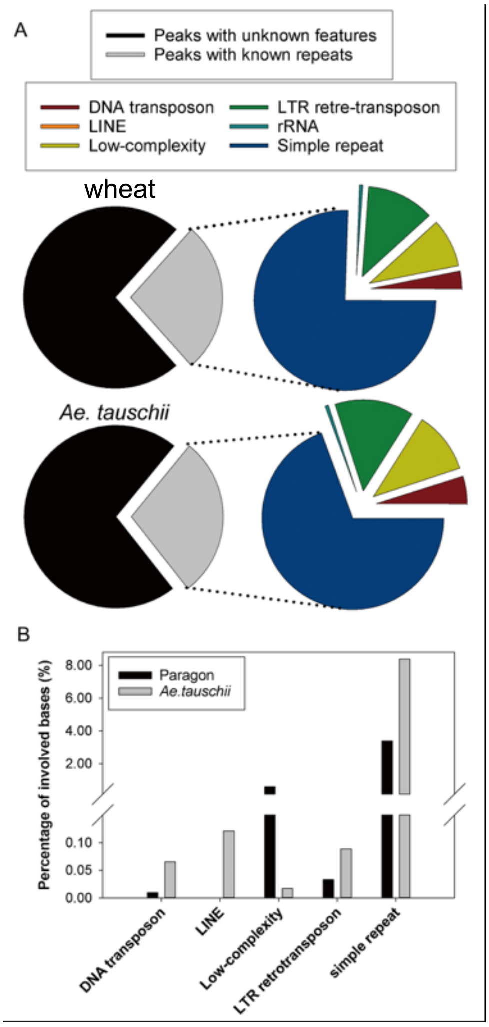

Click here to download Figure Figure 6.pdf

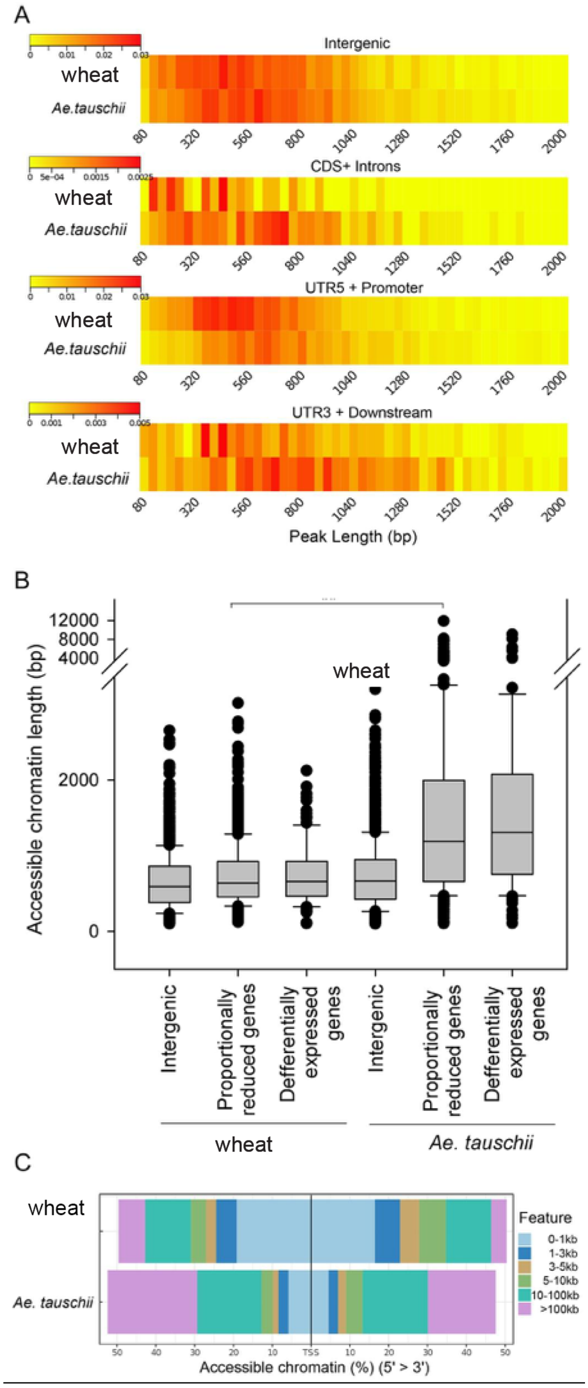

Figure 7 Click here to download Figure Figure 7.pdf

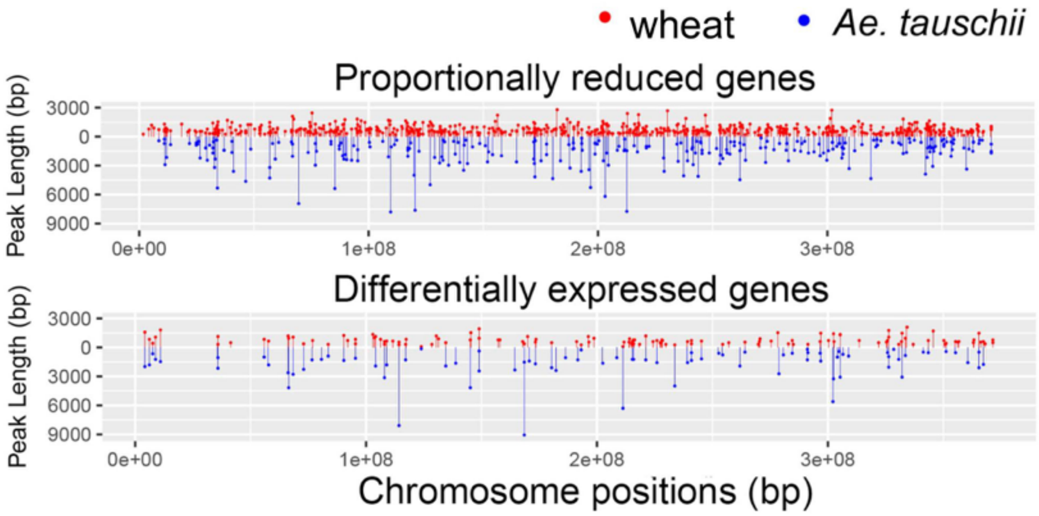

Figure 8 Click here to download Figure Figure 8.pdf

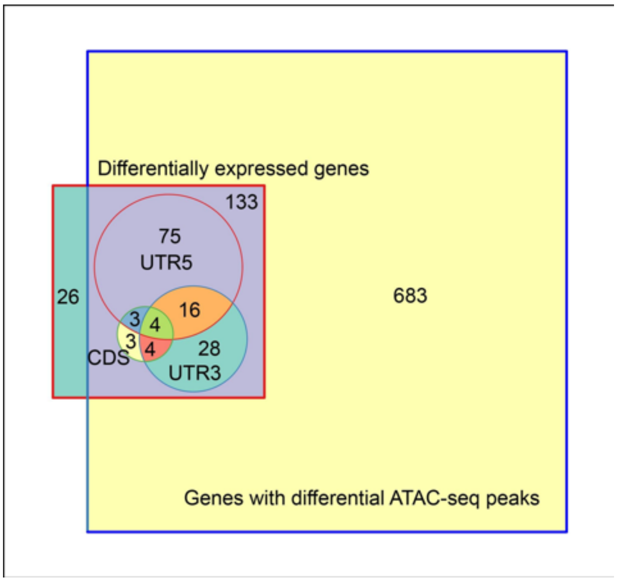

Additional File 1

Click here to access/download
Supplementary Material
Additional_File_1.pdf

Additional File 2

Click here to access/download
Supplementary Material
Additional_File_2.xlsx

Additional File 3

Click here to access/download
Supplementary Material
Additional_File_3.pdf

Additional File 4

Click here to access/download
Supplementary Material
Additional_File_4.xlsx

Click here to access/download
Supplementary Material
Additional_File_5.xlsx

Additional File 6

Click here to access/download
Supplementary Material
Additional_FIle_6.xlsx
