# Supplementary material for: Reduced chromatin accessibility underlies gene expression differences in homologous chromosome arms of diploid Aegilops tauschii and hexaploid wheat
Source: Gigascience. 2020 Jun 20;9(6):giaa070. doi: 10.1093/gigascience/giaa070 (PMC7305686; doi:10.1093/gigascience/giaa070)
Supplement: giaa070_Supplemental_Files [file giaa070_supplemental_files.zip › Additional_File_3.pdf]

Additional File 3: Supplementary Figures & Tables

Figure S1

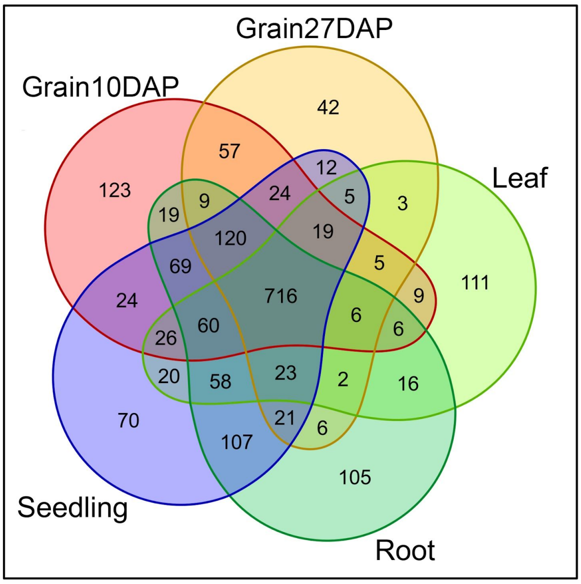

A

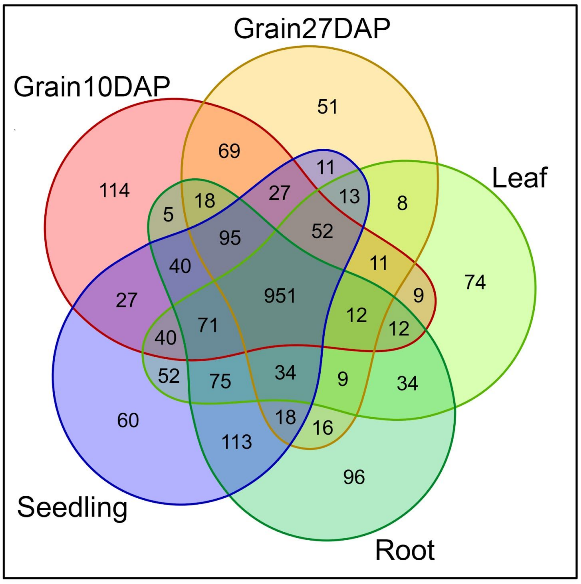

B

Figure S1: Expression profiles of syntenic genes of *Triticum aestivum* Paragon 3DL and *Aegilops tauschii* AL8/78 3L in five tissues. Syntenic genes expressed with TPM≥1 from wheat 3DL (1,893 genes, **A**) and *Ae. tauschii* chr3L (2,217 genes, **B**) among leaf, root, seedling, 10 DAP (Days After Pollination) and 27 DAP developing grain.

Figure S2

A

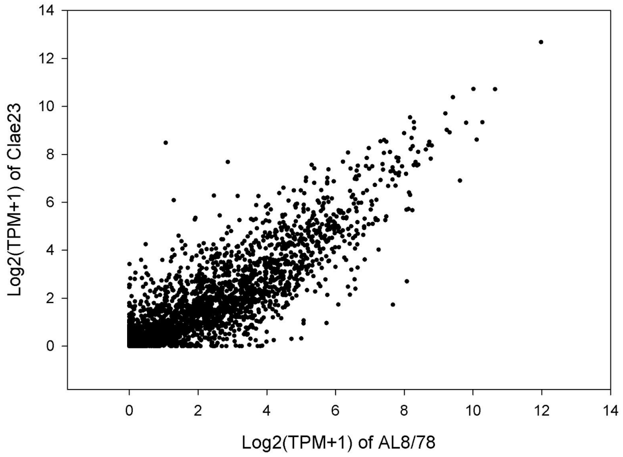

B

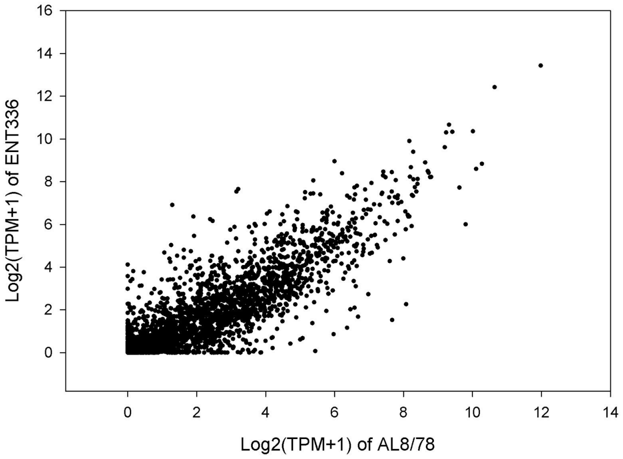

Figure S2: Comparison of expression levels of between *Aegilops tauschii* varieties AL8/78, Clae23 and ENT336.

To identify conserved differentially expressed genes (DEGs) between *Triticum aestivum* Paragon and *Aegilops tauschii* AL8/78, RNAseq data was generated from another two *Ae. tauschii* accessions, Clae23 (**A**) and ENT336 **(B**). TPM values showed a strong correlation between the pairs of accessions. (R^2 = 0.7964 for Clae23 and 0.7932 for ENT336)

Figure S3

A

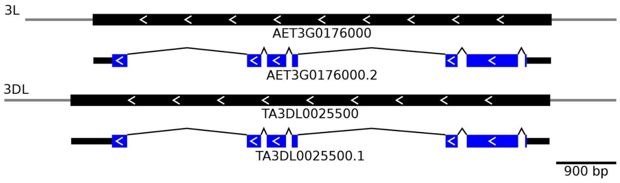

B

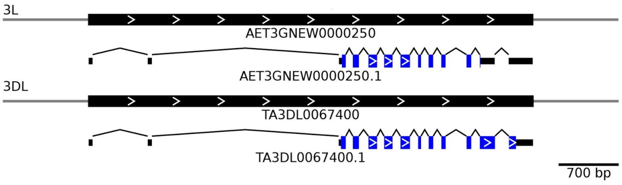

C

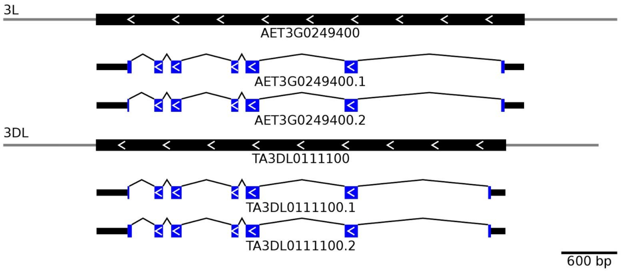

D

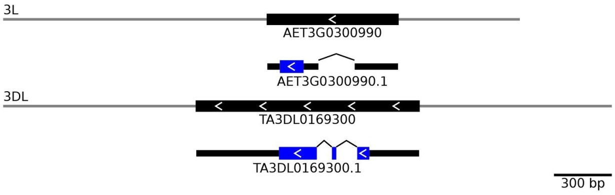

E

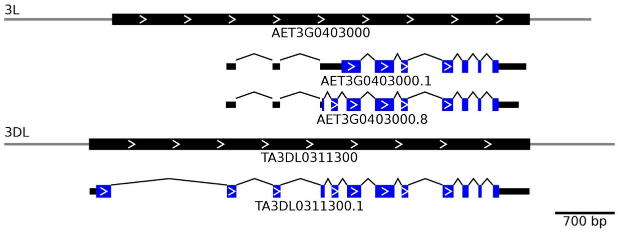

Figure S3: Illustration of gene structure differences among 106 conserved DEGs between wheat and *Ae. tauschii.*

A An example of wheat TA3DL0025500 with the same structure as its syntenic gene pair in *Ae. tauschii*, and 4 gene pairs with different gene structures: B TA3DL0067400 (alternate start codon); **C** TA3DL0111100 (alternate first exon); **D** TA3DL0169300 (exon 2 in *Ae. tauschii* missing); and **E** TA3DL0311300 (*Ae. tauschii* has premature stop codon).

Figure S4

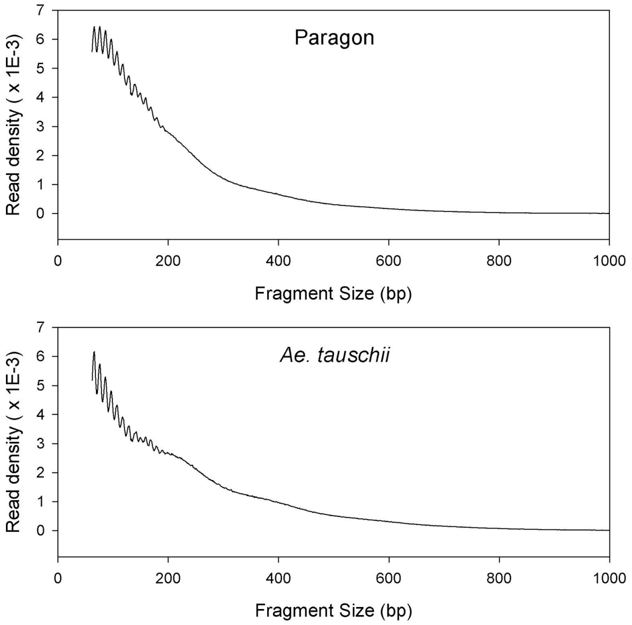

Figure S4: Size distribution of ATAC-seq fragment lengths between *Triticum aestivum* Paragon and *Aegilops tauschii* AL8/78.

The 10.5bp DNA pitch reflects the periodicity of right-handed helix in B-DNA. A trace of single and double nucleosome spacing can be seen in the 3L ATAC peaks, at approximately 200bp and 400bp.

Figure S5

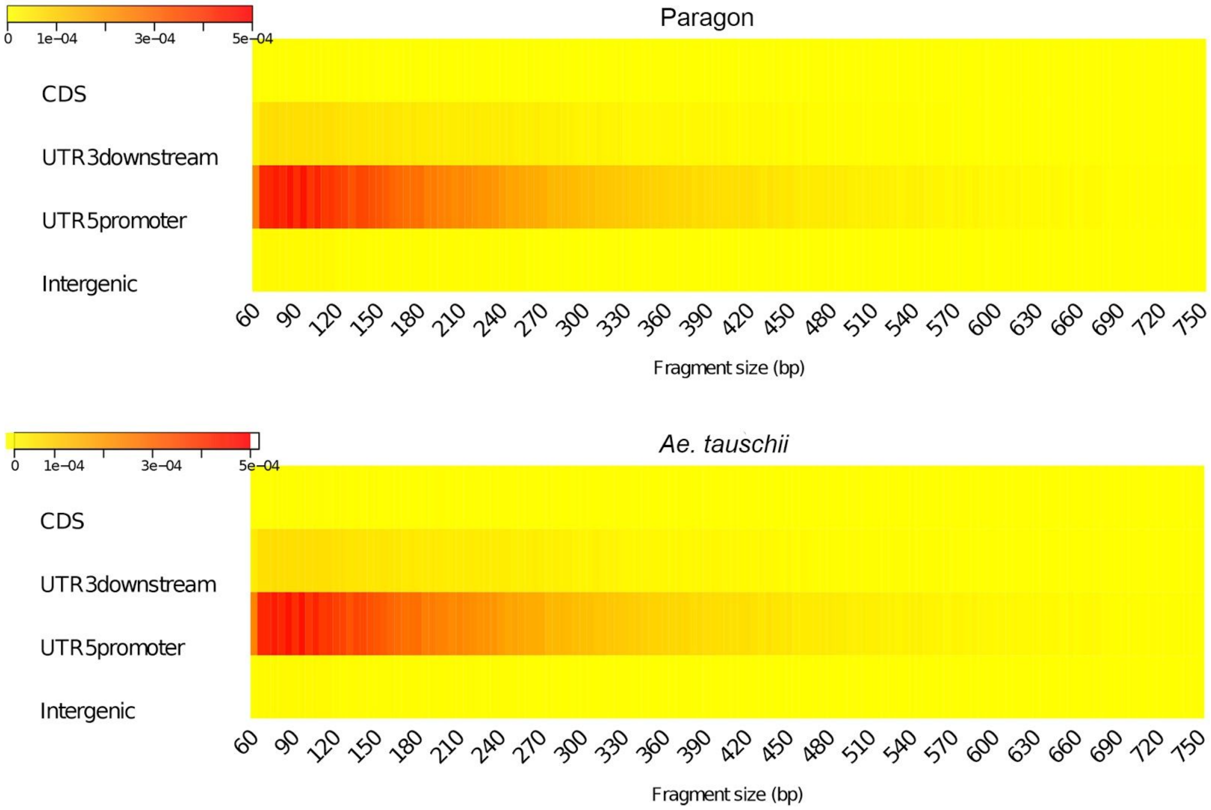

Figure S5: Normalised read enrichment for four classes of chromosome states in *Triticum aestivum* Paragon and *Aegilops ta*uschii AL8/78.

The normalised density plots of ATAC peaks showed that 5"UTR + promoter (2kb) regions had the highest read densities, followed by 3' UTR + downstream (2kb) regions, CDS + intron regions and intergenic regions.

Figure S6

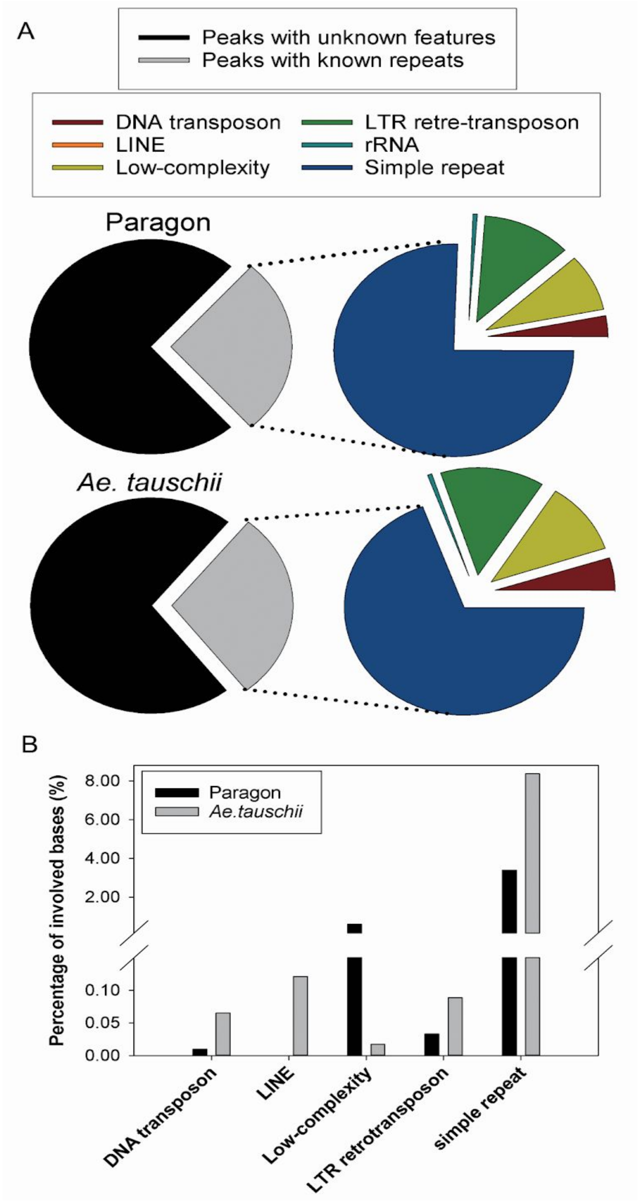

Figure S6: Distribution of chromatin accessibility across intergenic regions of hexaploid wheat 3DL and *Ae. tauschii* 3L chromosome arms. (**A**) The proportion of open chromatin sequences in intergenic regions. The left pie charts show the proportion of accessible chromatin sequences in intergenic space that has no annotated features and annotated repeats on both chromosome arms. The right pie charts show the proportion of different annotated repeats that have accessible chromatin. (**B**) The graph shows the percent of annotated repeats that have accessible chromatin in hexaploid wheat 3DL and *Ae. tauschii* 3L chromosome arms.

Figure S7

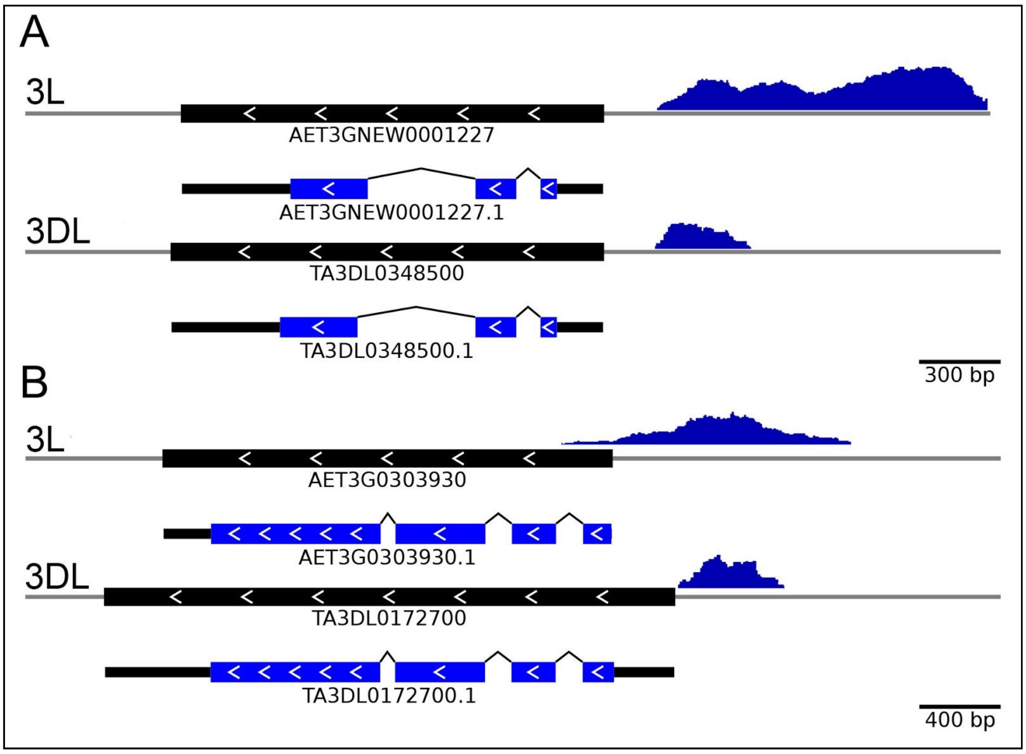

Figure S7: ATAC peaks on pairs of syntenic genes in hexaploid wheat and diploid *Ae tauschii*.

A An example of ATAC peaks on a syntenic pair of *Ae. tauschii* and wheat genes with typical proportionately reduced gene expression.

B An example of ATAC peaks on a typical pair of differentially expressed *Ae. tauschii* and wheat genes.

Table S1:

Sequence assembly summary of wheat chromosome 3DL

Scaffolds	Versions	Number	Total bases (n50)	n50 (bp)	Max (bp)
3DL PacBio	Original	2,703	409,078,553	279,970	1,657,081
	Fosill-joined	1,751	422,542,707	437,268	2,142,379
	BAC-scaffoldable	1,339	357,619,806	442,952	2,142,379
	BAC-joined	524	371,055,817	1,096,174	5,859,701
3DL BACs	Original (v2)	35,316	782,691,342	100,179	281,751
	PacBio-joined (v6)	504	371,709,278	1,176,844	5,861,578
	pseudomolecule	1	371,759,578	-	-

Table S2:

Summary of repetitive elements of *Triticum aestivum* Chinese Spring 3DL and *Aegilops tauschii* AL8/78 3L

		***Triticum aestivum* chr3DL**		***Aegilops tauschii* chr3L**	
**Class**	**Subclass**	**Length (bp)**	**Percentage**	**Length (bp)**	**Percentage**
**DNA transposon**		45,694,659	12.29%	47,299,305	12.54%
	CMC-ENSPM	41,305,883	11.11%	42,925,528	11.38%
**LINE**		1,670,347	0.45%	1,651,400	0.44%
**LTR retrotransposon**		191,094,703	51.40%	198,840,503	52.70%
	LTR-Copia	56,109,154	15.09%	57,910,706	15.35%
	LTR-Gypsy	103,525,917	27.85%	108,703,865	28.81%
**MobileElement**		956	0.00%	847	0.00%
**RC**		58,706	0.02%	73,186	0.02%
**rRNA**		19,149	0.01%	17,408	0.00%
**Simple_repeat**		1,493,362	0.40%	1,599,416	0.42%
**Low_complexity**		336,380	0.09%	345,552	0.09%
**Other**		20,280,094	5.46%	21,132,447	5.60%
**SUM**		**257806071**	**69.35%**	**267937996**	**71.01%**

Table S3:

Number of RNAseq read pairs after trimming in 5 sampled tissues of *Triticum aestivum* Paragon and *Aegilops tauschii* AL8/78

Tissues	Replicates	Paragon	AL8/78
Leaves	Replicate 1	56,816,727	50,677,603
	Replicate 2	56,452,015	55,377,053
	Replicate 3	38,670,428	57,634,414
Roots	Replicate 1	38,735,310	52,677,010
	Replicate 2	51,869,501	55,016,084
	Replicate 3	50,896,607	53,365,111
Grains (10dd)	Replicate 1	59,835,166	80,624,534
	Replicate 2	66,214,312	77,159,911
	Replicate 3	57,335,248	68,751,597
Grains (27dd)	Replicate 1	55,984,207	88,885,636
	Replicate 2	51,331,203	75,465,851
	Replicate 3	74,165,822	80,256,130
Grains (Pooled)	Replicate 1	63,778,882	60,647,054
Seedling (4dd)	Replicate 1	63,782,697	74,531,986
	Replicate 2	77,936,789	71,175,660
	Replicate 3	75,481,500	57,043,451

Table S4:

Number of differential expressed genes in 5 tissues between *Triticum aestivum* Paragon and *Aegilops tauschii* AL8/78

	Expressed genes	DEGs	Pseudogenes
Developing grain (10dd)	1,716	277 (up 147, down 130)	44
Developing grain (27dd)	1,516	173 (up 86, down 87)	38
Leaf	1,564	262 (up 112, down 150)	42
Root	1,784	327 (up 176, down 151)	44
Seedling	1,829	251 (up 144, down 107)	47
Sum	2,375	674	66

Table S5:

Mapping statistics after alignment of bisulfite treated *Triticum aestivum* Paragon and *Ae. tauschii* AL8/78 samples

		**Paragon**	***Ae.tauschii***
Number of genes mapped		2,810	3,997
C sites mapped on genes (min 10X)		846,450	458,347
C sites mapped on 3DL promoters (min 10X)		418,818	289,521
C's methylated overall			
	CpG	89.9%	87.1%
	CHG	59.4%	53.4%
	CHH	3.8%	3.5%
C's methylated genes			
	CpG	66.7%	41.2%
	CHG	13.2%	18.9%
	CHH	1.0%	3.8%
% converted		98.7	98.6

Table S6:

Number of regions for methylation analysis in *Triticum aestivum* Paragon 3DL and *Aegilops tauschii* AL8/78 3L

	**Paragon**	**AL8/78**	**Comparable regions**
Gene CpG	2,378	2,664	1,533
Gene CHG	2,467	2,643	1,586
Gene CHH	2,706	2,932	1,913
Promoter CpG	1,703	2,308	891
Promoter CHG	1,791	2,229	901
Promoter CHH	2,174	2,703	1,353

Table S7:

Methylation levels of pseudogenes in Paragon wheat 3DL and *Ae tauschii* AL8/78 3L

Methylation context	CpG %	CHG %	CHH %
Paragon wheat 3DL			
Coding genes	59.7	9.3	1.2
Coding gene promoters	65.4	30.7	3.7
pseudogenes	72.5	25.2	1.4
pseudogene promoters	78.3	43.6	2.7
Ae tauschii AL8/78 3L			
Coding genes	29.9	11.7	2.1
Coding gene promoters	62.5	38.1	5.5
pseudogenes	53.7	28.8	2.7
pseudogene promoters	65.7	42.7	4.0

Table S8:

Pseudogenes in Paragon wheat 3DL that have intact counterparts in *Ae. tauschii* AL8/78 3L

Paragon GeneID	Gene Expression	Mutations	Descriptions		CpG %	CHG %	CHH %
TA3DL0067600	-	Exon3 non-sense mutation (TAA/GAA)	MICOS complex subunit MIC60-like	-	-	-	-
TA3DL0149600	10DAP developing grain and seedling in AL8/78	Exon2 Opal mutation (TGA/CGA)	Protein ACCELERATED CELL DEATH 6-like	Paragon	76.97	0.88	0.17
				AL8/78	4.00	3.83	0.69
TA3DL0184600	10 DAP and 27 DAP developing grain, root and seedling in AL8/78	Exon4 2 bases insertion (GTGAG/GAG)	GDP-L-galactose phosphorylase 1-like	Paragon	47.30	0.37	0.39
				AL8/78	45.71	8.89	1.48
TA3DL0211300	Root in AL8/78	Exon4 non-sense mutation (TGA/GGA	Endo-1,3;1,4-beta-D-glucanase-like	Paragon	49.28	1.49	0.26
				AL8/78	12.00	3.64	1.39
TA3DL0286700	-	Exon5 one deletion (-TC/TTC)	Serine/threonine-protein phosphatase 7 long form homolog	Paragon	97.54	66.33	1.49
				AL8/78	97.35	50.81	2.32
TA3DL0299800	-	Exon2 alternative splicing	Uncharacterized	-	-	-	-
TA3DL0320700	-	Exon4 Opal mutation (TGA/CGA)	Zinc finger MYM-type protein 1-like	-	-	-	-

Table S9:

Summary of ATAC peaks and covered genes in different chromosome states of Paragon wheat and *Ae. tauschii* AL8/78

	Number of Peaks (Genes covered*)	
	**Paragon**	**AL8/78**
5'UTR+promoter	1,266 (1,098 genes)	1,739 (869 genes)
CDS+Intron	53 (42 genes)	171 (130 genes)
3'UTR+downstream	226 (211 genes)	480 (350 genes)
Intergenic	1,425 regions	2,570 regions
Total peaks	2,970 (1,187 genes)	4,960 (936 genes)

*All genes including non-syntenic genes

Table S10:

Numbers of syntenic genes covered by ATAC-seq peaks

	Paragon	AL8/78
Overall gene pairs	930 genes (159 DEGs)	
Total	774 (128 DEGs)	362(88 DEGs)
CDS	25(1 DEGs)	46(13 DEGs)
UTR3downstream	157(27 DEGs)	132(35 DEGs)
UTR5promoter	727(125 DEGs)	345(85 DEGs)
Gene pairs with differential peaks	816 genes (133 DEGs)	
CDS	69 (14 DEGs)	
UTR3downstream	251 (52 DEGs)	
UTR5promoter	684 (98 DEGs)	
