# Supplementary material for: Reduced chromatin accessibility underlies gene expression differences in homologous chromosome arms of diploid Aegilops tauschii and hexaploid wheat
Source: Gigascience. 2020 Jun 20;9(6):giaa070. doi: 10.1093/gigascience/giaa070 (PMC7305686; doi:10.1093/gigascience/giaa070)
Supplement: giaa070_Response_to_Reviewer_Comments_Original_Submission [file giaa070_response_to_reviewer_comments_original_submission.pdf]

Author's Response To Reviewer Comments

Close

Dear Editors
Here are our responses to the Reviewers' comments and requests on:
GIGA-D-19-00357
Reduced chromatin accessibility underlies gene expression differences in homologous chromosome arms of diploid Aegilops tauschii and hexaploid wheat
Fu-Hao Lu; Neil McKenzie; Laura-Jayne Gardiner; Ming-Chen Luo; Anthony Hall; Michael Webster Bevan, PhD
We have registered all our newly developed software on SciCrunch.org, and have their RRIDs included in the manuscript.
We have included several new references in the Introduction to provide a more detailed background for the new analyses.
We found the reviewer's comments to be helpful in clarifying the message of the manuscript. As ATAC-seq is a relatively new method, especially as applied to polyploids, we have spent more effort as requested in explaining the data generated by this method and how it can be interpreted. We also "re-balanced" the results and discussion section to make more of the methylation data, which we had originally de-emphasised due to the 11% of genes having differential methylation and differential expression. One remaining issue is the use of TPM for measuring gene expression in organisms with different numbers of genes. Here we validate the use of TPM using absolute quantitative PCR, and we compensate for the overall reduction of expression measured in the hexaploid in calculating differential gene expression, as done in a recent wheat gene expression paper. Nevertheless in future publications we aim to assess the use of TPM for these types of studies. It's important to point out that nearly all comparative genomics papers use TPM.
We hope the edited manuscript is now acceptable. As we show major changes in chromatin accessibility in hexaploid vs diploid chromosome arms we think our work opens up new opportunities for understanding genomic interactions. Newly available methods will make this a fruitful area for understanding such interactions.
Response to Reviewer 1:
Question (1) Non-syntenic genes (Fig. 1): Most of the 207 non-syntenic genes (~5%) are located near the chromosome end. It is unclear how the non-syntenic genes are defined, compared to 3,456 orthologous genes with >99% identity. What is the possible reason for those non-syntenic genes predominantly in the chromosome ends? Is this a general feature for all other chromosomes or the entire hexaploid genome?
Response: Those non-syntenic genes are defined as missing a syntenic counterpart on either of the diploid 3L Ae tauschii or hexaploid wheat 3DL chromosome arms. There is extensive evidence for a greater diversity of sequences, gene order and meiotic recombination towards the ends of large Triticeae chromosomes such as barley (Nature 554, p427, Figure 5) and wheat (Science 395, p1249721-1 Figure 1). These processes can erode ancestral gene order and lead to reduced gene order. It is also possible that sequence assembly errors may make a contribution to synteny loss, but as we observe high levels of synteny in repeat rich regions, which are more difficult to assemble, we think assembly differences probably make a very small contribution to reduced gene synteny in the assemblies used in this study.
Question (2) Gene expression (Fig. 2): "In total, 2,375 (68.72%) of the syntenic genes from 3DL (1,893 genes) and/or 3L (2,217 genes) were expressed at TPM≥1 in these tissues (Additional File 4)." It is unclear what is meant, and how many genes (%) are expressed, relative to what? Again, in the following sentence, "…showed a significant trend in reduction of TPM values in the hexaploid context to approximately 40% of those measured in diploid genomic context for approximately 70% of the pairs of genes." If these are syntenic (orthologous) genes, shouldn't they have the same number of denominators for expressed genes in both 3D and 3L, unless some genes are expressed specifically in 3D or 3L, which should be excluded for this analysis and discussed separately.
Answer: In each syntenic gene pair (total of 2,375 pairs) between wheat and tauschii, say x=wheat-TPM and y=tauschii-TPM, x and y may not >=1 in the same tissues sampled. That is, one (hexaploid or diploid gene) of the pairs may be TPM<1 and another TPM>=1. In the hexaploid context, 1893 genes are TPM>=1. And in tauschii, 2217 genes are TPM>=1. In each pair, if either of them TPM>=1, this pair would be included for further analysis. 40% indicates the TPM values and 70% indicates the number of

gene pairs. This means 70% of syntenic diploid genes have a reduction to 40% of TPM values measured in the hexaploid.
We have amended the manuscript to make this point clearer.
Question (3) DEGs (Fig. 2): It is unclear how this normalization of the transcriptome in 3L by multiplying "1.7604" is defined and justified. Although 70% of genes in 3D could be expressed at lower levels than those in 3L, expression of homoeologs in 3A and 3B is not examined and could be upregulated to compensate as a dosage effect, as every D homoeolog in hexaploid wheat would have additional two other homoeologs. This section of DEGs should be revisited. On a related note, image displays in Fig. 2B, D are confusing with too many lines and colors and should be simplified to highlight the DEGs.
Answer: Because hexaploid wheat TPM values were lower than that of diploid Ae tauschii TPM values, the factor 1.7604 elevates wheat TPM to a comparable level with tauschii based on a median normalization. We now explain this more clearly in the text. Regarding the expression of AA, BB homoeologs compared to DD homoeologs and orthologs, this work focussed on the effects of hexaploidization on a diploid DD chromosome. The interactions of AA, BB and DD homoeologs in the hexaploid genome context have recently been well characterised (Science 361, p662) and are referred to in this work. Indeed as this reviewer states about 70% of homoeologs display balanced expression. We want to emphasise here that we are comparing diploid with hexaploid patterns, not hexaploid-hexaploid. We did this for several reasons: 1. There is extensive conserved synteny between DD genomes in the hexaploid and diploid states due to the very recent hybridisation event between a tetraploid wheat and Ae tauschii, making it easier to identify orthologous relationships.. We made accurate long-range assemblies to ensure this conserved synteny was faithfully represented in our analyses 2. In contrast The AABB genomes, which hybridized less than 800 kya, have undergone extensive recombination and introgressions compared to their diploid progenitors. Furthermore the diploid progenitor(s) of the BB genome are not yet well characterised. This means a comparison of diploid to hexaploid is very difficult for the AA to AABB and BB to AABB chromosomes. 3. We generated a very accurate long-range assembly of 3DL in order to provide a precise basis for comparisons between hexaploid and diploid chromosomes. 4. This work provides a basis for ongoing work that compares expression in tetraploid and diploid progenitors of new synthetic wheats.
As suggested we altered Fig 2B and 2D to make the image clearer for readers.
Question They examined gene expression in 5 different tissues. Is this reduction of expression in the leaf consistent across all five tissues or different among tissue types? They reported 86 DEGs (upregulated in 3DL) during grain development. Thus, down-regulation of some genes in 3DL is not a common phenomenon and may vary during development. What about oher tissues?
Answer: The reduction of gene expression in hexaploid DD compared to diploid DD (Fig 2A) was based on data from 5 tissues. As the reviewer states each tissue type has different patterns of differential expression, and these patterns are summarized in Table S4 in Additional File 3. Concerning a description in other tissues, we focussed on describing DEGs in 27 day old developing grains as these had interesting patterns of GO enrichment relevant to new gene regulation patterns in hexaploid grains.
Question (4) Gene expression vs. sequence diversity (Fig. 3): this type of data should be in the supplemental information.
Answer: We would like to make a case for maintaining Figure 3 in the main text. The information it shows on sequence divergence is important as it shows the extent to which differential gene expression may be due to differences in promoter/gene sequences.
Question (5) DNA methylation changes (Fig. 4). The separate images (expressed vs. non-expressed genes) in 3DL and 3L do not convey a clear message. It would make a bit more sense to display methylation profiles with DEGs between 3DL and 3L, using all genes (or genes with equal expression levels) as the control. The images can be enlarged. The panels should be labeled as "A" and "B" (instead of "a" and "b").
Answer: As suggested, we changed Fig. 4 panel a,b to upper-case A,B, and we enlarged the Figure. Initially the Figure displayed methylation profiles of DEGs, but as a small proportion of genes showed differential expression and differential methylation (11%), the patterns still show no discernable differences. Please see responses to other referees in which we describe the cut-offs we used to define DMRs.
Question "Only 11.3 % of differentially methylated genes or promoters were associated with differences in gene expression between Paragon 3DL and Ae. tauschii 3L (Table 1)." Replace "associated" with "correlated" (with gene expression differences between…). Based on this number, though small, the methylation seems to play a role. Some details are unknown here, e.g., location of methylation in 5', 3' UTRs or gene body, because this would affect their data interpretation (positive, negative or uncorrelated). They do not seem to be consistent with what is stated. If methylation of DEGs was displayed, the picture would become clear? A related comment is that they used Agilent SureSelect gene capture for methylation analysis, which may have problems of capturing efficiency and representation of the sequences. This should be discussed and taken into consideration for concluding their results.

Answer: As suggested, we replaced "associated" with "correlated".
We analyzed methylation differences of promoter plus gene body for DEGs between 3L and 3DL 3DL and 3L, as summarized in Tables S6 and S7 in Additional File 3. As methylation of gene bodies and promoters can both influence gene expression, we did not further dissect this aspect of differential gene expression further due to the overall low contributions of DNA methylation to differential gene expression. Regarding the use of the Agilent capture method and its potential for altered representation of methylated genes, we have altered the Discussion by including the following sentence: " Eleven percent of DEGs had different gene and promoter methylation patterns that might alter expression, indicating a relatively minor influence of methylation differences on gene expression patterns in 3DL and 3L, which might be also be partly affected by gene capturing efficiency". More extensive use of the gene capture method has given us confidence that capture is representative of genomic patterns in wheat while being efficient and cost effective.
Question (6) Chromatin accessibility (ATAC-seq) (Figs. 5 and 6). Data in Fig. 5 dealt with repeats and ATAC-seq peaks, which do not have much to do with gene expression and should be in the supplementary information.
Answer: As suggested, we moved Figure 5 to Additional File 3. However, we feel that our observations of chromatin accessibility in repeats is interesting and has not, to the best of our knowledge, been reported previously. We therefore wish to maintain our Results and Discussion sections on chromatin accessibility of repeat regions.
Question Proportionally reduced expression vs. differentially expressed genes. It is unclear how these two groups of genes are defined. Based on results in Fig. 6, a main difference between 3DL and 3L is that the chromatin accessibility peaks are shorter in 3DL than in 3L. If one assumes close chromatin accessibility sites (to TSS) are important to gene expression, should the conclusion be opposite to what was noted in the manuscript? An important parameter is the peak intensity (or fold enrichment of peaks), which determines the chromatin accessibility, but this has not been examined, which could be a major problem for their overall conclusion.
Answer: These definitions can be found in 'Gene expression patterns in diploid 3L and hexaploid 3DL' and 'Differentially expressed genes' sections in Results. Regarding our use of ATAC peak length to measure chromatin accessibility, this is the standard measure of chromatin accessibility according to Buenrostro et al Nature Methods 2013. More recently a review of different methods for ATAC sequence analyses (Yan et al Genome Biology 21:22) concludes that our work follows current best practice. This includes mapping Nextera reads of purified genomic DNA to account for any bias in Nextera specificity, and the use of triplicated biological replicates. This establishes the specificity of sequence reads- we used fold enrichment compared to the baseline of purified DNA. For peak calling we used MACS2 paired - end with shifted sequences to off-set Nextera primer positions. MACS2 defined peaks according to read distribution shape, and adjacent peaks (closer than 10bp) were merged. The key feature revealed by ATAC-seq is peak length (multiples of nucleosomes). ATAC peak lengths are widely used as measures of chromatin accessibility as they are independent of sequence read depth. Notability we get the same peak length results using different amounts of Nextera reagent, showing that the results are robust, independent of read depth (intensity) and fully in line with all other ATAC analyses. We do not have sufficient knowledge at this stage to assume that accessible chromatin close to TSS are more important for gene expression than other regions eg the CDS. We do not understand the reviewer's statement that the conclusion that reduced chromatin accessibility as assessed by peak length should be opposite to that noted in the manuscript. Our interpretation is that more open chromatin across different regions of genes in the diploid chromosome arm is consistent with higher levels of gene expression. However there is not a direct correlation between more open chromatin and gene expression as, for example, there may be increased access of transcriptional repressors.
Question A minor note: both "proportionally" and "proportionately" are interchangeably used in the Abstract and text. Should they use one form?
Answer: We have changed proportionally" to "proportionately" throughout the text.
Question (7) Again in Fig. 7, it is unclear how the peak length correlates with expression of a gene (and see the comment above).
Answer: We addressed this concern in Question 6 above.
Question (8) Based on the data in Fig. 8, the correlation between chromatin accessibility and DEGs is not as straightforward as stated in the manuscript. Only 75 genes correlate with differential ATAC-seq peaks in UTRs, plus another 28 in 3' UTRs, which could be 5' UTRs of neighboring genes. They should revisit their conclusions (about relative contributions of DNA methylation and chromatin accessibility) in the Title, Abstract, and the rest of MS.
Answer: We wish to clarify that there are a total of 159 DEGs covered by ATAC-seq data in Figure 7, and 133 of them have differential ATAC-seq peaks, of which 98 (75+16+3+4) genes with differential peaks in UTR5, 52 (28+16+4+4) genes in UTR3 and 14 (3+3+4+4) genes in CDS. A comparison of these numbers to the 11 genes with differential methylation supports our focus on chromatin accessibility as a

significant factor in differences in gene expression between the diploid and polyploid states. Differential ATAC peaks are therefore more correlated with DEGs than methylation differences. As requested we will include more discussion of the role of methylation. With respect to the overlaps of UTR5 and UTR3 between genes, it should be noted that the average intergenic span in wheat is over 100kb. We therefore think that these functional overlaps between gene regulatory regions may be minimal.
Response to Reviewer 2:
Question 1. In the part of "Assay for Transposase-Accessible Chromatin (ATAC) Sequence Datasets," "ATAC-seq reads matching mitochondrial, and chloroplast genomes were identified and removed using the Triticum aestivum chloroplast genome (GenBank accession No. NC_002762), Triticum aestivum mitochondrial genome (GenBank accession No. AP008982) and the Ae. tauschii chloroplast genome (GenBank accession No. NC_022133). ". It is unclear why the ATAC-seq reads matching Ae. tauschii mitochondrial genome are not removed.
Answer: The Ae. tauschii mitochondrial genome of Ae tauschii has not yet been sequenced. Please refer to the NCBI website
(https://www.ncbi.nlm.nih.gov/genome/browse#!/organelles/). Therefore we used the sequence wheat mitochondrial genome instead. This was effective due to the very high similarities of grass mitochondrial sequences (BMC Genomics. 2014;15:1–16). We also identified non-nuclear ATAC peaks from their extremely high read depth in the BAM files. It is important to note that the main contribution
Question 2. The RNA-seq data of roots, seedlings, and developing grains is not necessary, as the tissues used in bisulfite-seq and ATAC-seq are only leaves. Moreover, the author did not clarify whether the leave materials used in bisulfite-seq, ATAC-seq, and RNA-seq are completely the same, which is vital to analyze the relationship among three sets of data.
Answer: To confirm, the same leaf samples were used for RNAseq and bisulphite sequencing. Exactly the same aged leaves grown in the same chamber were used for protoplasting and nuclear preparations for ATAC. All samples were biological triplicates for reproducibility. Therefore the leaf data is as comparable as possible. We have included a more precise explanation of this in the Methods section, while Additional File 1 describes the methods used in detail. We think it is important to include a wider range of RNAseq data to support the the observations of balancing versus differential expression in more than one tissue. Furthermore, RNAseq from developing grains provided insights into differential gene expression that influences grain development, which is an agronomically significant consequence of the DD genome to bread wheat.
Question 3. In Figure 3, DEGs have relatively less sequence similarity compared to non-DEGs. Statistical analysis should be performed to test if the difference is significant or not.
Answer: This is a binning problem. For those non- DEGs in an 8 Mbp bin, the high similarity value averages out low values in that bin, mostly because there are so many high similarity values in that bin. But for DEGs, there are a few similar numbers in that bin, making the plot less informative as the lowest value will reduce the average. Figure 3 Legend has been changed to clarify this and to include statistical analyses.
Question 4. The author concludes that DNA methylation only plays a minor role in reduced gene expression in hexaploid wheat, but the analysis of bisulfite sequencing data is not perfect enough. First, the description of data analysis is not detailed enough. In part defining the differential methylated region, "DMRs were defined if a CpG region showed a difference in methylation of 50% or more ($q < 0.05$), a CHG region showed a difference of 25% or more or a CHH site showed a difference of 10% or more. There is no reference shown here. Is it possible that the definition of DMR is too strict, resulting in a poor relationship between reduced gene expression and the distribution of DMRs?
Answer: We used a standard approach that is widely used to identify differential DNA methylation in order to maintain coherence with previous work (Akalin et al., 2012). Please note these conservative measures are widely used and as used here here stricter thresholds actually increase the strength of the relationships between gene expression and DMRs. Our wheat specific thresholds were defined from a previous analysis of DNA methylation in wheat (Gardiner et al., 2019). In specific examples of major differences in gene methylation such as pseudogenes we provide detailed comparisons on a gene-by gene basis. We have now added these two references to the text to clarify the methods that were used. The methods section has been modified to include the following clarification: “DMRs were defined if a CpG region showed a difference in methylation of 50% or more ($q < 0.05$), a CHG region showed a difference of 25% or more or a CHH site showed a difference of 10% or more (Gardiner et al., 2019; Akalin et al., 2012).”
Question 5. In figure 4, part A of bread wheat and B of Aegilops tauschii should merge into one part, and then the difference between Aegilops tauschii and bread wheat will be easy to see. According to the result in figure 4, the levels of CHG and CHH methylation in the promoter and 3 UTR regions in bread wheat are lower than in Aegilops tauschii.
Answer: In Figure 4 we are not trying to compare Aegilops tauschii and bread wheat, we are highlighting the difference between expressed genes and non-expressed genes in both Aegilops tauschii and bread

wheat (see text below). As such we feel that it is important to keep the information separate. Furthermore, having trialled combining parts A and B we feel that the plot looks too busy as the lines overlap. The text has been modified to read: “Fig. 4 shows a decrease of CpG methylation at the Transcriptional Start Site (TSS) and Transcription Termination Site (TTS) compared to the promoter and gene body regions. This pattern of reduced TSS methylation of CpG and CHH contexts is more marked for expressed genes than non-expressed genes in both 3L and 3DL. CpG and CHG methylation at the TSS (+/- 20bp) is significantly lower in expressed genes compared to non-expressed genes (CpG sites; $p< 0.0001$, $t=5.9739$, $df=80$, CHG sites; $p< 0.0001$, $t=6.3446$, $df=80$).”

Question 6. In the part of "Comparative DNA Methylation", DNA methylation at gene body and promoters were performed by exome capture and then bisulfite sequencing, but the whole-genome bisulfite sequencing was performed to bread wheat. It is unclear why different methods were used in the comparison. Furthermore, it is also unclear how efficient the method of "exome capture" can capture promoter regions? Will it affect the comparison in promoter region?
Answer: Whole genome bisulfite sequencing was performed for bread wheat as part of a large-scale reference methylome project. Carrying out the same whole genome analysis for Ae. tauschii would have been prohibitively expensive, therefore we developed exome capture combined with bisulfite sequencing as previously described (Gardiner et al., 2015). In this previous analysis we show that a 6Mb capture probe set typically yielded coverage of 27 Mb of the reference genome as sequencing significantly extends out from the targeted sequence, which includes 5’ and 3’ flanking sequences. Therefore such targeting of the exome yielded a significant amount of promoter sequence that we use here. To allow comparison we state in the paper that “A gene/promoter region was only analysed if a minimum of 5 methylated cytosines were included in the region, each with a minimum bisulphite sequencing coverage of 5x for Ae. tauschii 3L and 10x for Paragon 3DL, as it had a higher sequence depth coverage. This identified 2,709 unique gene-regions and 2,182 unique promoter regions across the CpG/CHG/CHH contexts for chromosome 3DL and 2,952 unique gene-regions and 2,719 unique promoter regions for chromosome 3L.” Our approach means that we can only analyse the overlap between sequenced promoters for bread wheat and Ae. tauschii, however this results in “2,224 gene pairs in total (64.35%) across CpG/CHG/CHH gene and promoter sites (1,920 genes and 1,368 promoters)” for comparison, which is a significant number for us to draw confident comparative conclusions. The approach is described in: Gardiner, L.-J. et al. A genome-wide survey of DNA methylation in hexaploid wheat. Genome Biology 1–15 (2015). doi:10.1186/s13059-015-0838-3. To clarify this point we now state in the Methods section that: “The use of paired-end Illumina libraries, which extend beyond the exome capture baits allow sequencing and analysis of flanking regions of genes (Gardiner et al., 2015).”

Question 7. In the part of "Chromatin accessibility", the author claimed that "Of the 683 genes with differential ATAC peaks between hexaploid 3DL and diploid 3L, 133 of 159 genes (84%) that were differentially expressed in leaf tissue (the tissue used for ATAC-seq) also had differential ATAC peaks (Fig. 8; Additional File 3: Table S10). ". However, on page 10, it is "Of the 262 (16% of 1,564 expressed genes) leaf-specific DEGs identified in wheat 3DL and Ae. Tauschii AL8/8 3L, 106, were conserved in all three Ae. tauschii varieties.". Why the amount of DEGs in leaves are different in these two parts?
Answer: Not every gene had an ATAC-seq peak. Among 262 leaf DEGs, 159 genes had at least one ATAC-seq peak in either wheat or Ae tauschii or both. Those DEGs without ATAC-seq peaks were of course excluded from ATAC-seq analysis. We have clarified this point in the revised manuscript.
Question 8. In Figure 8, the authors showed that the overlap between DEGs and the genes with differential ATAC peaks in hexaploid 3DL and diploid 3L. The analysis here is too simple. The genes with increased expression and decreased expression must be divided into two groups, then the relationship between gene expression and chromatin accessibility can be analyzed.
Answer: Our data shows that having more open chromatin does not necessarily imply higher expression levels, and vice versa, as chromatin could for example be open to the activities of transcriptional repressors and activators. Therefore the relationships between having more open chromatin and higher expression, or having more closed chromatin and lower expression, are not direct. As suggested we tried dividing differentially expressed genes into those that are up in hexaploid or up in diploid but we could not see any trend in increased accessibility in wheat and increased expression, or increased expression in tauschii and increased expression. For this reason we wish to maintain the current version of Figure 7.
Question 9. Please add line number in the manuscript, which will be very useful in the reviewing process.
Answer: Line numbers have been added to the revised manuscript

Response to Reviewer 3:
Major comments:
Question 1. Lu et al. used TPM (transcript per million), as mentioned in the manuscript (page 21, line 9),

to normalize transcript abundance. But the manuscript didn't state clearly what 'per million' represent. Is it the total number of sequencing reads or the total mapped reads. It is likely from the context that it is per million mapped read, with read mapped to "the complete Triticum3.1 genome assembly with 3DL assemblies replaced by our chromosome 3DL pseudomolecule." Still, the readers have no idea about what the "Triticum3.1 genome assembly" is. No matter which way of normalization is selected, to use TPM to compare sequencing data from hexaploid and diploid is debatable. Assuming the following:
a) there is only one expressed gene in diploid and hexaploid subgenome D, both of which are syntenic. The corresponding syntenic genes in A and B are also expressed.
b) the true expression level of this gene is the same between diploid and across subgenomes of hexaploid (designated 1).
Then, the total number of mapped reads in diploid wheat is 1, and the total number of mapped reads in hexaploid wheat is 3. If normalized by TPM, the relative read density of diploid is 1/1 = 1, while the relative read density of hexaploid is 1/3 = 1/3. This is in contradict with previous assumption that the expression density of the orthologue is the same. The above situation is an ideal condition which didn't take into account the big difference between these two genomes. Despite the gene synteny is high in 3L region, using TPM to normalize transcript expression need to take into account the total mapped read, since the difference (death and birth of genes) between hexaploid and diploid could makes big difference of background for normalization.
Answer: 1) With respect to Triticum3.1, please refer to Reference [18], describing this PacBio Assembly of wheat. We used the PacBio sequence reads from this work and re-assembled them with BAC assemblies and re-annotated the assemblies.
2) We agree that using TPM comparison across species with different gene numbers may appear to be un-straightforward, which is why we validated the use of TPM using qRT-PCR to provide a measure of absolute transcript levels per cell (Figure 2C). The fold changes we see in this set of genes is consistent with the fold changes assessed by TPM. As TPM is a current standard measure of comparative RNAseq we think that our validation is relevant to the identification of differentially expressed genes. Figure 2A shows the overall differences in diploid chromosome 3L and hexaploid 3DL gene expression. genes, in which a hexploid-context 3DL gene is measured as 70% of the TPM values seen in diploid 3L. This is why we used 4-fold change to define differential genes rather than the usual 2-fold difference.
Question 2. To validate the TPM result, Lu. et al designed a qPCR experiment focusing on only 14 genes. However, there is no description throughout the text about what the different labels represent, and how the reader could conclude from Figure 2 that the qPCR result is consistent with TPM result. Still, given that the assumption under TPM normalization is controversial, even if the qPCR results for several genes are consistent with TPM, whether the conclusion could be driven is still questionable.
Answer:
The qPCR methods can be found in Transcription Analyses in the Methods section, and details about qRT-PCR can be found in Additional File 5.
The labels on the X axis of Figure 2 represent the identities of the selected 3DL genes assayed, and these are described in Additional File 4. The legend of Figure 2C has been expanded to provide further description.
We have also added text to the legend of Figure 2 explaining universal and specific primers. "Universal primers can amplify all the homoeologs while specific primers can only amplify DD genes." This is also explained in Transcription Analyses in the Methods section.
The 14 genes were selected for qRT-PCR analysis from non-differentially expressed genes. We say 40% reduction is an overall trend for the gene tested. The log2 scale of expression needs to be taken into account when relating Figure 2C to the RNAseq differences.
The information we wish to show Fig2C is, hexaploid AA+BB+DD (solid red circle) is comparable with diploid DD (solid blue rectangle).
Question 3. It is concluded that the length of DNA accessible loci correlated with differential expression. However, no clear description about how the accessible length is defined. The length is sensitive to the parameters selected when defining accessible loci. In addition, there is no appropriate control, and the genes need to be partitioned according to increase, decrease or no expression change in Aet, followed by examination of the accessible length distribution. I'm also confused how to quantify the length of accessible length within gene body and UTR. Using the length of overlapping region? It may be biased by the length of the gene or UTR.
A detailed description of ATAC-seq methods is provided in Additional File 1. Firstly, the open regions were classified into 4 categories (Intergenic, CDS+Introns, UTR5 Promoters, UTR3 + Downstream). These genic regions were defined according to our precise gene annotations. Secondly, we calculated the total length (X) of open regions for each category. This was based on the length of mapped ATAC reads for each genic region. Thirdly, for each category, we placed open chromatin regions into 40bp bins based on their length, as determined by PE reads from ATAC seq. For example, all the regions with length >=121 and <=160, were put in one bin. Then we calculated the total length for each bin, which gave

bin length (Bin40, Bin80, Bin120, and so on). Fourth, these bin lengths were divided by category total length (X). Finally, the divided values were plotted as heatmap using R. This method is described in Additional File 1.
With respect to controls, we used naked genomic DNA as an ATAC template. This is commonly used as essentially every genomic region is equally accessible to the Nextera reagent. Additional File 1 describes how MACS2 was used to call peaks and to merge them if they were less than 10bp apart.
Question 4. There is no description about whether there is biological replicates.
Answer: RNAseq, DNA methylation and ATAC-seq were all conducted using three biological replicates. RNAseq replicates could be found in Table S3 in Additional File 3. The ATAC-seq replicates could be found in Additional File 1. All the replicates have been submitted to ENA.
Question 5. Since both genome sequence and transcriptome of Chinese Spring is published, why not compare the transcriptome of syntenic genes between Chinese Spring and the diploid?
Answer: Access to the IWGSC whole genome wheat assembly was not possible when we initiated this study, whereas we had access to our own Paragon genome assembly and detailed transcriptional analyses, with a high quality annotation based on Isoseq and RNAseq resources. There are advantages to using Paragon, as it is an elite wheat accession that is widely used in academic researchers as a functional genomics resource. This means we can build on the results we have made using these functional genomics resources, such as large deletions. Furthermore, its genome is much more representative of modern wheat cultivars than the Chinese Spring landrace.
Question 6. What’s the difference between proportionally reduced genes and differentially expressed genes? “DEGs were categorized by normalizing wheat TPM values to Ae. Tauschii TPM values by multiplying them by 1.7604”, why multiplying 1.7604 after TPM? Where does 1.7604 come from?
Answer: Proportionally reduced genes refer to the approximately 70% of genes that show an overall pattern of reduction to about 40% when in the hexaploid compared to diploid contexts. DEGs were conservatively defined by a 4-fold TPM change after normalization using the 1.7604 factor was calculated by a median-normalization. This point has been clarified in the text. As hexaploid TPMs were generally lower (the 40% reduction) than diploid Ae tauschii TPM, we think it is prudent to use a relatively conservative 4-fold difference to define DEGs.
Question 7. The authors used the difference in exon-intron structure to find the relationship between divergence of promoter/gene sequences and different patterns of gene expression , why?
Answer: It is well-known that alternative splicing affects the expression levels of different RNA isoforms, therefore we included these types of predicted functional variants in our definition of gene divergence. In fact there were relatively few examples. We want to convey the information that despite a high degree of DNA sequence and methylation similarity between 3L and 3DL genes, their expression levels are highly different. We make a case for chromatin- level changes as correlated changes that may underlie these differences.
Minor:
There are many details missing for both results and methods section. For example
Question 1. What are the numerical value represent for in the Additional file 6, I cannot get information about the “86 DEGs on 3DL that were up-regulated in 27 DAP developing grain of wheat compared to Ae. tauschii‟.
Answer:
In Additional File 6, the numbers are percentage of genes involved in that category based on GO annotation and classification by the online WEGO plotting tool (methods in Addition File 1)
The number of DEGs were summarized in Table S4 in Additional File 3. The detailed gene information could be found in Additional File 4. Please refer to column names: ‘DEG’ and ‘test_DEG’. The numbers in ‘DEG’ columns indicate fold change (upregulated in wheat by positive values and down-regulated by minus values). Those genes with absolute value >=4 is DEGs. test_DEG columns indicate whether the gene is a gene (number 1) or not (number 0).
Question 2. How to normalize peak length in Fig. 6A?
Answer: Firstly, the open regions were classified into 4 categories (Intergenic, CDS+Introns, UTR5 + Promoters, UTR3 + Downstream). Secondly, we calculated the total length (X) of open regions for each category. Thirdly, for each category, we placed open chromatin regions into 40bp bins based on their length, as determined by PE reads from ATAC seq. For example, all the regions with length >=121 and <=160, were put in one bin. Then we calculated the total length for each bin, which gave bin length (Bin40, Bin80, Bin120, and so on). Fourth, these bin lengths were divided by category total length (X). Finally, the divided values were plotted as heatmap using R. This method is described in Additional File 1.
Question 3. What is the percent of annotated repeats on the chromosome arms? Is SRR in the largest proportion?
Answer: The repeat information is summarized in Table S2 in Additional File 3. Around 70% of chromosome arms were annotated as repeats, including class I and II transposons (64%), as well as

SSRs (0.4%). The largest portion is class II LTR transposons (51.4%). Interestingly, almost each intergenic open region intersected with a repeat feature contains a SSR. Some published papers reported nucleosome wrapped by SSR were depleted in histone proteins, thus exposing DNA for ATAC seq. But only 8% of SSRs had open chromatin in our analyses..
Question 4. In Fig8, why do authors use DEG and differential peaks to make the venn plot, but not up/down genes and short/long peaks?
Answer: We addressed this in our responses to major Question 3.
Question 5. Fig.6C used peak distance to TSS to represent peak length, which is strange.
Answer: This is a figure to describe the peak distribution related to a generalised gene, plotted by the ChIPseeker program. It does not represent the peak length. It indicates almost half of the ATAC peaks were within 3kb of upstream/downstream of genes.

Close
