# Supplementary material for: Reduced chromatin accessibility underlies gene expression differences in homologous chromosome arms of diploid Aegilops tauschii and hexaploid wheat
Source: Gigascience. 2020 Jun 20;9(6):giaa070. doi: 10.1093/gigascience/giaa070 (PMC7305686; doi:10.1093/gigascience/giaa070)
Supplement: giaa070_Reviewer_1_Report_Original_Submission — Z. Jeffrey Chen -- 12/4/2019 Reviewed [file giaa070_reviewer_1_report_original_submission.pdf]

**Reviewer Report**

**Title: Reduced chromatin accessibility underlies gene expression differences in homologous chromosome arms of diploid Aegilops tauschii and hexaploid wheat**

**Version: Original Submission** **Date:** 12/4/2019

**Reviewer name: Z. Jeffrey Chen**

**Reviewer Comments to Author:**

Lu et al. reported a study on gene expression changes in the long arm of chromosome 3D (3DL) relative to 3L in the extant D-genome diploid (Ae. tauschii). They found high syntenic relationship between these two chromosomal arms with 99% identity in the genic regions (3,456 of 3927 predicted genes). Approximately ~70% of genes in 3DL showed ~40% reduction of expression levels, relative to those in 3L. They further showed methylation difference accounting for ~10% of DEGs. In addition, ATAC-seq indicated that chromatin accessibility correlated with 86% (133 of 158) genes in the leaf tissue. They concluded that chromatin structure and organization of the hexaploid wheat may contribute to differential expression (reduction) of the genes in the homoeologous D chromosomes relative to the D-genome diploids.

Polyploidy is an important evolutionary feature of genome evolution for all flowering plants including many crops such as wheat. However, wheat genome is too large to conduct extensive analysis of DNA methylation and some other genome-wide studies. This study using a pair of chromosome arms has provided some valuable insights into roles of DNA methylation and chromatin accessibility in gene expression variation in the hexaploid wheat. Having that said, some data interpretation and conclusions should be revised to improve clarity and impact.

(1) Non-syntenic genes (Fig. 1): Most of the 207 non-syntenic genes (~5%) are located near the chromosome end. It is unclear how the non-syntenic genes are defined, compared to 3,456 orthologous genes with >99% identity. What is the possible reason for those non-syntenic genes predominantly in the chromosome ends? Is this a general feature for all other chromosomes or the entire hexaploid genome?

(2) Gene expression (Fig. 2): "In total, 2,375 (68.72%) of the syntenic genes from 3DL (1,893 genes) and/or 3L (2,217 genes) were expressed at TPMâ‰¥1 in these tissues (Additional File 4)." It is unclear what is meant, and how many genes (%) are expressed, relative to what? Again, in the following sentence, "…showed a significant trend in reduction of TPM values in the hexaploid context to approximately 40% of those measured in diploid genomic context for approximately 70% of the pairs of genes." If these are syntenic (orthologous) genes, shouldn't they have the same number of denominators for expressed genes in both 3D and 3L, unless some genes are expressed specifically in 3D or 3L, which should be excluded for this analysis and discussed separately.

(3) DEGs (Fig. 2): It is unclear how this normalization of the transcriptome in 3L by multiplying "1.7604" is defined and justified. Although 70% of genes in 3D could be expressed at lower levels than those in 3L, expression of homoeologs in 3A and 3B is not examined and could be upregulated to compensate as a dosage effect, as every D homoeolog in hexaploid wheat would have additional two other homoeologs.

This section of DEGs should be revisited. On a related note, image displays in Fig. 2B, D are confusing with too many lines and colors and should be simplified to highlight the DEGs.
They examined gene expression in 5 different tissues. Is this reduction of expression in the leaf consistent across all five tissues or different among tissue types? They reported 86 DEGs (upregulated in 3DL) during grain development. Thus, down-regulation of some genes in 3DL is not a common phenomenon and may vary during development. What about other tissues?
(4) Gene expression vs. sequence diversity (Fig. 3): this type of data should be in the supplemental information.
(5) DNA methylation changes (Fig. 4). The separate images (expressed vs. non-expressed genes) in 3DL and 3L do not convey a clear message. It would make a bit more sense to display methylation profiles with DEGs between 3DL and 3L, using all genes (or genes with equal expression levels) as the control. The images can be enlarged. The panels should be labeled as "A" and "B" (instead of "a" and "b").
"Only 11.3 % of differentially methylated genes or promoters were associated with differences in gene expression between Paragon 3DL and Ae. tauschii 3L (Table 1)." Replace "associated" with "correlated" (with gene expression differences between…). Based on this number, though small, the methylation seems to play a role. Some details are unknown here, e.g., location of methylation in 5', 3' UTRs or gene body, because this would affect their data interpretation (positive, negative or uncorrelated). They do not seem to be consistent with what is stated. If methylation of DEGs was displayed, the picture would become clear?
A related comment is that they used Agilent SureSelect gene capture for methylation analysis, which may have problems of capturing efficiency and representation of the sequences. This should be discussed and taken into consideration for concluding their results.
(6) Chromatin accessibility (ATAC-seq) (Figs. 5 and 6). Data in Fig. 5 dealt with repeats and ATAC-seq peaks, which do not have much to do with gene expression and should be in the supplementary information.
Proportionally reduced expression vs. differentially expressed genes. It is unclear how these two groups of genes are defined. Based on results in Fig. 6, a main difference between 3DL and 3L is that the chromatin accessibility peaks are shorter in 3DL than in 3L. If one assumes close chromatin accessibility sites (to TSS) are important to gene expression, should the conclusion be opposite to what was noted in the manuscript? An important parameter is the peak intensity (or fold enrichment of peaks), which determines the chromatin accessibility, but this has not been examined, which could be a major problem for their overall conclusion.
A minor note: both "proportionally" and "proportionately" are interchangeably used in the Abstract and text. Should they use one form?
(7) Again in Fig. 7, it is unclear how the peak length correlates with expression of a gene (and see the comment above).
(8) Based on the data in Fig. 8, the correlation between chromatin accessibility and DEGs is not as straightforward as stated in the manuscript. Only 75 genes correlate with differential ATAC-seq peaks in UTRs, plus another 28 in 3' UTRs, which could be 5' UTRs of neighboring genes. They should revisit their conclusions (about relative contributions of DNA methylation and chromatin accessibility) in the Title, Abstract, and the rest of MS.

**Methods**

Are the methods appropriate to the aims of the study, are they well described, and are necessary controls included? Choose an item.

**Conclusions**

Are the conclusions adequately supported by the data shown? Choose an item.

**Reporting Standards**

Does the manuscript adhere to the journal's guidelines on minimum standards of reporting? Choose an item.

Choose an item.

**Statistics**

Are you able to assess all statistics in the manuscript, including the appropriateness of statistical tests used? Choose an item.

**Quality of Written English**

Please indicate the quality of language in the manuscript: Choose an item.

**Declaration of Competing Interests**

Please complete a declaration of competing interests, considering the following questions:

- Have you in the past five years received reimbursements, fees, funding, or salary from an organisation that may in any way gain or lose financially from the publication of this manuscript, either now or in the future?
- Do you hold any stocks or shares in an organisation that may in any way gain or lose financially from the publication of this manuscript, either now or in the future?
- Do you hold or are you currently applying for any patents relating to the content of the manuscript?
- Have you received reimbursements, fees, funding, or salary from an organization that holds or has applied for patents relating to the content of the manuscript?
- Do you have any other financial competing interests?
- Do you have any non-financial competing interests in relation to this paper?

If you can answer no to all of the above, write 'I declare that I have no competing interests' below. If your reply is yes to any, please give details below.

I declare that I have no competing interests

I agree to the open peer review policy of the journal. I understand that my name will be included on my report to the authors and, if the manuscript is accepted for publication, my named report including any attachments I upload will be posted on the website along with the authors' responses. I agree for my report to be made available under an Open Access Creative Commons CC-BY license (http://creativecommons.org/licenses/by/4.0/). I understand that any comments which I do not wish to be included in my named report can be included as confidential comments to the editors, which will not be published.

Choose an item.

To further support our reviewers, we have joined with Publons, where you can gain additional credit to further highlight your hard work (see: https://publons.com/journal/530/gigascience). On publication of this paper, your review will be automatically added to Publons, you can then choose whether or not to claim your Publons credit. I understand this statement.

Yes Choose an item.
