# Supplementary material for: Reduced chromatin accessibility underlies gene expression differences in homologous chromosome arms of diploid Aegilops tauschii and hexaploid wheat
Source: Gigascience. 2020 Jun 20;9(6):giaa070. doi: 10.1093/gigascience/giaa070 (PMC7305686; doi:10.1093/gigascience/giaa070)
Supplement: giaa070_Reviewer_2_Report_Original_Submission — Hua Jiang -- 12/5/2019 Reviewed [file giaa070_reviewer_2_report_original_submission.pdf]

Reviewer Report

Title: Reduced chromatin accessibility underlies gene expression differences in homologous chromosome arms of diploid Aegilops tauschii and hexaploid wheat

Version: Original Submission **Date:** 12/5/2019

Reviewer name: Hua Jiang

Reviewer Comments to Author:

Hexaploid bread wheat is one of the most important crops in the world, and the plasticity of the transcriptome in allopolyploidization is one of the core questions to understand the evolution of bread wheat. Currently, the mechanisms that induce transcriptome changes in this process is still not well understand. In this manuscript, Lu and the colleagues utilized Aegilops tauschii, the progenitor of wheat D chromosomes, to investigate the mechanisms underlie the transcriptomic plasticity in hexaploid wheat. They performed RNA-seq, bisulfite-seq, and ATAC-seq to investigate the relationship between gene expression levels, DNA methylation, and chromatin accessibility in wheat and Ae. tauschii. They concluded that chromatin compaction orchestrated reduced gene expression levels in hexaploid chromosomes of wheat compared to its diploid progenitor chromosomes. The biological question is essential, and the conclusion is interesting. Nevertheless, some places in the manuscript must be improved before publication.

Comments:

1. In the part of "Assay for Transposase-Accessible Chromatin (ATAC) Sequence Datasets," "ATAC-seq reads matching mitochondrial, and chloroplast genomes were identified and removed using the Triticum aestivum chloroplast genome (GenBank accession No. NC_002762), Triticum aestivum mitochondrial genome (GenBank accession No. AP008982) and the Ae. tauschii chloroplast genome (GenBank accession No. NC_022133). ". It is unclear why the ATAC-seq reads matching Ae. tauschii mitochondrial genome are not removed.
2. The RNA-seq data of roots, seedlings, and developing grains is not necessary, as the tissues used in bisulfite-seq and ATAC-seq are only leaves. Moreover, the author did not clarify whether the leave materials used in bisulfite-seq, ATAC-seq, and RNA-seq are completely the same, which is vital to analyze the relationship among three sets of data.
3. In Figure 3, DEGs have relatively less sequence similarity compared to non-DEGs. Statistical analysis should be performed to test if the difference is significant or not.
4. The author concludes that DNA methylation only plays a minor role in reduced gene expression in hexaploid wheat, but the analysis of bisulfite sequencing data is not perfect enough. First, the description of data analysis is not detailed enough. In part defining the differential methylated region, "DMRs were defined if a CpG region showed a difference in methylation of 50% or more ($q < 0.05$), a CHG region showed a difference of 25% or more or a CHH site showed a difference of 10% or more. There is no reference shown here. Is it possible that the definition of DMR is too strict, resulting in a poor relationship between reduced gene expression and the distribution of DMRs?
5. In figure 4, part A of bread wheat and B of Aegilops tauschii should merge into one part, and then

the difference between Aegilops tauschii and bread wheat will be easy to see. According to the result in figure 4, the levels of CHG and CHH methylation in the promoter and 3 UTR regions in bread wheat are lower than in Aegilops tauschii.

6. In the part of "Comparative DNA Methylation", DNA methylation at gene body and promoters were performed by exome capture and then bisulfite sequencing, but the whole-genome bisulfite sequencing was performed to bread wheat. It is unclear why different methods were used in the comparison. Furthermore, it is also unclear how efficient the method of "exome capture" can capture promoter regions? Will it affect the comparison in promoter region?
7. In the part of "Chromatin accessibility", the author claimed that "Of the 683 genes with differential ATAC peaks between hexaploid 3DL and diploid 3L, 133 of 159 genes (84%) that were differentially expressed in leaf tissue (the tissue used for ATAC-seq) also had differential ATAC peaks (Fig. 8; Additional File 3: Table S10). ". However, on page 10, it is "Of the 262 (16% of 1,564 expressed genes) leaf-specific DEGs identified in wheat 3DL and Ae. Tauschii AL8/8 3L, 106, were conserved in all three Ae. tauschii varieties.". Why the amount of DEGs in leaves are different in these two parts?
8. In figure 8, the authors showed that the overlap between DEGs and the genes with differential ATAC peaks in hexaploid 3DL and diploid 3L. The analysis here is too simple. The genes with increased expression and decreased expression must be divided into two groups, then the relationship between gene expression and chromatin accessibility cab be analyzed.
9. Please add line number in the manuscript, which will be very useful in the reviewing process.

Methods

Are the methods appropriate to the aims of the study, are they well described, and are necessary controls included? Choose an item.

Conclusions

Are the conclusions adequately supported by the data shown? Choose an item.

Reporting Standards

Does the manuscript adhere to the journal's guidelines on [minimum standards of reporting?]() Choose an item.

Choose an item.

Statistics

Are you able to assess all statistics in the manuscript, including the appropriateness of statistical tests used? Choose an item.

Quality of Written English

Please indicate the quality of language in the manuscript: Choose an item.

Declaration of Competing Interests

Please complete a declaration of competing interests, considering the following questions:

- Have you in the past five years received reimbursements, fees, funding, or salary from an organisation that may in any way gain or lose financially from the publication of this manuscript, either now or in the future?
- Do you hold any stocks or shares in an organisation that may in any way gain or lose financially from the publication of this manuscript, either now or in the future?
- Do you hold or are you currently applying for any patents relating to the content of the manuscript?
- Have you received reimbursements, fees, funding, or salary from an organization that holds or has applied for patents relating to the content of the manuscript?
- Do you have any other financial competing interests?
- Do you have any non-financial competing interests in relation to this paper?

If you can answer no to all of the above, write 'I declare that I have no competing interests' below. If your reply is yes to any, please give details below.

I declare that I have no competing interests

I agree to the open peer review policy of the journal. I understand that my name will be included on my report to the authors and, if the manuscript is accepted for publication, my named report including any attachments I upload will be posted on the website along with the authors' responses. I agree for my report to be made available under an Open Access Creative Commons CC-BY license (http://creativecommons.org/licenses/by/4.0/). I understand that any comments which I do not wish to be included in my named report can be included as confidential comments to the editors, which will not be published.

Choose an item.

To further support our reviewers, we have joined with Publons, where you can gain additional credit to further highlight your hard work (see: https://publons.com/journal/530/gigascience). On publication of this paper, your review will be automatically added to Publons, you can then choose whether or not to claim your Publons credit. I understand this statement.

Yes Choose an item.
